# Stringent selection drives convergence toward omicron-like SARS-CoV-2 receptor-binding motifs

**DOI:** 10.1038/s41467-026-72312-z

**Published:** 2026-04-25

**Authors:** Aviv Shoshany, Ruojin Tian, Miguel Padilla-Blanco, Adam Hruška, Aditi Konar, Katarína Baxová, Eyal Zoler, Martin Mokrejš, Gideon Schreiber, Jiří Zahradník

**Affiliations:** 1 Department of Biomolecular Sciences, Weizmann Institute of Science, Rehovot, Israel; 2 First Faculty of Medicine, Charles University, BIOCEV center, Vestec, Czechia; 3 Viral Immunology Lab, Molecular Biomedicine Department, Margarita Salas Center for Biological Research (CIB-CSIC), Madrid, Spain; 4 Institute of Organic Chemistry and Biochemistry, Czech Academy of Sciences, Prague, Czechia; 5 Institute of Biotechnology, Czech Academy of Sciences, BIOCEV center, Vestec, Czechia

**Keywords:** SARS-CoV-2, Molecular evolution, Phylogenetics

## Abstract

In vitro protein evolution can provide powerful insights into the amino acid sequences that underlie key biological functions. Here, we use this to explore the evolutionary trajectories of the SARS-CoV-2 spike protein receptor-binding motif (RBM) binding the human angiotensin-converting enzyme 2 (ACE2), an essential first step in viral infection. Applying stringent selection pressures starting from the Wuhan or another non-Omicron variant protein-coding sequence results in rapid convergence towards Omicron characteristic mutations and its sub-lineages. Conversely, under mild selection, only some Omicron-like mutations are selected, however at lower frequencies and with incomplete representation. Stringent selection results in fewer, but dominant, non-synonymous mutations mirroring Omicron mutations and their variations within its sub-lineages. Notably, initiating evolution from Omicron itself results in maintenance of Omicron-defining mutations under both conditions. This evolutionary pattern parallels global SARS-CoV-2 mutation trends as well as in silico simulations, emphasizing the critical role of receptor-binding constraints in shaping viral adaptation. Mutations primarily associated with immune evasion are not selected by in vitro evolution. Our findings demonstrate the predictive capacity of in vitro evolution, suggesting Omicron RBM to be the humanized binding motif, emerging from high-stringency selection, superimposed on milder background pressures.

## Abstract (web summary)

Authors explore the in vitro evolution of the binding elements on SARS-CoV-2. They find that stringent but not mild laboratory evolution led to rapid convergence toward Omicron lineage mutational patterns.

## Introduction

The emergence of SARS-CoV-2 in late 2019 initiated a global pandemic with profound impacts on public health. The initial Wuhan strain was rapidly replaced by successive viral lineages with higher fitness. Viral mutations are accumulated and selected during the virus life cycle, with most mutations being lost due to strong selection during the tight bottlenecks imposed^1,2^. Transmissibility, usually expressed as the net reproduction number (*R*t), is assumed to closely approximate their fitness at the host population level^3^. The mutation rate of SARS-CoV-2 is estimated to be 10^–6^ mutations per nucleotide per replication cycle, similar to other beta coronaviruses^4^. Yet, the distribution of mutations is not uniform and is highly context-dependent, influenced by local sequence composition, genomic region, and RNA structure and refined by functional selection^5^. In addition, frequent C → U mutations associated with host APOBEC enzyme account in part for the strikingly high ratio of non-synonymous changes in SARS-CoV-2 genomes compared with those at synonymous sites^6,7^. Due to the randomness of mutation events, single-nucleotide mutations dominate the early phases of the pandemic, which allows for substitution to 7–10 other amino-acids, as indeed observed for SARS-CoV-2. Double nucleotide mutations and epistatic mutations emerged gradually later^8^.

Central to SARS-CoV-2 transmission and pathogenesis is the Spike (S) protein, which underwent rapid adaptive evolution in the human population, shaping the trajectory of the pandemic^9–11^. The S protein consists of two subunits: S1, which contains the receptor binding domain (RBD, amino acids 331–528) and S2, responsible for membrane fusion^12^. The RBD directly interacts through its receptor binding motif (RBM, amino acids 438 to 506) with ACE2 of the host cell, making it critical for viral entry and a primary target for neutralizing antibodies^13^. The RBD has become a focal point of research as its structure and function are intricately tied to viral fitness, transmissibility, and immune escape. Molecular studies have revealed that even subtle modifications in the RBD, such as found in the Alpha, Beta, Gamma and Delta variants, can significantly alter viral transmission efficiency and host cell tropism^14,15^. Its adaptive evolution underlies the emergence of high-fitness variants, most of which have been designated by the WHO as Variants of Concern (VOCs) or variants of interest (VOIs) and have been closely monitored^16^. Omicron, which evolved at the end of 2021, was the most radical alteration of the RBD, and since then all VOCs are of the Omicron lineage. The emergence of the Omicron lineage marked a pivotal shift in the pandemic’s trajectory. Omicron displayed an unprecedented number of over 30 mutations in the Spike protein alone (15 of them in the RBD)^13,17^, which altered its interaction with ACE2 and neutralizing antibodies^13,18^. These mutations enhanced its ability to evade pre-existing immunity from vaccination and prior infections, contributing to widespread breakthrough infections. Omicron also demonstrated increased transmissibility, which, alongside immune escape, raised questions about its origins. Theories regarding Omicron’s evolution include prolonged infection in an immunocompromised host, facilitating the accumulation of mutations, or cross-species transmission, followed by adaptive evolution in an animal reservoir before re-entering the human population^3^. The mutations in Omicron altered the virus’s behavior, including a shift from TMPRSS2-dependent entry to preferential entry via the endosomal pathway^12,19^. This change potentially modified the virus’s tissue tropism, favoring the upper respiratory tract over the lower respiratory tract, which may have contributed to increased transmissibility but decreased pathogenicity^11^. Despite significant progress in characterizing Omicron’s genomic and phenotypic properties, the precise evolutionary pathway remains an enigma.

Selective pressures on the SARS-CoV-2 RBD are multifactorial, involving a balance between ACE2 binding affinity, immune evasion, and structural stability. Studies have highlighted the role of ACE2 affinity in viral fitness, with variants exhibiting increased ACE2 binding often demonstrating enhanced transmissibility^15,20^. This relationship is modulated by compensatory mutations that balance the competing demands of immune evasion and structural integrity. For example, the Omicron variant harbors key mutations in the ACE2 binding region—such as K417N, S477N, E484A, Q493R, G496S, Q498R, N501Y, and Y505H—that enhance ACE2 binding and/or immune evasion. Other mutations like G339D, S373P, S375F, N440K and T478K, while not directly interacting with ACE2, are frequently found in Omicron variants. Multiple studies showed the trend of increasing binding throughout the SARS-CoV-2 evolution^20,21^. In addition, deep mutational scanning studies have shown that mutations at protein-protein interfaces rarely have neutral effects and often influence binding properties, demonstrating the complexity of immune escape mechanisms^22–24^. While numerous studies have explored RBD evolution in the context of immune escape, the precise interplay of selective pressures shaping these mutations remains incompletely understood, highlighting the need for alternative approaches to deconvolute these forces.

In a previous study, affinity maturation of the SARS-CoV-2 RBD towards ACE2 resulted in a mutant RBD with 600-fold increase in binding affinity. This work demonstrated the rapid selection of affinity-enhancing mutations observed in the viral population^25^. Notably, we quickly acquired the mutations S477N, Q498R, and N501Y, which form part of the Omicron BA.1 variant^13^. However, that study had certain limitations as its primary objective was developing an infection inhibitor^26^ rather than studying evolutionary processes. First, the codons were optimized for expression in *Saccharomyces cerevisiae* for yeast display, meaning that single-point mutations could result in different amino acids than those derived from the original sequence. Second, the in vitro evolution included a pre-equilibrium selection step favoring faster on-rates, which may not align with natural viral evolution.

In this work we lay a robust foundation for using in vitro evolution to predict natural viral evolution. We provide evidence that the key mutations defining the Omicron lineage RBMs are primarily explained by adaptation to the human ACE2 receptor ortholog and stability maturation. Moreover, comparing low-stringency with high-stringency selection, combined with fitness/reward modeling, elucidates how mutations with differing effects on fitness behave under varying selective pressures. As a large proportion of modern epidemics are caused by zoonosis and adaptation to the orthologous receptor, these insights lay the groundwork for more robust predictions of viral variant emergence and evolutionary trajectories, showing a limited number of tipping points in the adaptive trajectory.

## Results

### Experimental design and setup for parallel in vitro evolution

In this study, we evaluated the sequence evolution of the RBM under selective pressures for ACE2 binding affinity and protein stability in an in vitro system without the neutralizing antibodies selection. Our aim was to determine how these pressures influence mutational outcomes across multiple starting viral lineage RBMs, and whether such pressures lead to convergent or divergent mutational trajectories that resemble those observed in SARS-CoV-2 evolution.

**Fig. 1:**
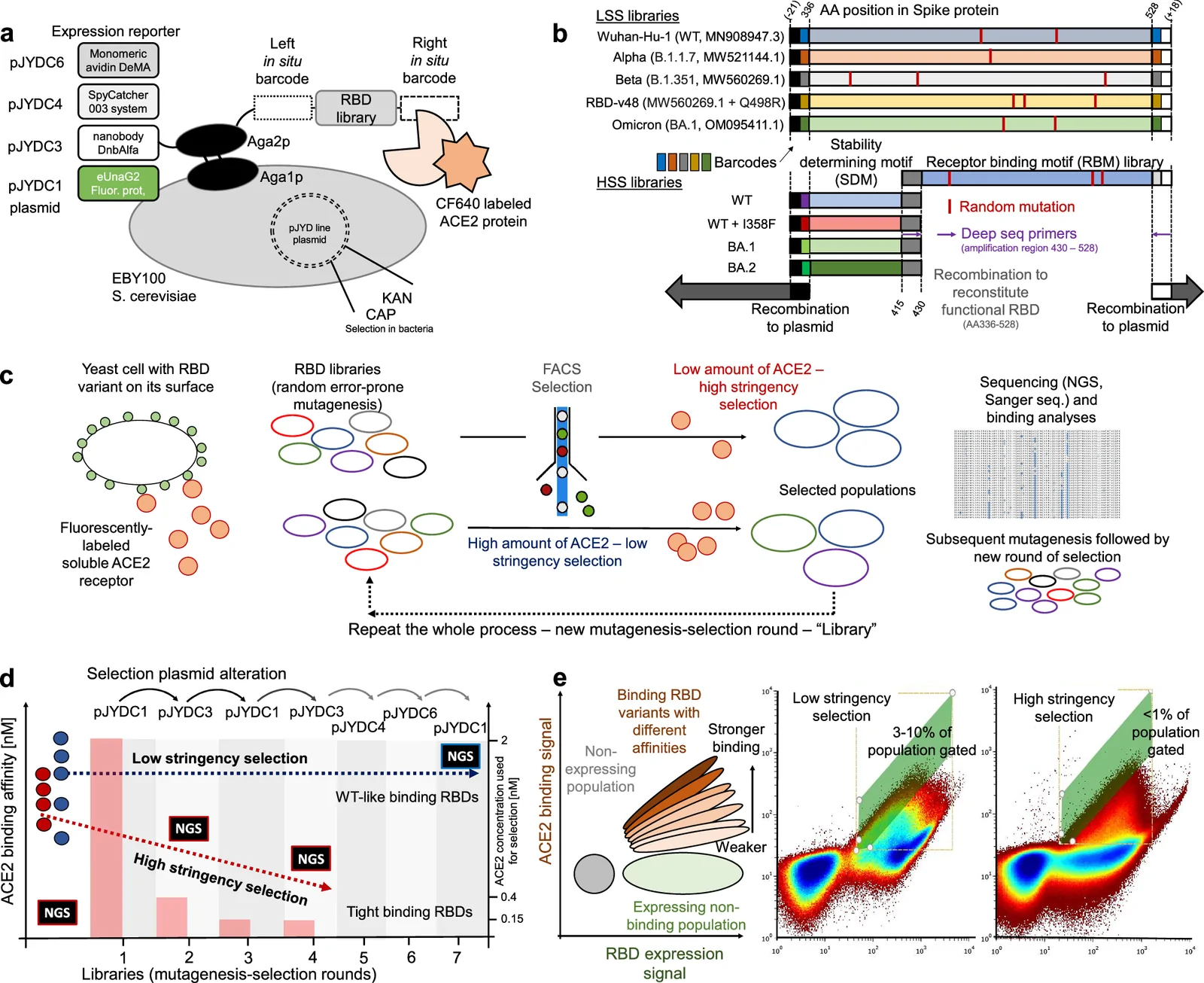
High-throughput yeast display evolution of SARS-CoV-2 RBD under defined selection pressures. **a** Schematic overview of the yeast display system used for in vitro evolution of the SARS-CoV-2 receptor-binding domain (RBD; S protein residues 331–528). Yeast cells express the RBD on their surface, enabling interaction with fluorescently labelled angiotensin converting enzyme 2 (ACE2) peptidase domain. Surface expression is monitored via orthogonal fluorescent reporter systems encoded on different plasmids. In situ barcodes and antibiotic markers allow precise identification of selected clones. **b** RBD library constructs designed for low-stringency selection (LSS) and high-stringency selection (HSS) regimes. In the LSS libraries, the entire functional RBD domain was subjected to mutagenesis, whereas in the HSS libraries, only the receptor-binding motif (RBM) region was diversified. In both cases, the mutagenesis rate was adjusted to yield approximately 1–3 mutations per variant. **c** Overview of the experimental workflow. Mutation libraries are subjected to FACS-based selection, with ACE2 concentration and sorting thresholds defining the selection regime. Selected and unselected populations are analyzed by Sanger and next-generation sequencing and can be further mutagenized, re-cloned into alternative plasmids, and reintroduced for subsequent rounds of evolution. **d** Framework for HSS and LSS mutagenesis-selection cycles, highlighting the use of distinct plasmid alterations to mitigate the risk of cross-contamination between libraries and respective evolutionary pathways. Constant or decreasing concentrations of ACE2 protein were used for LSS and HSS, respectively. The next-generation sequencing (NGS) symbol highlights the libraries that were subjected to deep sequencing analysis. Other stages were monitored by Sanger sequencing. **e** Scheme (left panel) and Fluorescence-Activated Cell Sorting (FACS) density dot plots using different gating strategies for LSS selection (middle panel) and HSS selection (right panel, for details see Fig. S1).

To monitor mutational pathways, we established a high-throughput, in vitro evolution system based on yeast surface display. In this system, multiple copies of single RBD domain variants are expressed on the surface of *Saccharomyces cerevisiae* EBY100 based on Aga1p-Aga2p interaction, allowing us to assess both surface expression levels and binding to soluble fluorescently labeled ACE2 (Fig. 1a-c). Surface expression is quantified using green fluorescent reporter systems fused to the displayed protein, while ACE2 binding is monitored using far-red labeled ACE2 protein. This system includes multiple improvements to enhance performance and scalability of the enhanced yeast display system^27^. To accurately identify the maximum number of clones from individual libraries throughout multiple rounds of in vitro evolutions, we extended our original strategy, which employed an enhanced yeast surface-display protocol incorporating two distinct surface expression detection strategies: a bilirubin dependent green fluorescent protein eUnaG2 and a nanobody recognizing ALFA-peptide with high affinity and specificity (DnbALFA)^27^. We introduced additional orthogonal detection systems: the SpyTag–SpyCatcher003 system for covalent isopeptide-bond-based expression labeling (pJYDC4)^28^, and designed stability and expression enhanced monomeric avidin based on rhizavidin for high affinity FITC-biotin based labeling (DeMA; Supporting information part PS1, pJYDC6^29^). In addition to orthogonal expression labeling strategies, we made an alternative plasmid encoding for chloramphenicol resistance and incorporated barcodes at both the N- and C-gene termini ensuring library stability throughout in vitro evolution. The N-terminal barcode was added as an extension of the existing (GGGS)_4_ linker within the expression construct. To prevent any potential interference with yeast display performance, we tested the barcodes using the expression of 3EFR-Cfr-Anti-Streptavidin binding protein^27^ and measured its binding to Streptavidin-APC (Table S1). Overall, our system consists of ten barcodes, four labeling strategies, and two selection antibiotics (Fig. 1a and b), representing a scalable and reliable framework for parallel in vitro evolutionary studies with minimal risk of cross-contamination or library loss due to unwanted impurities which can now be precisely identified. This approach minimizes the risk that the observed mutations arise from cross-contamination instead of genuine in vitro evolutionary processes.

### Exploring mutational trajectories under varying selective pressures

We adopted a strategy with a high degree of redundancy, characterized by starting with RBD sequences of different lineages and two distinct sorting strategies (Fig. 1a-c). The first strategy represents a low stringency selection (LSS), designed to mimic conditions where selective pressure maintains the initial binding affinity. This is achieved using Fluorescence-activated Cell Sorting (FACS) by selecting for 3–10% of the RBD expressing population and a bait (ACE2) concentration of 2 nM ACE2 (which is ~EC25 of WT). Each library underwent 7 cycles of random mutagenesis, with each cycle including two rounds of FACS sorting against ACE2 (Figs. 1d, S1). These permissive conditions allow mutations that slightly reduce binding affinity and protein stability to persist within the population. This strategy was applied in parallel to five different lineage-derived RBD sequences using error-prone mutagenesis covering the full RBD region (Fig. 1b). The starting RBDs originated from SARS-CoV-2 variants: Wuhan-Hu-1 (WT), Alpha (mutation present N501Y), Beta (K417N, E484K, N501Y), RBD variant 48 (RBD-v48, Beta + Q498R mutation^25^), and Omicron BA.1 (RBD-v48 + G339D, S371L, S373P, S375F, N440K, G446S, S477N, T478K, E484A, Q493R, G496S, Y505H) (Table S2).

The second strategy employed high-stringency selection (HSS), imposing stringent requirements to enhance binding affinity and maintain thermal-stability. Each cycle included random mutagenesis, heating of the yeast cells to 40 °C for 20 min (removing less stable mutants)^30,31^ and three rounds of FACS selection for the top 1% of ACE2-binders by clones. The first cycle of randomly mutated RBDs was selected against 2 nM of ACE2, followed by 0.4 nM ACE2, and finally 0.15 nM ACE2. In addition to WT and WT + I358F (a mutation that increases thermal-stability by 4 °C, Fig. S2c, allowing exploration of additional evolutionary trajectories^25^), the RBDs of BA.1 and BA.2 lineages were used as starting sequences for in vitro evolution (Table S2). To increase the likelihood of isolating high-affinity clones and comprehensively covering potential variants, error-prone mutagenesis was restricted to the receptor-binding motif (RBM, AA430–528, Fig. 1c), while residues 337-430, which do not participate in ACE2 binding, were fixed from the original variant to keep their stability and yeast surface expression constraints (Fig. S2).

**Fig. 2:**
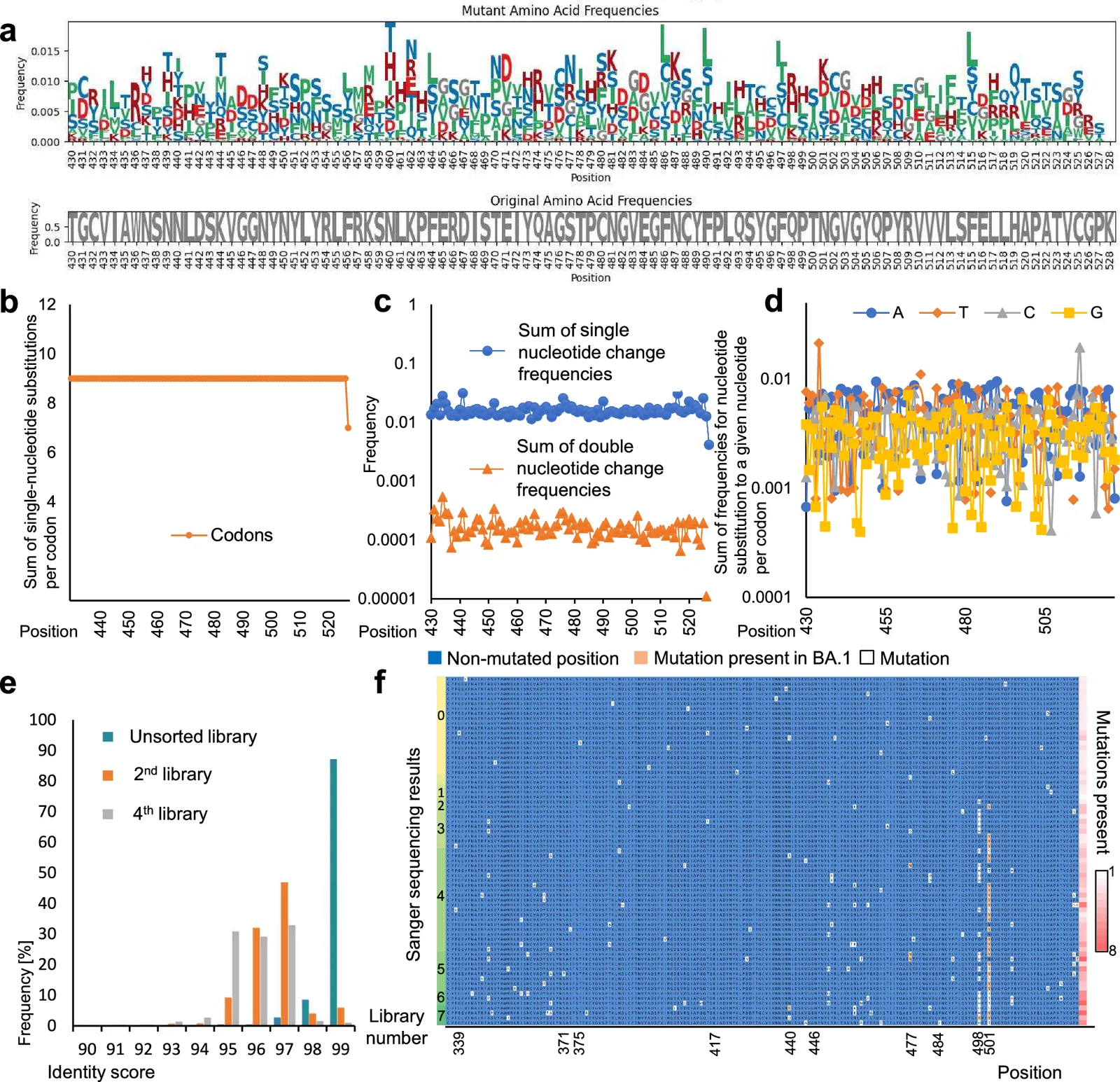
WT library quality assessment. **a** Sequence Logo plot displaying amino-acid frequencies in the first library for high-stringency selection based on SARS-CoV-2 WT receptor binding domain (HSS WT RBD), prior to selections. **b** The number of observed single nucleotide substitutions per codon along the RBM, with 9 representing all mutations in all three positions. **c** Frequencies for all single- and double-nucleotide substitutions per codon, showing minimal positional deviations. **d** Sum of single-nucleotide mutation frequencies per mutant nucleotide and codon. **e** Comparison of sequence similarity scores of individual merged paired-end reads into a single sequence with their respective reference (WT RBD sequence for the unsorted first library), showing increasing number of mutations accumulated during progression of in vitro evolution. More detailed distribution of nucleotide sequence identities of LSS and HSS libraries when aligned to the reference WT sequence is shown in Fig. S7. **f** Multiple sequence alignment of randomly sampled unique clones of LSS WT libraries (unsorted to 7th library, yellow-green segments) analyzed by Sanger sequencing, showing a gradual increase in the number of mutations (right white-red bar). The wild-type residues are shown as blue squares, the mutant residues presented in BA.1 are shown as orange squares and other mutations in white. Positions of important residues (BA.1/BA.2) are marked. Detailed analyses for LSS and HSS libraries are provided in Supporting information Figs. S3–S13. Source data are provided in the file Source_Data_Fig-2.xlsx.

Error-prone mutagenesis was experimentally optimized to predominantly generate sequences with a single mutation per gene. Selected libraries (Fig. 1d) were analyzed by next generation sequencing (NGS-Illumina paired-end deep sequencing 2 × 250 bp) while all rounds were monitored by Sanger sequencing. This combined strategy ensures that both high-frequency and relevant co-occurring mutations are accurately captured, while low-frequency variants are considered in the NGS analysis. A two-level barcoding system was employed to process the NGS data, incorporating additional barcodes into amplicons that already contained in situ barcodes embedded within the in vitro evolution plasmids (Fig. 1a, b and Tables S1 and S3). This dual barcoding strategy enabled robust and unambiguous identification of clones originating from distinct libraries and also sorting rounds, ensuring high fidelity in tracking evolutionary trajectories. Figure 2 shows an in-depth analysis of the WT library used for HSS. Figure 2a shows the sequence Logo plot as determined from NGS, displaying mutation frequencies prior to any selection. Figure 2b is an analysis of single-nucleotide substitution coverage in the random library prior to selection at specific positions, showing the presence of all theoretically expected mutations across nearly the entire gene length. Figure 2c shows the sum of frequencies for all single- and double-nucleotide substitutions in the starting library, indicating a homogeneous distribution of frequencies with minimal positional deviations. The frequency of double-nucleotide substitutions is 1/100 relative to single nucleotide substitutions, showing the rarity of double-nucleotide mutations, reflecting known observations from viral evolution^3,8,13^. Figure 2d sums up the single-nucleotide mutation frequencies per mutant nucleotide and position, showing an equal distribution. Overall, Fig. 2a-d shows the high quality of the starting library before selection. Figure 2e provides a snapshot of the number of mutations per sequence (similarity score relative to WT) before selection and after two and four rounds of in vitro evolution. While single-nucleotide mutations dominate the starting library, in vitro evolution results in higher numbers of mutations per sequence (with the highest number after the 4th library, Fig. 2e). A complete analysis of the mutant libraries for WT + I358F, BA.1 and BA.2 following the same scheme as shown for WT in Fig. 2a-e is given in Fig. S3-S7 (with WT as reference). The high frequency mutations seen in Figs. S4 and S5 are from Omicron as a starting variant, plotted relative to WT. Figure 2f shows the results of Sanger sequencing of the LSS WT library, showing a gradual increase in the number of mutations in individual sequences upon increasing rounds of in vitro evolution, from 1 to 7 (details for other libraries and all rounds of in vitro evolution, Figs. S8–S13). For deep sequencing analysis, we selected the unsorted, 2nd, and 4th rounds of HSS. For comparison, the 7th round of LSS was chosen to obtain a comparable number of mutations in the selected sequences as can be seen from Sanger sequencing results and Illumina sequencing reads analyses (Figs. S7–S18).

### Selection stringency dictates the outcome of in vitro evolution

**Fig. 3:**
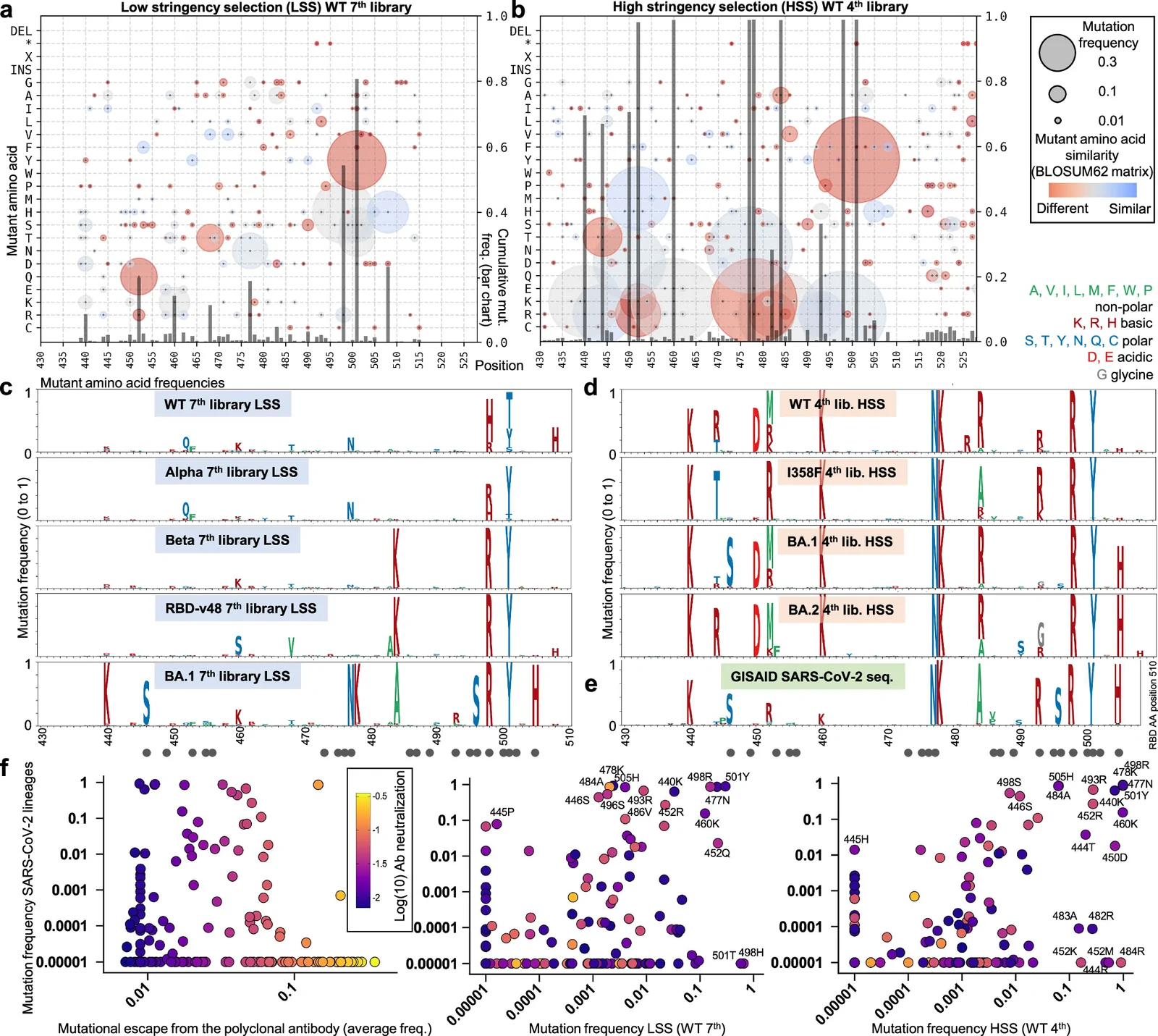
Mutation accumulation following in vitro evolution in comparison to SARS-CoV-2. Mutation scatter plots of in vitro evolution under low-stringency selection (LSS) **a** and high-stringency selection (HSS) **b**. The relative sizes of circles reflect mutation frequencies in the population, and color denotes amino acid similarity to the wild type based on the relative substitution score within the substitution matrix BLOSUM62^84^. The bars in (**a** and **b**) represent the sum of the frequencies of mutations at each position (right y-axis). **c** Sequence Logo plots showing the frequencies of mutations from LSS in vitro evolution (color legend in upper right). **d** Sequence Logo plots showing the frequencies of mutations from HSS evolution. **e** Mutation frequencies of the receptor binding motif (RBM) representing sequences obtained from a spikenuc1207 file released on December 7, 2024 by GISAID. More detailed analysis including logos and mutation scatter plots for all libraries and Pango named lineages is provided in Supplementary Material Figs. S19–S36. Interactive versions for corresponding mutation scatter plots are available at https://host-patho-evo.github.io/mutation_scatter_plot/ enabling zooming and mouse-selection-based readouts of corresponding frequencies for given position/codon. **f** Comparison of mutation frequencies observed in natural viral populations (same as **e**) with those obtained from in vitro evolution experiments. Data shown for escape from neutralizing antibodies binding in convalescent serum are averaged values across all individuals from published dataset Greaney et al.^24^ (Fig. S38). Annotated and scalable figures can be found at https://host-patho-evo.github.io/mutation_scatter_plot/. Source data are provided in the file Source_Data_Fig-3.xlsx.

Mutation frequencies following LSS and HSS in vitro evolution for WT RBD are shown in Fig. 3a, b. Bars indicate the fraction of mutations per position (right axis), while circles represent the frequency of the specific mutation. Comparing LSS (Fig. 3a) and HSS (Fig. 3b) reveals that well documented Omicron mutations 440, 450, 452, 460, 477, 484, 498, and 501^32–37^occur at increased frequencies in both protocols. However, the evolution under both protocols is fundamentally different. HSS fixate specific mutations at very high frequencies, contrary, LSS mutation frequencies are much lower and the mutations are more variable. Logo plots of LSS and HSS libraries using non-WT starting variants are shown in Fig. 3c, d, respectively, demonstrating similar trends. Detailed mutation analyses are provided in Figs. S19–S23 for LSS and Table S4, Figs. S24–S35 for HSS and Fig. S36 for deposited viral sequences and lineages. Structure representation of the mutations accumulated at higher frequencies following LSS and HSS shows that they are clustered near the RBD-ACE2 interface (Fig. S37). For example, N460K is selected in both LSS and HSS, but at a frequency of ~1 in HSS vs. 0.14 in LSS. Similarly, S477N and T478K are enriched to frequencies of ~1 in HSS, and 0.2 and 0.002 in LSS. Overall, LSS results in a greater number of mutations with frequency >0.01 (204 mutations on average with 69 mutations being non-synonymous) than HSS (68/40 mutations) (Fig. 3a-d). Comparing mutation frequencies after two and four rounds of HSS shows that dominant mutations were already selected by round two, but their prevalence increased by round four (Table S4, Figs. S15–S35). A detailed comparison of high frequency mutations with SARS-CoV-2 evolution is shown in Table S5. The complete list of mutations and their respective frequencies was deposited at Zenodo.org under 10.5281/zenodo.19475241 accession and also as interactive mutation scatter plots at https://host-patho-evo.github.io/mutation_scatter_plot/.

Positions 498 and 501 are of particular interest as the double-mutation Q498R/N501Y emerged with Omicron, and is conserved since, while N501Y was established already from the Alpha lineage. Under HSS, the Q498R/N501Y double mutation was established in round 2 and did not further evolve. In contrast, LSS starting from WT led to N501Y in 30% and Q498R in 15% of clones after seven rounds, with Q498H appearing in 69% of the sequences. Notably, Q498H alone emerged rapidly under low-stringency selection in mammalian display experiments^38^ and is associated with adaptation to mice^35^. Furthermore, Q498H acquisition is often followed by N501T or N501S, suggesting epistasis, similar to Q498R/N501Y. When LSS started from Alpha or Beta lineages, where N501Y is present, it is maintained, with Q498R emerging in 35–96% of the sequences (Table S6). When both Q498R and N501Y were present at the onset of LSS (in BA.1 and RBD-v48), they remained fixed throughout selection (Fig. 3c and Table S6). In previous work, we demonstrated the synergistic effect of Q498R and N501Y^25^, which is strongly selected under HSS and maintained under LSS, representing an evolutionary tipping point directing RBD evolution.

We included the RBD-v48 variant for LSS evolution as an intriguing experiment to evaluate how its 50-fold increased binding affinity^25^ will contribute towards evolutionary plasticity. Indeed, this increased affinity provided a surplus of affinity providing broader mutational tolerance, allowing the fixation of unique mutations such as A348P, A363P, K386E, N388D, N460S, I468V, V483A, and S514T identified by Sanger sequencing (Fig. S11) and NGS (Fig. S22). Available deep mutational scanning data for the WT RBD indicate minimal impact of these mutations on binding (± 0.07 Δlog10(KD,app)^23^), likely explaining their tolerance.

Interestingly, the S375F mutation, characteristic of Omicron but not interacting with ACE2, was rapidly eliminated and reverted under LSS in vitro evolution conditions (see BA.1, Fig. 2f and S23a, c), suggesting a strong selective disadvantage in our in vitro evolution system. The display of RBD variants with and without this mutation revealed a striking difference, indicating its dramatic structural and functional impact (Fig. S2b). The continuous presence of S375F in all Omicron lineages is likely driven by factors other than adaptation to human receptor binding^39,40^. The K417N mutation (characteristic of the Beta and BA.1 variants) showed different behavior. It did not revert to the original residue and further diversified into several accepted substitutions, with K417I and K417Y emerging as most prominent (Figs. S21–S23).

HSS produced a distinct mutation profile (Fig. 3d) including N440K, S477N, T478K, Q498R, and N501Y when starting from WT and WT + I358F, already after two rounds of mutagenesis. This confirms their role in enhancing ACE2 binding while maintaining protein stability. These mutations are native in BA.1 and BA.2, and did not further evolve when they were used as starting points (Table S4). N460 evolved to K starting from both WT and Omicron. While this mutation is not observed in BA.1 or BA.2, it is fixed in late Omicron variants (Table S5), clearly pointing towards its importance. Position 484, which changed from E (WT) to K (Beta variant) and A (in BA.1 and BA.2), evolved to R or A under HSS in WT and WT + I358F, respectively, a pattern also seen in BA.1 and BA.2 (Tables S4 and S5). Position 450 evolved from N to D in WT, BA.1, and BA.2, while 452 (L in WT, R in delta) evolved to M or R under HSS in WT, WT + I358F, BA.1, and BA.2. Positions 446 and 496, G in WT and BA.2 but S in BA.1, reverted in BA.1 evolution to G. Position 505, R in WT and H in Omicron, remained H in BA.1 and BA.2, but starting from WT it initially mutated to H before reverting to Y. Overall, our results reveal a high degree of similarity between HSS-driven evolution of WT, BA.1, and BA.2 RBMs, resulting in Omicron like variants (Figs. 3d, S15–S18 and S24–S35). Interestingly, the SARS-CoV-2 Y453F mutation, previously observed in minks^36,37^ and known to strongly enhance ACE2 binding affinity^23^, was selected only under LSS. This suggests a potential incompatibility between Y453F and other affinity-enhancing mutations selected under HSS.

### High stringency selection for ACE2 binding recapitulates viral evolution

BA.1 and BA.2 were the first variants of Omicron that were identified in November 2021. These were followed by additional Omicron lineages, accompanied by a selective sweep that caused the previous lineages to disappear. To compare HSS, either starting from WT or Omicron sequences towards the overall variability of the RBD sequences as determined through virus sequencing from 2020-2024, we gathered the complete set of named Pango lineages released on September 12, 2024 (https://github.com/corneliusroemer/pango-sequences, Table S5 and S36B,D)^41^ and all GISAID sequences^42^, incorporating a set of spike nucleotide sequences as of December 7, 2024 release (spikenuc1207.fasta, 17,039,211 sequences, Fig. S36A,C) (Fig. 3e). While this analysis is biased towards countries with abundant sequencing, with the fraction of patients sequenced being sharply reduced over time, it provides a qualitative view of the evolution of the RBD of SARS-CoV-2. Table S5 shows a comparison of the overall frequencies of amino-acids calculated from GISAID (only frequencies >0.01 are shown) and those calculated from 2023 and onwards (termed advanced) in comparison to the same residues after 4 rounds of HSS starting from WT, WT + I358F, BA.1 and BA.2 sequences. The data show a clear similarity between HSS and natural evolution (see also Fig. 3f). From the 29 mutations with frequencies in SARS-CoV-2 > 0.01 in GISAID records, 24 were selected by HSS. Interestingly, those that were not selected had all relatively low frequencies in SARS-CoV-2, which some eventually increasing in the advanced Omicron lineages, maybe due to an advantage in immune evasion. Also vice versa, from the 33 amino-acid variations selected by HSS with frequency of >0.03 from at least one starting sequence, 25 were evolved also in SARS-CoV-2 with frequencies >0.01 in GISAID records.

Interestingly, K444T, N450D, L452M/R and E484R did evolve in HSS, but their occurrence among SARS-CoV-2 samples is highly lineage specific. K444T and N450D are observed in more advanced Omicron lineages, such as BA.2.75 and BQ.1 descendants (BR.4, CH.1, BN.3.1). L452M appeared in CM and BA.2.74. L452R is frequent in many lineages including descendants of BA.5. An outlier is E484R, which was fixed in HSS from three starting library sequences (except WT + I358F). The natural frequency of this mutation is relatively rare, appearing in B.1.351^43^, CM.x lineages and a few others. This suggests that E484R contributes to ACE2 binding (as is the case for E484K^23^), but is likely hindering viral fitness by different mechanisms, as supported by a pseudovirus assay^44^. One possibility may be that A in position 484 affects RBD stability, as it strongly evolved only in the starting library WT + I358F (Table S5), which is a more stable variant.

Additional mutations observed in more advanced Omicron variants, including F486P^45,46^, and the so-called Flip mutations L455F, F456L^47,48^ (and thus may be important for immune evasion), were selected by HSS starting from WT and WT + I358F sequences, yet not to high-frequencies. Additionally, V445H and N481K, which were detected across all HSS libraries, are characteristic of BA.2.86 and its descendant lineages^49,50^. This shows that in vitro evolution catches not only the highly advantageous mutations (which were rapidly fixed), but, at lower frequencies also mutations that evolved later in Omicron.

In contrast to the mutations discussed above, the mutations G446S, Q493R, G496S and Y505H exhibit distinct evolutionary trends. These residues are present in natural evolution, but did not evolve to high frequencies using LSS or HSS. Y505H did evolve in the second round of HSS, but its frequency was reduced in the 4th round (Table S4). Y505H interacts with the neighboring Spike chain residue P373 in closed conformations^51^, raising the possibility of evolutionary advantage for the closed conformation. Mutation G446S emerged in BA.1 and there persisted similarly to its persistence in both LSS and HSS BA.1 libraries. In other libraries, as also in BA.2 and its descendants, it evolved to low frequency (of 0.01-0.03, which is still 1000-fold above baseline). Thus, it seems that the replacement to S brings only a limited advantage over G. Similarly, Q493R appears to be context-dependent, persisting under certain conditions but being eliminated under others (Table S5). The G496S was eliminated from both natural and HSS evolution (Table S5). The transient presence of G446S, Q493R and G496S mutations, despite their eventual disappearance, supports the hypothesis that Omicron evolved in immunocompromised patient(s). In such individual(s), prolonged intra-host viral evolution was likely influenced by highly specific selective pressures (specific antibodies) leading to the selection of unique mutations, which were eliminated upon transmission to the general population^52–55^. Still, we cannot exclude the possibility of adaptive evolution in an animal reservoir to different ACE2 orthologs where their fitness is positive^3^.

Our experimental design excluded antibody selection. However, we were able to reuse complementary datasets from studies specifically focused on antibody escape, in which ACE2 binding was not a stringent constraint. This allowed us to deconvolute mutations primarily driven by receptor affinity from those enriched due to immune escape. Here, we utilized the comprehensive frequency data generated by Allison J. Greaney and colleagues (Prof. Jesse D. Bloom laboratory^24^) and compared their averaged values across all individuals (Fig. S38) with our experimental results and frequencies observed in circulating viral populations recorded in GISAID. The combined analyses are presented in Fig. 3f, with an annotated version available at [https://host-patho-evo.github.io/mutation_scatter_plot/]. The plot shows that the stronger selected mutations in HSS are also more frequent in SARS-CoV-2. In addition, this group of mutations also has some neutralizing potential, which may be due to them being within the ACE2 binding site (Fig. S37), which is highly enriched for neutralizing mutations. This comparison identified that L452M and E484R were predominantly enriched due to enhanced ACE2 binding. For Q498R and N501Y, both antibody escape and receptor-binding advantages act synergistically to promote their selection.

### HSS but not LSS selection for ACE2 binding results in tighter-binding clones

**Fig. 4:**
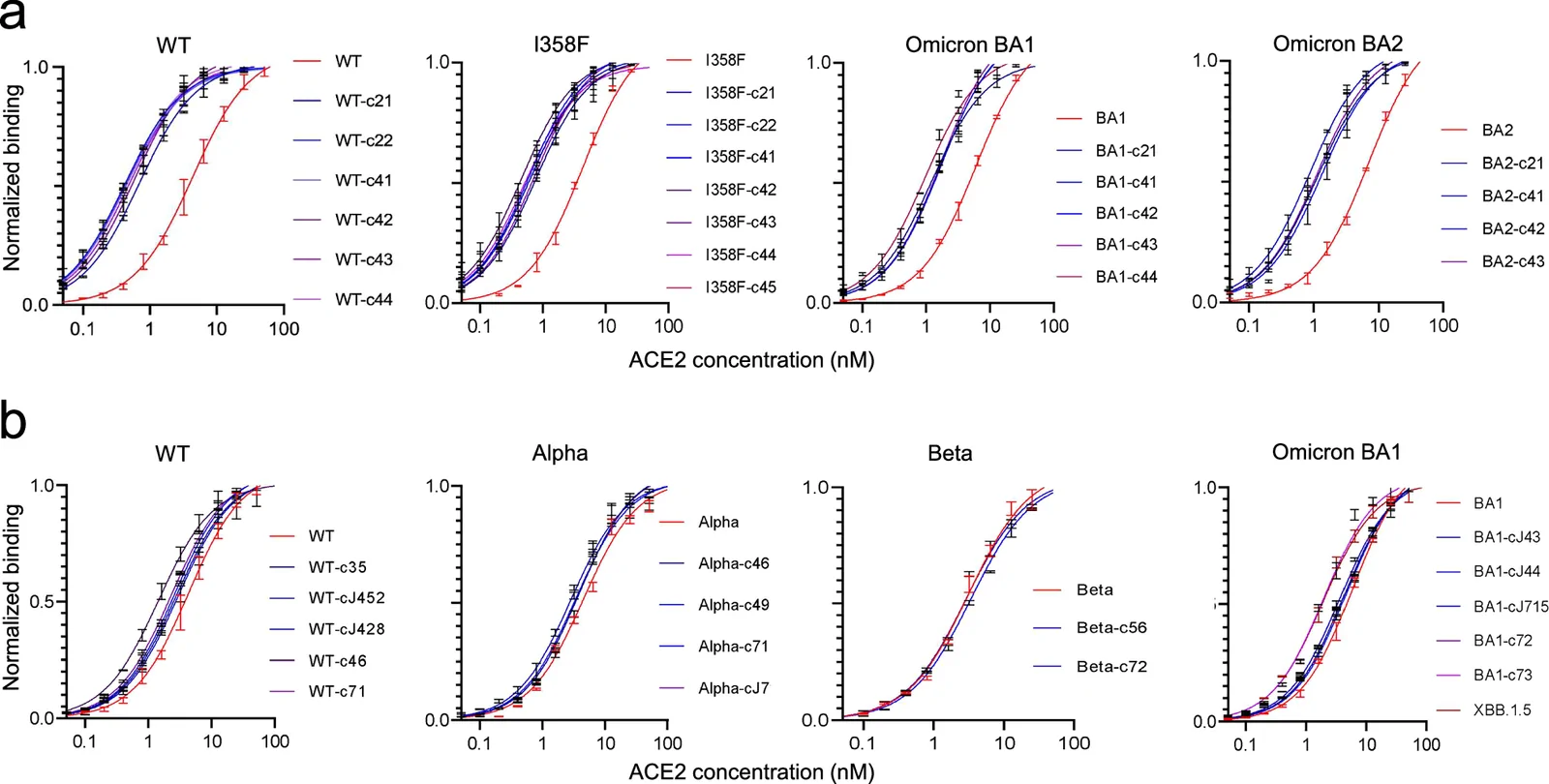
Binding affinities of selected clones. High-stringency selection (HSS) (**a**) and low- stringency selection (LSS) (**b**) clones were sequenced and their binding affinities were determined by titration curves of increasing concentrations of fluorescently labelled angiotensin converting enzyme 2 (ACE2) peptidase domain, bound to receptor binding domain (RBD) proteins displayed on the surface of yeast. The red line represents the parental variant. *K*_D_ values and sequences for the different clones are given in Table S7. Individual clones are labeled with the following code: parental_RBD, library number (number of random mutagenesis libraries, following rounds of selection), clone_number e.g. Alpha-c71. Data are presented as mean values ± SD from *n* = 3 independent biological replicates. For details and number of biological repetitions for the individual measurements see source data for Fig. 4. Source data are provided in the file Source_Data_Fig-4.xlsx.

To evaluate and confirm the driving force behind the in vitro selections, we measured binding affinities and RBD thermostability for different clones following HSS and LSS evolution (Fig. 4 and Table S7). An intriguing outcome is that in all cases, HSS selection resulted in increased binding affinity by about 10-fold, reaching 0.4–1.5 nM, independent of the evolved variant (Fig. 4a). This is in line with the increased binding affinity values of many of the SARS-CoV-2 lineages (Fig. 4 and Table S7). The thermostability of the HSS evolved variants was ~55 °C, including for BA.1 and BA.2, where the starting clones were less stable by 8 and 5 °C, respectively (Figs. S2c and S39). Interestingly, increased binding affinity was observed already following the 2^nd^ round of in vitro evolution, and did not further improve, in line with the sequence selection. These results clearly highlight the strong selective pressure favoring a subset of mutations creating high-affinity variants with sufficient thermostability and increased binding affinities. Binding affinities of LSS derived clones were in the range of 2-5 nM, comparable to their parental lineage (Fig. 4b and Table S7). Different from HSS evolution, in LSS no consistent increase in thermostability was observed (Fig. S39).

### Simulations of evolutionary trajectories

**Fig. 5:**
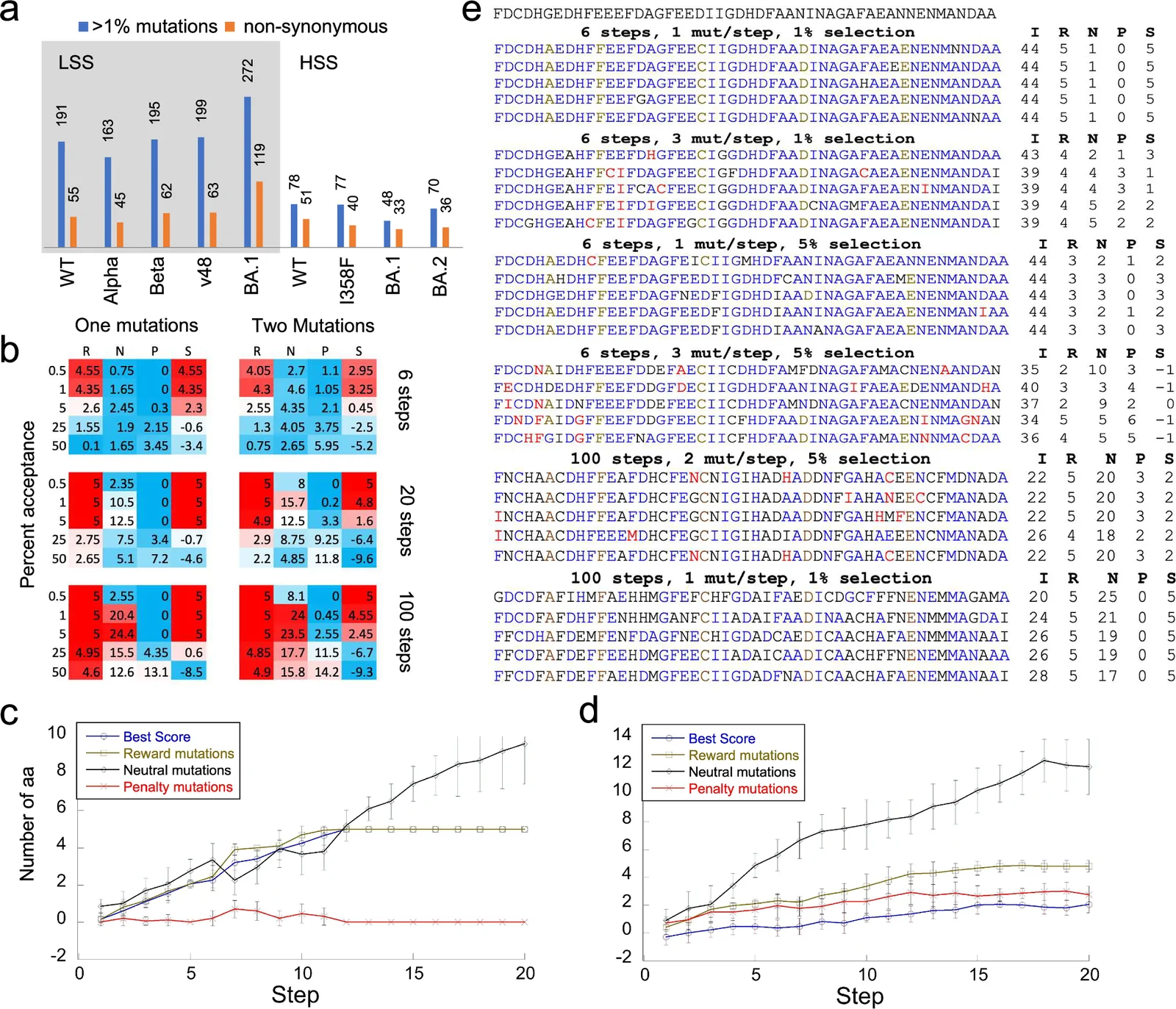
Simulating the acquisition of mutations throughout evolution. **a** The number of mutant codons (synonymous + non-synonymous) with frequencies over 0.01 in the given library in comparison to non-synonymous mutant codons following 7 and 4 rounds of low- stringency selection (LSS) and high- stringency selection (HSS). **b**–**e** A 50-letter string composed of 10 different characters is subjected to random mutagenesis, with the top scoring sequences (0.5-50%) progressing to the next round of mutagenesis and selection. Specific positions (6, 11, 21, 31, and 41) are designated as reward [R] positions, yielding a positive score when mutated to A, F, C, D, and E, respectively. Each position permits two neutral [N] letters, including the initiating letter, while the remaining 8 (or 7 at reward positions) were assigned a negative score, penalty [P]. **b** Quantification of reward, neutral, and penalty mutations, as well as final scores (S), after applying different selection stringencies (top 0.5%, 1%, 5%, 25%, and 50% of scoring sequences). Results for single mutations per step (left) and double mutations per step (right) are shown. Heatmaps (red to blue) depict the average score from 20 independent simulations. **c** Simulation progression over 20 rounds with single mutations (2000 per round) and the top 5% of scoring sequences transferred to the next round. A rapid rise in reward mutations is observed, plateauing after approximately 10 rounds, achieving the maximum score of 5. This is accompanied by an increase in neutral mutations at each round. Data are presented as mean values ± SD from *n* = 20 independent simulations. **d** Simulation progression with double mutations (2000 per step), showing slower score improvements, reduced incorporation of reward mutations, and increased accumulation of neutral and penalty mutations. Data are presented as mean values ± SD from *n* = 20 independent simulations. **e** Final sequences obtained after 6 and 100 rounds, using selection thresholds of the top 1% or 5%. Colors are as in (**c** and **d**). Source data are provided in the file Source_Data_Fig-5.xlsx.

The robust convergence of mutations underscores how stringent selection conditions shape mutational landscapes, driving adaptation toward highly optimized receptor binding domains while limiting the diversity seen in low-stringency selection. This conclusion is further supported by the markedly lower number of synonymous mutations after HSS in comparison to LSS (Fig. 5a), which was also observed in the BA.1 and BA.2 lineages. To further support this observation, we investigated evolutionary trajectories using simulations based on a number of rules as outlined below:

1. As an input sequence we used a string of 50 letters, each drawn from a set of 10 different characters (arbitrary, not amino-acids). Mutations at positions 6, 11, 21, 31, and 41 to the letters A, F, C, D, and E respectively, were given a reward. The simulations started with a neutral letter at all positions, with one additional neutral letter and 8 penalty letters available (or 7 at reward positions).
2. Selection criteria: At each step, the top 0.5%, 1%, 5%, 25%, and 50% of scoring values were selected for the next round of mutagenesis and selection.
3. Simulation setup: 2000 single or double mutations were randomly introduced per step, with varying selection stringency.

When single mutations were introduced with stringent acceptance criteria (0.5% or 1%), reward residues were rapidly established (already after 6 steps), with minimal accumulation of neutral or penalty mutations. They accumulated only after acquisition of all reward mutations. By contrast, introducing two mutations in parallel led to lower scores and greater accumulation of neutral and penalty mutations even under stringent selection (Fig. 5b). Increasing the number of steps in the simulations (20 or 100 steps) increased the number of neutral mutations at all positions, with penalty mutations appearing more frequently at lower selection stringency (Fig. 5b, e). The results of the simulations explain the rapid accumulation of Omicron mutations, which enhance binding affinity, within only two rounds of yeast display selection. Furthermore, the simulations explain the minimal number of bystander mutations observed in early rounds of HSS yeast display and in natural viral evolution.

### SARS-CoV-1 affinity maturation reveals ACE2 adaptation paths

Knowing that the characteristic mutations of Omicron lineages evolved under HSS, we can utilize this insight beyond SARS-CoV-2. We consider that SARS-CoV-1 adapting to the human population would follow an initial evolutionary phase characterized by increasing receptor-binding affinity, as observed in SARS-CoV-2. The SARS-CoV-1 emerged in 2002–2004, infected over 8000 individuals across 30 countries and territories and resulted in nearly 1000 fatalities (https://www.emro.who.int/health-topics/severe-acute-respiratory-syndrome/)^56^. Although this outbreak was successfully contained, closely related viruses continue to circulate in populations of Chinese horseshoe bats and masked palm civets^57,58^. The limited human-to-human transmission of SARS-CoV-1 has been attributed to its reduced binding affinity for the human ACE2 receptor^23^ and the restricted size of the infected population, which constrained the emergence and competition of adaptive mutations. During the SARS-CoV-1 outbreak, emerging mutations were closely monitored and analyzed^59–62^. However, the identified receptor-binding domain (RBD) mutations mostly reduced ACE2 affinity. This is in line with previous observations that a low number of individuals in the population under permissive conditions allows significant variation due to chance and the fixation of alleles even with a low fitness score^63,64^.

**Fig. 6:**
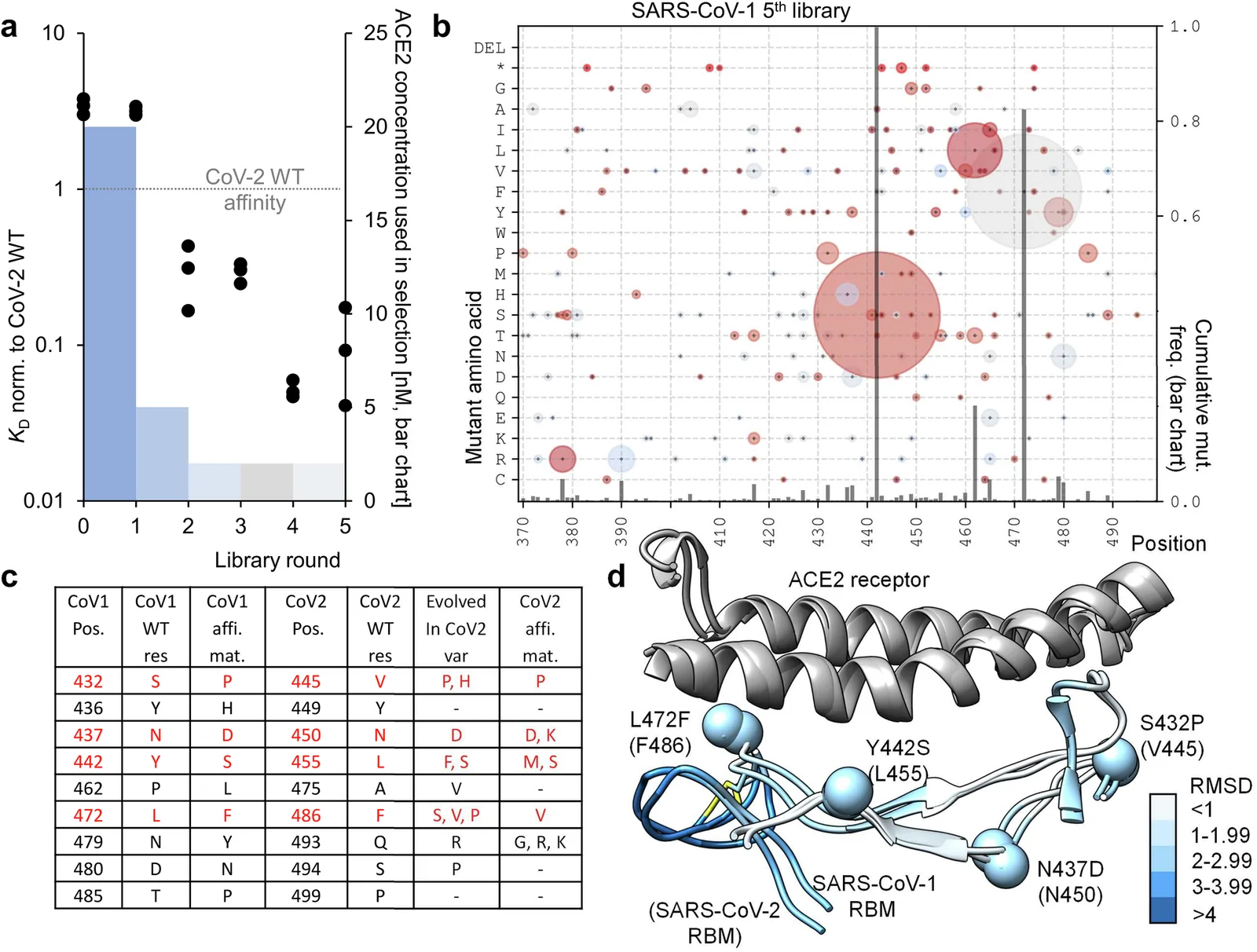
SARS-CoV-1 selection for high affinity binding of human ACE2. **a** Progression of affinity maturation across individual libraries (1–5) (right axis, bar chart). Black points (left axis) represent *K*_D_ values normalized to SARS-CoV-2 affinity for SARS-CoV-1 (library round 0, triplicate) and three randomly selected clones after completed selection library step (dots at the right edge of each bar). The sequences of these clones are shown in Fig. S40. **b** Mutation scatter plot showing the distribution of mutations in the 5th library above 0.1% threshold. The legend matches that of the mutation scatter plot in Fig. 3, where the radius of the circle represents mutation frequency in the population, and the color indicates the evolutionary distance from the original amino acid. **c** Selected residues during affinity maturation of SARS-CoV-1 and SARS-CoV-2, along with an analysis of the structural similarity of their receptor-binding motifs (RBMs) in binding to angiotensin converting enzyme 2 (ACE2) peptidase domain. Residues showing evidence of convergence are highlighted in red and are depicted as spheres in the structure comparison. **d** based on structures ACE2 and SARS-CoV-1 (PDB: 6CS2^65^) and ACE2 and SARS-CoV-2 (PDB: 6m17^66^). Amino acid residue number of SARS-CoV-1 with original and mutated AA residue is enlisted first and followed by residue number from SARS-CoV-2 (in braces with just the original AA residue shown, visualized in UCSF Chimera 1.18). Source data are provided in the file Source_Data_Fig-6.xlsx.

To address the need for systematic identification and characterization of mutations with the potential to enhance receptor-binding affinity, we performed affinity maturation of SARS-CoV-1 RBD applying three rounds of HSS followed by two additional rounds of LSS to expand the interface’s mutational variability (Figs. 6a, S40). After three rounds of HSS, three mutations, Y442S, L472F, and P462L were fixed in the population with frequencies 0.99, 0.82 and 0.18, respectively. Remarkably, Y442S alone was sufficient to close the binding affinity gap between SARS-CoV-1 and SARS-CoV-2. Subsequent low-stringency evolution identified six additional beneficial mutations, further enriching the mutational landscape of the SARS-CoV-1 RBD (Fig. 6b, c). Notably, four of the identified mutations exhibited convergent evolutionary patterns between SARS-CoV-1 and SARS-CoV-2. Mutation N437D is the only mutation identified in SARS-CoV-1 virus - ShanghaiLY strain (Uniprot: P59594). These mutations were localized to regions with low root-mean-square deviation between SARS-CoV-1 (PDB: 6CS2:B^65^) and SARS-CoV-2 (PDB: 6m17:F^66^), indicating similar binding modes (Fig. 6c, d).

## Discussion

In vitro evolution of proteins is a technique that selects for the amino-acid sequences that provide for a given trait. Here, we applied this technique to follow the sequence evolution of the RBD of the spike protein of SARS-CoV-2 upon binding to the human receptor ACE2, which is the first step in viral infection. As many of the hallmark mutations along the evolution of SARS-CoV-2 are located in the RBD (and more specifically in the RBM) we wondered whether in vitro evolution of the RBD-ACE2 binding would mimic viral evolution during the course of pandemic, and whether we can use it to understand the environmental pressure driving it. For this, two regimes of evolution were employed: low and high stringency. In the LSS regime, the bait protein (ACE2) concentration was in the range of WT affinity (2 nM), with 3-10% of the clones being selected for the next round. In the stringent regime, the concentration of ACE2 was gradually reduced to 0.15 nM, with 1% of the best binding clones being selected. Moreover, in the stringent regime, a heating step to 40 °C for 20 min was added, to eliminate mutations causing marginally stable prey proteins. As a result, LSS selects for a variety of sequences resulting in good binding, while HSS rapidly selects for better binding and stable proteins.

In LSS, random mutagenesis was applied to the complete RBD starting from 5 different sequences representing different SARS-CoV-2 variants (WT, Alpha, Beta, RBD v48, BA.1; Fig. 1b). HSS was done from 4 different starting sequences (WT, WT + I358F, BA.1, and BA.2; Fig. 1b) mutating only the RBM and not the stability-determining motif AA 337 - 430. Random mutagenesis, with equal distribution of 1-3 mutations per clone (detailed distribution in Fig. S7), was verified on the starting libraries for both LSS and HSS, showing that two adjacent nucleotide mutations are rare (in line with virus evolution^8^ - Fig. 2c). Consecutive steps of random mutagenesis and selection were employed to reach substantial divergence from the starting sequences. This was achieved after 7 rounds of mutagenesis for LSS and 4 rounds for HSS. HSS rapidly converged, achieving a high frequency of mutations characteristic for Omicron already after 2 rounds of mutations (Table S4), with only few background mutations, while LSS did not converge. Detailed analysis using Sanger sequencing and NGS sequencing following 2, 3 and 4 rounds of HSS showed very little differences in the evolved mutations, with even higher frequencies of accumulation of critical mutations in individual clones (Figs. S15-S18 and S24–S35).

Comparing LSS to HSS shows that in both cases the mutations accumulated in the RBM-ACE2 interface (Fig. S37). However, the pattern of accepted mutations differs, with the absolute number of mutations following LSS being higher, however most at low frequencies, with a higher number of synonymous mutations. Conversely following HSS fewer mutations were recorded; however, selected mutations were at much higher frequencies (see also Figs. 5a and S7-S35). Moreover, a much higher fraction of mutations was non-synonymous after HSS than after LSS, showing that HSS is more target oriented (Fig. 5a). This is in line with mutation patterns of the virus, where most mutations of the S-protein in BA.1 and BA.2 were non-synonymous^67^ and the mutated residues were of high frequencies (Fig. 3e). Simulating the conditions of high and low stringency mutations (Fig. 5) shows that HSS leads to fast accumulation of rewarding mutations, while non-rewarding mutations are scarcely selected during the first few rounds of in silico evolution. Conversely, simulating LSS conditions results in slower accumulation of reward mutations and higher accumulation of neutral mutations (Fig. 5b-e). The agreement between mutation accumulation in the RBD between the virus, in vitro evolution and in silico evolution points towards the mechanism driving mutation accumulation in the RBD and supports the substantial role of receptor adaptation in shaping SARS-CoV-2 evolution.

Notably, key mutations such as N440K, L452R, N460K, S477N, T478K, Q498R, and N501Y were enriched under HSS, mirroring their individual convergent evolution in multiple VOCs and combined emergence in Omicron and its descendant SARS-CoV-2 lineages. Additionally, we observed the selection of mutations associated with more advanced Omicron variants, including K444T/R, N450D, F486P and Flip 455–456, as well as some mutations characteristic of BA.2.86 (Table S5 and Fig. S36). The presence of these mutations in both in vitro and real-world viral evolution suggests that receptor-binding constraints played a crucial role in SARS-CoV-2 adaptation. Conversely, certain binding interface mutations, such as G446S, Q493R and G496S, were rapidly eliminated during in vitro evolution, suggesting structural or functional disadvantage. This supports the hypothesis that specific immune-driven selection in immunocompromised individuals, rather than receptor adaptation, was responsible for the fixation of some Omicron mutations and explains their eventual disappearance in descendant lineages. Interestingly, the non-RBM mutation S375F that destabilizes the RBD and reduces its binding affinity to ACE2 and yeast surface expression (Fig. S2) was quickly eliminated in our experiment (Figs. S2 and S12), yet it persists in the virus evolution, suggesting an overall fitness advantage. It should be noted that the use of the isolated RBD domain for in vitro evolution has limitations, as it may lack stabilizing contacts present in the full Spike trimer that can compensate for the destabilizing effect of the S375F substitution. Structural studies have shown that S375F favors the RBD adopting the down conformation^68–70^, where substantially more intermolecular contacts are formed. Nevertheless, the overall stability of the Omicron Spike, at least in the initial inflection region, remains markedly reduced^71^. Sanger sequencing of individual LSS rounds revealed minimal fixation of mutations outside the RBM region (amino acids 336–430). In later rounds (≥3), partial fixation of the stabilizing mutations A348P and I358F was observed (with I358F being used also as a starting mutation in HSS)^25,72^. Overall, these results suggest that this portion of the RBD without destabilizing Omicron mutations has a low impact on the acquisition of mutations within the RBM. Therefore, the presence of the S375F substitution, which is largely responsible for the Omicron phenotype^39^, must be supported by complex epistatic effects underlying immune escape or tissue tropism switching. These effects likely confer a positive fitness advantage but cannot be directly recapitulated in our experimental system. It should also be noted that despite S375F being fixed in HSS (where only the RBM was mutated), its thermostability reverted rapidly to WT values, due to secondary mutations (Fig. S39).

Mutations primarily associated with immune evasion, particularly in the 444–456 and 486–490 regions, were not significantly enriched in our experiments, reinforcing the idea that immune pressure, rather than receptor adaptation, drove their selection in vivo^73–75^. Yet, these mutations were significantly represented in the analyzed libraries (frequency >0.001). Unlike most other variants, these were not excluded entirely, suggesting that they are tolerated and do not exert a major negative effect on binding or stability. Importantly, our experiments were intentionally designed to exclude antibodies from the selection process, allowing us to examine RBM evolution in the absence of immune-related selective pressures. This approach provides a clear view of how receptor binding alone shapes SARS-CoV-2 RBD evolution, independent of antibody-driven selection. Although the fixation of major HSS mutations did not require immune system pressure, these mutations consequently contributed to immune escape by altering the RBD protein binding interface physicochemical properties. Our analysis using the BLOSUM62 score matrix which is considered as a universal quantitative approach defining whether an amino acid substitution is conservative or nonconservative (color coding, Figs. 3a, b, S19-S36) shows that properties of most fixed HSS mutations differ significantly from the original amino acid, including frequent mutations altering surface charges. One of the likely mechanistic explanations for these changes is an improved charge complementarity of the interaction^25^. Comparing our results with an existing dataset mapping how mutations affect recognition by polyclonal human plasma antibodies^24^ showed that residues selected under HSS most closely matched the mutation frequencies observed in GISAID (Fig. 3f). A subset of selected mutations exhibited dual characteristics with enhancing both receptor binding and immune escape (e.g., N460K, S477N, Q498R, N501Y). In contrast, other positions showed that residues conferring stronger immune escape, such as E484F, were not selected. This suggests that, at these sites, selection favored residues tolerated in terms of receptor binding yet providing a partial immune escape advantage, as exemplified by E484A. This finding is particularly important for applying similar methodologies to predict advanced viral lineages and mutations in scenarios where a large portion of the human population is no longer immunologically naïve. It is evident that immune escape mutations will preferentially emerge from variants that balance receptor binding affinity and immune evasion which are represented in our experiments as the low frequency mutations.

An interesting question that can be answered by our experiments is whether Omicron emergence, which is clearly the SARS-CoV-2 variant with human adapted RBM, was inevitable. We show that it emerged rapidly under HSS, but did not emerge under LSS even after 7 rounds of selection where most clones diverged from the Omicron residues with Y453F, Q498H, and N501T being the most frequent mutations. However, using BA.1 as the starting library, Omicron was conserved also under LSS. This shows that HSS was crucial for the evolution of Omicron but not its maintenance, which is driven by its evolutionary advantage. Omicron did not emerge through stepwise evolution in the general public, but through a drastic shift in the mutation landscape as an example of saltatory evolution^76^ which was, as we demonstrate in this manuscript driven by HSS likely within a small closed environment (maybe in immunocompromised patients). Our data indicate that rapid and dramatic accumulation of context-specific beneficial mutations can occur solely under strong selective pressure for the relevant trait, without the need for any additional mechanism that would increase the intrinsic mutation rate. Within the general public, LSS together with immune evasion were the dominant evolutionary drivers for a long time, until Omicron emerged as is supported by convergence of LSS and specific mutations in pre-omicron evolution^8,77,78^. Could another drastic event change the mutation landscape again? At least from the stability of Omicron mutations under our HSS and LSS conditions the answer is no. However, a drastic shift in evolutionary pressure could change this prediction.

The repeated and stable selection of Omicron-like RBD sequences under stringent conditions despite different starting conditions supports the feasibility of this approach for studying viral adaptation. Given that Omicron and its sub-variants have dominated SARS-CoV-2 evolution since late 2021, we postulate that Omicron represents a “humanized” form of SARS-CoV-2 RBM, optimized for human ACE2 binding and its key mutations will stay, while the immune escape mutations will continue to evolve at different positions. The ability to recapitulate its emergence and deconvolute the individual selective pressures responsible for mutation acquisition through directed evolution highlights the predictive power of our approach and its potential for anticipating evolutionary constraints in emerging viruses.

Our findings also suggest that similar in vitro evolution approaches can be applied to predict adaptive landscapes of other viruses. The in vitro evolution of the SARS-CoV-1 RBD, guided by our observations, identified residues that could potentially become fixed during the adaptation of this virus, or a closely related one, to the human ACE2 receptor. Strikingly, closing the affinity gap between SARS-CoV-1 and SARS-CoV-2 for ACE2 binding, which has been linked to the lower infectivity of SARS-CoV-1^23^, requires only a single substitution, Y442S. In addition, amino acid residues that share the same binding mode in both SARS-CoV-1 and SARS-CoV-2 exhibit a notable degree of convergence in our selection experiments. This similarity suggests potential cross-reactivity of immune responses targeting these sites, thereby constraining the possible adaptive trajectories. Overall, this experimental evolution framework provides a powerful strategy for identifying mutations that could enable a virus to cross the species barrier and efficiently infect humans. It also highlights mutations whose careful surveillance should be prioritized. Comparable in vitro evolutionary analyses performed under different selective pressures could help dissect the subsequent evolutionary processes that drive immune escape, or shifts in tissue tropism toward the upper respiratory tract^15,79^, features characteristic of later epidemic stages, and ultimately advance our understanding toward true predictability of viral evolution. Ultimately, the lessons from both the 2002–2004 SARS outbreak and the COVID-19 pandemic highlights the critical importance of early detection, transparency, data sharing, and international collaboration in preventing and mitigating future pandemics.

## Methods

### Cloning and DNA manipulations

For the modification of yeast display vectors, *S. cerevisiae* optimized genes for *SpyCatcher003* and *DeMA* monomeric avidin (Supporting information part PS1) were purchased from Twist Bioscience, and subsequently plasmids pJYDC1 and pJYDC3 (Addgene IDs 162458 and 162460) were modified by restriction free cloning^80^ similarly to previous modifications^27^. Barcodes (Table S1) were incorporated into amplification primers and introduced into pJYDC1-3EFR-Cfr-anti-StreptavidinAPC plasmid by homologous recombination^27^. Non-optimized *RBD* genes (amino acids 330–529, Table S2, Twist Bioscience) and the previously mentioned genes were PCR-amplified using KAPA HiFi polymerase (Roche) with specific primers forward: GACAAGGTACCGGAAGTACAAGTGCTAGCCATATGGGTTGCCCTTTTGGTGAAGTTTTTAACGC and reverse: gcttttgttcGGTACTGCCATTTCCGGATCTGGATCCTTTAGGTCCACAAACAGTTGCTGGTG. Amplicons were purified using the NucleoSpin Gel and PCR Clean-up Kit (Macherey-Nagel) and eluted in double-distilled water. Site-directed mutagenesis to introduce the I358F mutation was performed on the *WT RBD* using restriction-free cloning by PCR and primer I358F (CCAGATTTGCATCTGTTTATGCTTGGAACAGGAAGAGATTTAGCAACTGTGTTGCTGATTATTCTG), similarly to incorporation of genes^80^. Parental plasmids were eliminated by *Dpn*I (1 h) mediated digestion, and the crude reaction mixture (0.8 µl) was directly electroporated (MicroPulser Electroporator, BioRad) into electrocompetent *E. coli* Cloni 10 G cells (Lucigen). Clones were screened by colony PCR and verified by Sanger sequencing and primers pCTCON_seq_FW: GCAGCCCCATAAACACACAGTAT and pCT_seq_R: CATGGGAAAACATGTTGTTTACGGAG. Four RBD variants (*WT*, *WT* + *I358F*, *BA.1*, and *BA.2*) genes were analogously cloned to mammalian expression vector pHLsec-1^13^, verified by sequencing and produced at large quantities with high purity for transfection by using Macherey-Nagel NucleoBond Xtra Maxi kit (Macherey-Nagel).

### DNA libraries preparation

Random mutagenesis of the C-terminal domain for HSS (*CTD*; amino acids 430–528, corresponding to RBM) was performed using the GeneMorph II Random Mutagenesis Kit. PCR conditions were optimized (template amount, cycle number) to yield 1 to 3 nucleotide mutations per *CTD*. Resulting CTD amplicons were cloned into pJYDC vectors via RF cloning, and mutation rates were validated by Sanger sequencing. Optimal mutagenesis was achieved using 10 ng template DNA and 30 PCR cycles. In parallel, non-mutated N-terminal domains (*NTD*; amino acids 330–429) were amplified using KAPA HiFi polymerase (Roche). Yeast plasmids were linearized with *Nde*I-HF and *BamH*I-HF restriction enzyme cleavage; all three DNA fragments (CTD, NTD, plasmids) were gel-purified, isolated by NucleoSpin Gel and PCR Clean-up Kit (Macherey-Nagel) and assembled by yeast mediated homologous recombination^81^. Random mutagenesis for LSS libraries was done by previously published Taq polymerase-based error prone mutagenesis procedure over the whole *RBD* gene with 1 to 3 nucleotide mutations per gene^25^.

### Yeast libraries preparation

Yeast libraries were prepared by the procedure analogous to published protocol by Benatui et al.^81^. *Saccharomyces cerevisiae* EBY100 cells (ATCC MYA-4941™) were grown in 400 ml YPD to OD_600_ ∼ 1.6 (~3 × 10⁷ cells/ml). Following two consecutive washes with ice-cold HPLC-grade water, the cells were washed once with electroporation buffer (1 M sorbitol, 1 mM CaCl₂) and conditioned in conditioning buffer (0.1 M LiAc, 10 mM DTT) for 30 min at 30 °C. Following conditioning, cells were washed twice with electroporation buffer, resuspended in 4 ml of electroporation buffer, divided to 1 ml aliquots and incubated with DNA for 5 min on ice. For HSS each electroporation mixture contained 6 µg of mutated CTD fragment, 6 µg of NTD, and 4 µg of linearized plasmid (three component assembly, Fig. 1b). For LSS, the mixture contained 12 µg of mutated *RBD* gene and 4 µg of linearized plasmid. Electroporation was performed using a BioRad Micropulser (2.5 kV, 5 ms) in 2 mm cuvettes. Cells were recovered for 2 h in 1:1 YPD media + 1 M sorbitol, then transferred to SDCAA selective media (–Trp) and grown overnight at 30 °C, 225 rpm. Diluted aliquots (10⁶ cells) were plated on SD-W agar to estimate transformation efficiency (more than 1.5 × 10⁷ clones per library was accepted).

### Yeast cultivation and expression procedures

Aliquots of yeast libraries grown in SDCAA media overnight were collected by centrifugation (4000 g, 3 minutes, 4 °C), resuspended in 1/9 expression media (1:9 glucose: galactose ratio^27^), and expression cultures were grown overnight at 20 °C (225 rpm). For subsequent experiments, yeast expressed cells were collected by centrifugation and washed with ice cold PBSB (PBS + 1 g/L BSA).

### ACE2 labeling and quantification of expression

Yeast samples with surface expressed proteins were resuspended in ice cold PBSB. Quantification of yeast surface expression levels was achieved by co-cultivation or post-cultivation incubation (1 hr) with one of the following substances, depending on used plasmid: 10 nM DMSO solubilized bilirubin for pJYDC1 (eUnaG2 reporter holo-form formation, green/yellow fluorescence, excitation 498 nm, emission 527 nm), 5 nM ALFA-tagged mNeonGreen for pJYDC3 (DnbALFA reporter nanobody), 50 nM SPY-tagged mNeonGreen for pJYDC4 (SpyCatcher003 reporter), or 5 nM biotin(Avi)-tagged mNeonGreen for pJYDC6 (DeMA reporter). ACE2 protein was labeled with CF-640R succinimidyl ester (Biotium, molar ratio 1:5; 1 hr at RT), and unreacted dye was subsequently quenched with 1 mM Tris. Protein concentration was determined via A280 (NanoDrop, Thermo Scientific; ε = 152,000 M⁻¹cm⁻¹, MW = 69.3 kDa) and confirmed using the BCA assay. Labeled ACE2 at working concentration (Fig. 1c) was added to yeast samples in volumes ensuring ligand excess and incubated overnight at 4 °C on rotator (45 rpm). After incubation, cells were washed twice in ice cold PBSB and filtered through nylon mesh prior to FACS.

### Library sorting

Expression and binding labeled HSS yeast libraries were heat-treated (40 °C, 20 min) prior sorting. eUnaG2 and mNeonGreen signal spills were compensated using ProSort software. The gating strategy is shown in Fig. S1. Sorting proceeded stepwise: a minimum of 20,000 cells were sorted, expanded for 48 h in SDCAA, induced, and re-sorted after new expression and ACE2 labeling to enrich the population. Round two usually enabled visual resolution of enriched vs. parental populations on dot plots. For HSS an additional round of sorting was applied for every library (3 consecutive sorts vs. 2 for LSS). DNAs from sorted populations were extracted using Lyticase method, and corresponding regions (either full RBD or CTD fragments) were re-amplified with in situ barcode specific primers to avoid contaminations (Table S1) for further rounds of mutagenesis and FACS. Aliquot of yeast extracted plasmids were electroporated into *E. coli* Cloni 10 G cells (Lucigen) and corresponding *E. coli* mini-preps (Wizard® Plus SV Minipreps DNA Purification Systems, Promega) from selected colonies were sequences with universal primers for pJYDC vectors^27^.

### Binding assays and affinity constant (*K*_D_) determination

Affinity constant values of selected RBD clones for different libraries were determined using the cytometry titration curve method. Small samples of yeast single clones (10ul from expression cultures, OD600 = 1.5, ~150,0000 cells) were co-cultivated with fixed concentration of expression labeling substrate (bilirubin 10 nM or ALFA-tagged mNeonGreen 5 nM, plasmids pJYDC4 and pJYDC6 were not used for *K*_D_ determination), and with increasing concentrations of labeled ACE2. A total of 12 concentrations were analyzed, in the concentration range starting from 50 nM to 10 pM. In order to verify that ACE-2 ligand is in excess compared to RBD molecules, large incubation volumes were used (especially at low ACE2 concentrations) to prevent ligand depletion. After incubation (overnight, 4 °C, mild shaking) yeast cells were washed twice with PBSB with increased concentration of BSA to 3 g/L to reduce the non-specific interactions. Cells fluorescence parameters were recorded by using Beckman Coulter Life Sciences CytoFLEX benchtop flow cytometer in plate format for automated acquisition. Minimum of 30 k cells in single cell population were recorded (Fig. S1). Results were analyzed using the FlowJo program. Gating was applied to normalize the expression rates across different measurements and to separate the positive population (expressing cells) from the negative population (non-expressing cells). To calculate specific binding at each single measurement, median APC-A channel intensity of the negative population was subtracted from the positive median value of the defined expression level population. The analysis done with defined expression level across measured clones increased accuracy of affinity determination for highly expression variable clones (Fig. S41). Specific binding values were plotted over corresponding ACE-2 concentrations (log Scale) and fitted to one-site specific binding equation using GraphPad v10, and best fit *K*_D_ values were generated from at least 3 replicate binding curves.

### Yeast surface displayed RBD stability assay

The protein stability of surface displayed RBD variants was analyzed by heating expressed yeast cells with a gradient temperature (from 37 °C to 73 °C) for 14 minutes in gradient PCR cycler prior to incubation with CF-640 labeled ACE-2 (6.4 nM, 2 ml volume). Subsequently cells were washed twice with PBSB with high concentration of BSA 6 g/L. Yeasts fluorescent parameters were analyzed by using Beckman Coulter Life Sciences CytoFLEX benchtop flow cytometer, and normalized APC-A intensity (specific binding fraction) was plotted over increasing temperatures. A Sigmoidal (logistic) curve was generated using GraphPad v10 (4PL, Four-Parameter Logistic model) and IC50 (melting temperatures) were calculated from at least 3 replicate binding curves.

### Biosafety statement

The work described in this manuscript provides a safe alternative to experiments with live viruses, as it involves only protein-based assays in a yeast display system. All yeast display experiments were conducted at Biosafety Level 2 (BSL-2) in accordance with protocols approved by the Centre for Experimental Biomodels, First Faculty of Medicine, the institution formally authorized to provide biosafety oversight. All personnel handling yeast were trained in relevant safety procedures and protocol-specific techniques.

### Recombinant RBD and ACE2 production and purification

For recombinant RBD protein production suspension HEK293F cells (Cytion cat. no. 300260) were grown and transfected using PEI-MAX transfection reagent according to the manufacturer’s protocol. Briefly, each RBD variant was produced in 200 ml of cell suspension transfected with 200 ug of plasmid DNA and 600 ul of PEI reagent. The media were collected after 80 hr of incubation by centrifugation (500 g, 5 minutes) and filtered using a 0.45 µm vacuum filter unit. Secreted protein bearing C-terminal his tag was purified on Ni-NTA agarose beads (HisPur Ni-NTA Agarose Resin, ThermoFisher) and BioRad gravity flow column (Econo-Pac® Chromatography Columns, 14 mL volume), supernatant was loaded, washed with 10 CV of PBS and eluted using PBS + 300 mM Imidazole (protein purity was analysed by SDS-PAGE, see Fig. S2). The Elution buffer containing imidazole was replaced by 100% PBS using dialysis buffer exchange. The ACE2 protein (ACE2D, carrying mutations outside the receptor-binding interface) was expressed in *E.coli* BL21(DE3) using the plasmid pET28-bdSUMO-ACE2D, following our previously established protocol^50^. Cells were cultured in 2×YT medium at 37 °C until reaching an optical density at 600 nm (OD₆₀₀) of 0.6. Protein expression was induced with 0.5 mM IPTG, and cultures were incubated overnight at 20 °C. Cells were harvested by centrifugation (8000 × g, 5 min), resuspended in lysis buffer (50 mM Tris-HCl, 300 mM NaCl, pH 8.0), and lysed by sonication. The lysate was clarified by centrifugation (16,000 × g, 30 min, 4 °C), and the supernatant was filtered and applied to a 15 mL gravity-flow column containing Ni-NTA agarose resin (HisPur Ni-NTA, Thermo Fisher Scientific). After washing with 10 column volumes (CV) of wash buffer (50 mM Tris-HCl, 300 mM NaCl, 25 mM imidazole, pH 8.0) followed by 5 CV of PBS, bdSUMO protease (50 µM) was added directly to the column. The column was incubated overnight at 4 °C with gentle shaking (60 rpm) to release the cleaved ACE2D protein. The eluted protein was subsequently purified by size-exclusion chromatography on a Superdex 75 16/600 column (Cytiva) equilibrated in PBS.

### Next generation sequencing

DNA libraries (four HSS RBD libraries at three stages: unsorted mutagenized libraries, second round of affinity maturation, fourth round of affinity maturation, and five LSS RBD libraries) were amplified using KAPA HiFi and purified using the NucleoSpin Gel and PCR Clean-up Kit. Barcodes were added as part of the amplification primers (primers are described in Supplementary data file 1 and barcodes are listed in Table S3) and the number of minimal PCR amplification was determined by qPCR. PCR amplicons were sequenced on Illumina sequencers as 2 × 150 nt paired-end reads, and additionally as 2 × 250 nt paired-end reads to ensure overlap of forward and reverse reads for all amplicons. The PCR products already contained P5 and P7 Illumina sample barcodes; hence, demultiplexing was done using the standard Illumina bcl2fastq tool. Notably, the sequencing libraries were diluted with samples from other customers to avoid ghost signals. For efficiency, the sequencing reads were sorted and unified using UNIX sort and uniq utilities and their incidence counts were recorded in a FASTA ID tag along their SHA256 checksum, allowing to discern from each other groups of exactly the same sequences and reduced the data down to 8–20 % of the original FASTA size. The sequences representing each original cluster were aligned using NCBI blastn with the following command: “blastn -task blastn -reward 2 -max_hsps 1 -num_alignments 1 -word_size 4 -dust no -evalue 1e-5 …” to the Wuhan-1 reference S protein portion spanning only the amplicon region (excluding primer sites) and starting in the +1 reading frame. This effectively restricted Illumina reads to the amplicon region and chopped away the primers. Notably, the NCBI’s BLAST command line re-enables the original while thorough blastn algorithm with the most sensitive exact match requirement set down to the minimum of only 4 nucleotides and increases reward bonus for a match to overcome mismatches close to the ends of the alignment. The BLASTn output was parsed using Unix awk and further processed by a pipeline of Python and shell scripts: drop_erroneous_insertions.py, reversecomplement_reads_on_minus.py, and fix_SARS-CoV2_S-protein_indel_misalignments.sh. These scripts removed single-nucleotide insertions disrupting the reading frame, reverse-complemented sequences on the minus strand, and added leading/trailing dashes to standardize sequence length to 3822 nt (the full *SARS-CoV-2 S* gene). Further details and InDel re-alignment are described in https://github.com/host-patho-evo/mutation_scatter_plot. The Wuhan S protein coding sequence (GenBank accession MN908947.3) is spanning from the initiator ATG to the termination codon at position 3822 and, is representing 1274 codons. We aligned the obtained Illumina sequences using NCBI’s BLASTN to the respective reference sequence and applied a similarity cutoff of > 84%, which included 99% of the data while we discarded low-quality and short sequences.

For the follow-up analysis we developed in Python two programs which are now available at https://github.com/host-patho-evo/mutation_scatter_plot. Briefly, the multiFASTA files with incidence counts followed by a SHA256 checksum were parsed by calculate_codon_frequencies.py using biopython (version 1.83) which iterated over the reference sequence (to respect reading-frame) and calculated frequencies of codons in a particular 3nt wide column of the multi-FASTA ALN file. The results were stored in a TAB-separated TSV file for easy post-processing by another Python-based utility named mutation_scatter_plot.py, which discards codons containing unknown (N) nucleotides from the TSV files using Python Pandas library and finally draws interactive figures using Matplotlib and Bokeh graphical libraries. To color the figures, we used the widely used BLOSUM62 matrix from https://www.ncbi.nlm.nih.gov/IEB/ToolBox/C_DOC/lxr/source/data/BLOSUM62 to discern evolutionarily conservative amino acid changes from those more different to the parental residue. The color palette “coolwarm_r” visible at https://i.sstatic.net/cmk1J.png we used for drawing ranges from red to blue (in dark red should be rather pronounced change while in dark blue should be functionally similar amino acid change). By default, mutation_scatter_plot.py omits codon changes with frequency lower than 0.001 (below one 0.1%) or when in amino acid mode it omits cumulative codon changes summed up at the resulting amino acid frequency below 0.01 (below 1%). The FASTA, TSV, HTML with JavaScript, PNG, JPG and PDF files for each sample were deposited at Zenodo.org under DOI: 10.5281/zenodo.19475241 accession. Our software is available at https://github.com/host-patho-evo/mutation_scatter_plot and Zenodo repository v0.3 DOI:10.5281/zenodo.18550271 including the list of used GISAID sequences. The interactive HTML files are directly accessible along JPG preview images at https://host-patho-evo.github.io/mutation_scatter_plot.

### Simulations of evolutionary trajectories

Simulations were performed as detailed in Fig. 5 and adjacent text using a Python script generated for this purpose. The code is available at https://zenodo.org/records/18554273.

### Reporting summary

Further information on research design is available in the Nature Portfolio Reporting Summary linked to this article.

## Supplementary information


Supplementary Information
Peer Review file
Description of Additional Supplementary Files
Supplementary Data 1
Reporting Summary


## Source data


Source Data 1
Source Data 2
Source Data 3
Source Data 4
Source Data 5


## Data Availability

Source data for all main figures are provided in the accompanying Source Data files. All other data supporting the findings of this study on SARS-CoV-2 evolution are available within the article, in the Supplementary Information, and via the EU Open Research Repository Zenodo under DOI: 10.5281/zenodo.19475241, including the list of GISAID sequence headers used in evolutionary comparisons. Full sequences and their metadata are available through GISAID. Data related to in vitro evolution of SARS-CoV-1 are subject to restricted access due to considerations of potential dual-use research of concern (DURC). Access can be granted upon request to the corresponding author, Dr. Jiri Zahradnik. Source data are provided with this paper.

## Code availability

The codon mutation simulator (v0.1) is available at Github https://github.com/gideonschreiber/codon_mutation_simulator and archived at https://zenodo.org/records/18554273^82^. The tools (v0.3) for counting incidence of codons within DNA multiple sequence alignment and for drawing scatter plots of the relative frequencies of each codon utilized in a site (or alternatively, of the encoded amino acids) are available at Github https://github.com/host-patho-evo/mutation_scatter_plot and archived at https://zenodo.org/records/18550271^83^.
