# Author Correction: Stringent selection drives convergence toward omicron-like SARS-CoV-2 receptor-binding motifs

**DOI:** 10.1038/s41467-026-77179-8

**Published:** 2026-08-26

**Authors:** Aviv Shoshany, Ruojin Tian, Miguel Padilla-Blanco, Adam Hruška, Aditi Konar, Katarína Baxová, Eyal Zoler, Martin Mokrejš, Gideon Schreiber, Jiří Zahradník

**Affiliations:** 1 Department of Biomolecular Sciences, Weizmann Institute of Science, Rehovot, Israel; 2 First Faculty of Medicine, Charles University, BIOCEV center, Vestec, Czechia; 3 Viral Immunology Lab, Molecular Biomedicine Department, Margarita Salas Center for Biological Research (CIB-CSIC), Madrid, Spain; 4 Institute of Organic Chemistry and Biochemistry, Czech Academy of Sciences, Prague, Czechia; 5 Institute of Biotechnology, Czech Academy of Sciences, BIOCEV center, Vestec, Czechia

**Keywords:** SARS-CoV-2, Molecular evolution, Phylogenetics

Correction to: *Nature Communications* 10.1038/s41467-026-72312-z, published online 25 April 2026

In the version of this article initially published, in Fig. 6a and associated Source Data, there were duplicate values due to errors during the normalization process. The figure and source Data are now updated in the HTML and PDF versions of the article. For comparison, the original Fig. 6 is available as Supplementary Information accompanying this article.

## Supplementary information

Original Fig. 6

