# Supplementary material for: Stringent selection drives convergence toward omicron-like SARS-CoV-2 receptor-binding motifs
Source: Nat Commun. 2026 Apr 25;17:5712. doi: 10.1038/s41467-026-72312-z (PMC13324855; doi:10.1038/s41467-026-72312-z)
Supplement: Supplementary file 1 — Supplementary Information [file 41467_2026_72312_MOESM1_ESM.pdf]

Supporting Information to:

Stringent Selection Drives Convergence Toward Omicron-like SARS-CoV-2 Receptor-Binding Motifs

Aviv Shoshany ^{1*}, Ruojin Tian ^{2*}, Miguel Padilla-Blanco ^{2,3*}, Adam Hruška ², Aditi Konar ², Katarina Baxova ^{2,4}, Eyal Zoler ¹, Martin Mokrejš ^{2,5}, Gideon Schreiber ^{1#} and Jiří Zahradník ^{2#}

* Contributed equally

# Correspondence:

gideon.schreiber@weizmann.ac.il

jiri.zahradnik@lf1.cuni.cz

Affiliations

- 1) Department of Biomolecular Sciences, Weizmann Institute of Science, Herzl St. 234, Rehovot 7610001, Israel
- 2) First Faculty of Medicine, Charles University, BIOCEV center, Prumyslova st. 595, Vestec 252 50, Czechia
- 3) Viral Immunology Lab, Molecular Biomedicine Department, Margarita Salas Center for Biological Research (CIB-CSIC), Madrid, Spain
- 4) Institute of Organic Chemistry and Biochemistry, Czech Academy of Sciences, Flemingovo sq. 542/2, Prague 160 00, Czechia
- 5) Institute of Biotechnology, Czech Academy of Sciences, BIOCEV center, Prumyslova st. 595, Vestec 252 50, Czechia

Supporting information part PS1 - platform for parallelized *in vitro* evolution

The pJYDC4 plasmid was created using a cut-and-paste replacement of the eUnaG2 reporter with SpyCatcher003. Similarly, the pJYDC6 plasmid was constructed using a designed monomeric avidin (DeMA). The design of this reporter was based on the PDB structure 1avd of rhizavidin. The design process was identical to the preparation of the eUnaG2 and DnbALFA reporters in Zahradník, J. *et al.* A Protein-Engineered, Enhanced Yeast Display Platform for Rapid Evolution of Challenging Targets. *ACS Synthetic Biology* 10, 3445-3460 (2021). [https://doi.org:10.1021/acssynbio.1c00395](https://doi.org/10.1021/acssynbio.1c00395)

>SpyCatcher003_S.cer_optimized

ATGGTTACCACTGAGCGGTCTGAGTGGTGAACAGGGTCCGAGCGGTGATATGACCACCGAAGAAGATAGCG
CAACCCATATCAAATTTAGCAAACGTGATGAAGATGGTCGTGAAGTGGCAGGCGCAACCATGGAAGTGCCTGA
TAGCAGCGGTAAACCATTAGCACCTGGATTAGTATGGTCACGTGAAAGATTTTATCTGTATCCGGGTAAA
TATACCTTCGTTGAAACCGCAGCACCGGATGGTTATGAAGTTGCAACCCCGATTGAATTCACCGTTAACGAAG
ATGGCCAGGTTACCGTTGATGGTGAAGCAACCGAAGGTGATGCACATACC

>DeMA_S.cer_optimized

TTTCGATGCCTCTAACTTCAAGGACTTCTCTTCTATTGCTGGTACTTCTACTACCTGGCAAAATCAACATGGTT
CCACTATGGTTATCACCGTTGATTCTCAAGGTAACGTTTCTGGTCAATACGTTAATAGAGCTGAAGGTACTGG
TTGTCAGAAATTTCCATATCCATTGACTGGTTGGGTTAACGGTACTTTTATTGATTTCTCTGTTACCTGGAAC
AACTCTACCGAAACTGTAACCTGTTACTCAATGGACTGGTTACGCTCAAGTTAATGGTAACAACACTGAAA
TCGTTACCGATTGGAAGTTGGTTTATGAAGGTCCATCTGGTCCAGCTATTGGCAAGGTCAAGATACTTTTCA
ATACGTCCCACTACCGAG

pJYDC-barcF-RBDo-Wu plasmid sequence segment with expression of whole yeast display cassette used: eUnaG2 – Aga2p – barcode_F_fwd - RBDo-Wu_sequence - barcode_F_rev.

Left and right barcodes were integrated flanking the expression cassette within the expression construct. Their sequences are in the Table S1

>Expression_construct

ATGAGGTTCCCATCTATTTTACCGCTGTTGTTTTGCTGCTTCTTCTGCTTTGGCTGCTCCAGCTAATGGTATGTTAGA
AAAATTTGTTGGCACCTGGAAGATCGAATCCTCTGAAAATTTTGGTGAATACTTGAAGGCTATCGGTGCCCCAAAAGAAT
TGGCTGATGCTGGTGATGCTACTACTCCAGTCTTGTACATTTCTCAAAAAGGATGGTGATAAGATGACCGTCAAGATTGAA
AACGGTCCACCAACTTTTTTGGATACCCAAGTTTCTTCAAGTTGGGTGAAGAATTCGACGAATTTCCATCCGATAGAAG
AAAGGGTGTAAAGTCCGTTGTTAACTTGTCTGGTGAGAAGTTGGTTTACGTTCAAAAAGTGGGATGGTAAAGAAACCACTT
ACGTCAGAGAAATCAAGGACGGTAAATTGGTTGTTACTTTGACCATGGGTGATGTTGTTGCTGTTAGATCTTATAGAAGG
GCCTCTGAAGTTTCTGCACAGGAAGTACCAACTATATGCGAGCAAATCCCCTCACCAACTTTAGAAATCGACGCCGTACTC
TTTGTCAACGACTACTATTTTGGCCAACGGGAAGGCAATGCAAGGAGTTTTTGAATATTACAAATCAGTAACGTTTGTCA
GTAATTGCGGTTCTCACCCCTCAACAAGTAGCAAAGGCAGCCCCATAAACACACAGTATGTTTTTAAGGACAATAGCTCG
ACGATTgaaggtagatacccatagcagcttccagactacgctctgcaggctagtgggtggaggaggtctgggtggaggcgg
tagcggaggcggaCAAGGTACCGGAAGTACAAGTgctagccatattgggtTGCCCTTTTGGTGAAGTTTTTAACGCCACCA
GATTTGCATCTGTTTATGCTTGAACAGGAAGAGAATCAGCAACTGTGTTGCTGATTATTCTGTCTATATAAATTCGCA
TCATTTTCCACTTTTAAGTGTTATGGAGTGTCTCCTACTAAATTAATGATCTCTGCTTTACTAATGTCTATGCAGATTC
ATTTGTAATTAGAGGTGATGAAGTCAGACAAATCGCTCCAGGGCAAAGTGGAAAGATTGCTGATTATAAATTATAAATTAC
CAGATGATTTTACAGGCTGCGTTATAGCTTGAATCTAACAATCTTGATTCTAAGGTTGGTGGTAATTATAAATTACCTG
TATAGATTGTTTAGGAAGTCTAATCTCAAACCTTTTGAGAGAGATATTTCAACTGAAATCTATCAGGCCGGTAGCACACC
TTGTAATGGTGTGAAGGTTTTAATTGTTACTTTTCTTTTCAATCATATGGTTTCCAACCCACTAATGGTGTGGTTACC
AACCATACAGAGTAGTAGTACTTTCTTTTGAAGTTCTACATGCACCAGCAACTGTTTGTGGACCTAAAggatccAGATCC
GGAAATGGCAGTACCgaacaaagcttatttctgaagaggacttgtaa

- in red short AppS4 leader sequence; in green eUnaG2 reporter protein; in bold Aga2p sequence; yellow background highlighted sequences – fwd and rev barcodes; in blue RBD sequence; in magenta c-myc sequence

Table S1. Expression cassette barcodes for identification of plasmids in parallelized *in vitro* evolution

Barcode name	Barcode sequence	AA sequence
A_fwd	AGCAACGGTACTGGATCTAGT	SNGTGSS
A_rev	GGAGGCAACTCAAGTGGAAC	GGASSGT
B_fwd	AACACTTCAGGAGGTAGAGGA	NTSGGRG
B_rev	ACCGGATCTAATGGTTCAGGT	TGSNGSG
C_fwd	TCCTCTGGAACATAATGGCACA	SSGTNGT
C_rev	GGTTCAACTGGCGGTTCTCTCG	GSTGGSS
D_fwd	acaaacgggttccggcacaggc	TNGSGTG
D_rev	aataccgggttctggaaccgca	NTGSGTA
E_fwd	AGTGGCAATGCAACTGGTAGC	SGNATGS
E_rev	TCGGGCAATAGTGGATCATCT	SGNSGSS
F_fwd	CAAGGTACCGGAAGTACAAGT	QGTGSTS
F_rev	AGATCCGGAAATGGCAGTACC	RSGNGST
G_fwd	aacagcaccggcggcagcggc	NSTGGSG
G_rev	gctcgcgctgccgggtgcggcc	GRTGSAS
H_fwd	accaccaacagcagcggcagc	TTNSSGS
H_rev	gccgcccgtgttcgcgggtgcg	RTANSGG

- lowercase letters show barcodes that were not used due to potential expression influence

Table S2. Initial RBD sequences, barcodes and plasmids

Original SARS-CoV2 sequence<br>(Low Stringency selections, LSS)	Barcode	Plasmid (1 st round)
isolate Wuhan-Hu-1 (MN908947.3)	FF	pJYDC3
isolate SARS-CoV-2/human/ITA/VA-English-2021-01-22/2021 (MW521144.1)	CC	pJYDC3
isolate SARS-CoV-2/human/ITA/VA-South Africa 2021-02-03/2021 (MW560269.1)	AA	pJYDC3
MW560269.1 + Q498R (Abbreviated RY)	BB	pJYDC3
Omicron BA.1; isolate SARS-CoV-2/human/USA/TX-CDC-ASC210646153/2022 (OM652834.1)	EE	pJYDC3
Original SARS-CoV2 sequence<br>(High Stringency selections, HSS)	Barcode	Plasmid (1 st round)
isolate Wuhan-Hu-1 (MN908947.3)	AA	pJYDC1
isolate Wuhan-Hu-1 (MN908947.3) + I358F mutation introduced by SDM	BB	pJYDC3
Omicron BA.1; isolate SARS-CoV-2/human/USA/TX-CDC-ASC210646153/2022 (OM652834.1)	C_fwd	pJYDC1
Omicron BA.2, isolate SARS-CoV-2/human/USA/CA-CDC-LC0582623/2022 (ON373904.1)	C_rev	pJYDC3

- combination of barcodes A_fwd + A_rev is simplified to AA; C_fwd means that only forward barcode was used in this case, C_rev means that only reverse barcode was used in this case

Table S3. Sample specific barcodes used in Illumina paired-end deep sequencing

Sample	Forward primer	Fwd-seq-barcode (5'-end)	Reverse primer	Rev-seq-barcode (5'-end)
HSS WT lib non-sorted	TTGCTGATTATAATTATAA<br>ATTACCAGATGATTTT	Not used	CAAGTCCTCTTCAGAAAT<br>AAGCTTTTGTTTCGGATCC	AGTTCCACTTGAGTT<br>GCCTCC
HSS I358F non-sorted	TTGCTGATTATAATTATAA<br>ATTACCAGATGATTTT	Not used	CAAGTCCTCTTCAGAAAT<br>AAGCTTTTGTTTCGGATCC	TCCTGAACCATTAGA<br>TCCGGT
HSS BA1 non-sorted	TTGCTGATTATAATTATAA<br>ATTACCAGATGATTTT	Not used	CAAGTCCTCTTCAGAAAT<br>AAGCTTTTGTTTCGGATCC	TGTGCCATTAGTTCC<br>AGAGGA
HSS BA2 non-sorted	TTGCTGATTATAATTATAA<br>ATTACCAGATGATTTT	Not used	CAAGTCCTCTTCAGAAAT<br>AAGCTTTTGTTTCGGATCC	CGAGGAACCGCCAG<br>TTGAACC
HSS WT lib 2	TTGCTGATTATAATTATAA<br>ATTACCAGATGATTTT	Not used	CAAGTCCTCTTCAGAAAT<br>AAGCTTTTGTTTCGGATCC	AGTTCCACTTGAGTT<br>GCCTCC
HSS I358F lib 2	TTGCTGATTATAATTATAA<br>ATTACCAGATGATTTT	Not used	CAAGTCCTCTTCAGAAAT<br>AAGCTTTTGTTTCGGATCC	TCCTGAACCATTAGA<br>TCCGGT
HSS BA1 lib 2	TTGCTGATTATAATTATAA<br>ATTACCAGATGATTTT	Not used	CAAGTCCTCTTCAGAAAT<br>AAGCTTTTGTTTCGGATCC	TGTGCCATTAGTTCC<br>AGAGGA
HSS BA2 lib 2	TTGCTGATTATAATTATAA<br>ATTACCAGATGATTTT	Not used	CAAGTCCTCTTCAGAAAT<br>AAGCTTTTGTTTCGGATCC	CGAGGAACCGCCAG<br>TTGAACC
HSS WT lib 4	TTGCTGATTATAATTATAA<br>ATTACCAGATGATTTT	Not used	CAAGTCCTCTTCAGAAAT<br>AAGCTTTTGTTTCGGATCC	AGTTCCACTTGAGTT<br>GCCTCC
HSS I358F lib 4	TTGCTGATTATAATTATAA<br>ATTACCAGATGATTTT	Not used	CAAGTCCTCTTCAGAAAT<br>AAGCTTTTGTTTCGGATCC	TCCTGAACCATTAGA<br>TCCGGT
HSS BA1 lib 4	TTGCTGATTATAATTATAA<br>ATTACCAGATGATTTT	Not used	CAAGTCCTCTTCAGAAAT<br>AAGCTTTTGTTTCGGATCC	TGTGCCATTAGTTCC<br>AGAGGA
HSS BA2 lib 4	TTGCTGATTATAATTATAA<br>ATTACCAGATGATTTT	Not used	CAAGTCCTCTTCAGAAAT<br>AAGCTTTTGTTTCGGATCC	CGAGGAACCGCCAG<br>TTGAACC
LSS WT	GTACAAGTGCTAGCCATA<br>TGGGTTGCC	GCCCCT<br>CT	GGCCTGATAGATTTCAGT<br>TGAAATATCTCTC	TTCTCAGA
LSS Alpha	ATGGCACAGCTAGCCATA<br>TGGGTTG	AACCTT<br>AC	GGCCTGATAGATTTCAGT<br>TGAAATATCTCTC	CCGTACAC
LSS Beta	GGATCTAGTGCTAGCCAT<br>ATGGGTTG	GACAGC<br>CC	ACCGGCCTGATAGATTTC<br>AGTTG	TGCCGTGG
LSS RBD-v48	GTAGAGGAGCTAGCCATA<br>TGGGTTG	ACGCAA<br>TC	ACCGGCCTGATAGATTTC<br>AGTTG	GCACCTAG
LSS BA.1	CTGGTAGCGCTAGCCATA<br>TGGGTAC	AGATGA<br>GT	GGCCTGATAGATTTCAGT<br>TGAAATATCTCTC	CTCACAAT

Table S4. Codon frequencies upon HSS evolution of the RBM

Position	WT aa	BA.1 aa	BA.2 aa	Mutant aa	WT codon	Mutant codon	Freq Lib WT	Freq WT 2nd	Freq WT 4th	Freq Lib I358F	Freq I358F 2nd	Freq I358F 4th	Freq Lib BA.1	Freq BA.1 2nd	Freq BA.1 4th	Freq Lib BA.2	Freq BA.2 2nd	Freq BA.2 4th
440	N	K	K	K	AAT	AAG	5.4E-04	5.3E-01	6.8E-01	3.1E-04	5.5E-01	8.6E-01	9.8E-01	9.9E-01	9.7E-01	9.7E-01	9.9E-01	9.7E-01
444	K			T	AAG	ACG	3.8E-03	2.1E-01	1.9E-01	3.7E-03	5.5E-01	7.6E-01	4.1E-03	2.7E-01	1.1E-01	4.1E-03	9.7E-03	8.9E-03
444	K			R	AAG	AGG	1.8E-03	3.2E-01	4.6E-01	3.1E-03	2.2E-03	1.2E-03	8.4E-04	1.5E-01	6.7E-02	3.2E-03	6.7E-01	7.5E-01
445	V			V	GTT	GTC	2.2E-03	2.3E-03	7.4E-03	4.0E-03	3.0E-03	7.8E-03	1.0E-03	3.5E-01	5.7E-01	3.8E-03	1.7E-03	6.9E-03
446	G	S	G	S	GGT	AGT	2.3E-03	8.6E-03	1.2E-02	2.8E-03	2.0E-02	3.4E-02	9.9E-01	5.4E-01	7.8E-01	2.9E-03	2.2E-03	5.5E-03
450	N			D	AAT	GAT	1.5E-03	6.2E-01	6.9E-01	2.1E-03	9.3E-04	4.3E-03	9.0E-04	5.6E-01	7.3E-01	2.1E-03	7.1E-01	8.2E-01
452	L			K	CTG	AAG	8.0E-06	1.5E-02	1.6E-01	7.0E-06	1.5E-05	1.6E-05	4.0E-06	4.6E-03	3.6E-02	1.1E-05	5.5E-03	9.8E-02
452	L			M	CTG	ATG	2.6E-03	6.1E-01	5.3E-01	3.1E-03	1.8E-03	1.2E-03	2.3E-03	5.6E-01	6.9E-01	3.8E-03	7.1E-01	7.2E-01
452	L			R	CTG	CGG	8.9E-04	2.8E-01	2.7E-01	7.7E-04	5.8E-01	9.1E-01	7.5E-04	4.0E-01	2.6E-01	8.5E-04	1.3E-02	1.3E-02
453	Y			F	TAT	TTT	2.2E-03	1.6E-02	6.4E-03	3.4E-03	1.2E-01	2.1E-02	1.3E-03	5.9E-03	3.8E-03	3.2E-03	1.4E-01	1.7E-01
460	N			K	AAT	AAA	1.8E-03	3.0E-01	2.3E-01	2.2E-03	5.9E-01	9.2E-01	1.4E-03	3.4E-01	2.3E-01	2.1E-03	1.5E-01	1.7E-01
460	N			K	AAT	AAG	2.7E-04	6.3E-01	7.4E-01	2.4E-04	9.2E-03	2.9E-02	2.3E-04	5.8E-01	7.4E-01	2.7E-04	7.2E-01	8.1E-01
477	S	N	N	N	AGC	AAC	4.8E-03	8.4E-01	9.8E-01	5.1E-03	7.5E-01	9.8E-01	9.9E-01	9.9E-01	9.9E-01	9.8E-01	9.9E-01	9.9E-01
478	T	K	K	K	ACA	AAA	1.3E-03	8.1E-01	9.7E-01	1.1E-03	6.4E-01	9.5E-01	9.9E-01	9.9E-01	9.8E-01	9.7E-01	9.9E-01	9.7E-01
482	G			R	GGT	CGT	3.8E-04	1.9E-01	2.6E-01	2.9E-04	7.0E-06	7.2E-04	3.2E-04	1.0E-05	2.9E-03	3.5E-04	4.0E-06	2.0E-05
484	E			R	GAA	AGA	3.7E-05	4.6E-01	9.0E-01	1.6E-05	7.3E-03	1.0E-01	3.1E-05	6.3E-01	9.0E-01	2.4E-05	6.0E-01	7.1E-01
484	E	A	A	A	GAA	GCA	9.4E-04	4.0E-01	6.3E-02	1.1E-03	5.7E-01	6.4E-01	9.8E-01	3.6E-01	6.2E-02	9.8E-01	3.9E-01	2.7E-01
486	F			V	TTT	GTT	3.0E-03	2.7E-01	2.6E-02	2.6E-03	4.2E-01	5.2E-02	3.1E-03	1.2E-01	1.3E-02	2.6E-03	3.8E-03	2.5E-03
493	Q			K	CAA	AAA	6.5E-04	3.0E-04	5.0E-02	6.8E-04	8.6E-03	1.7E-01	2.0E-06	5.9E-04	8.0E-03	2.0E-06	1.9E-04	8.5E-05
493	Q	R	R	R	CAA	CGA	2.5E-03	1.0E-02	2.7E-01	4.0E-03	1.1E-01	6.5E-01	9.9E-01	5.7E-02	1.6E-02	9.8E-01	1.8E-01	1.5E-01
493	Q			G	CAA	GGA	3.0E-06	1.0E-06	2.5E-04	2.0E-06	3.0E-05	3.0E-04	7.1E-04	1.1E-01	7.1E-02	7.0E-04	1.5E-01	3.9E-01
496	G	S	G	S	GGT	AGT	1.6E-03	6.5E-03	7.7E-03	2.0E-03	3.5E-02	1.3E-02	9.9E-01	2.0E-01	6.7E-02	2.2E-03	4.0E-04	1.5E-03
498	Q			H	CAA	CAT	2.5E-03	9.1E-03	2.5E-05	4.4E-03	2.4E-01	2.9E-02	2.6E-05	1.2E-04	4.4E-05	3.5E-05	1.8E-05	3.0E-06
498	Q	R	R	R	CAA	CGA	3.4E-03	9.1E-01	9.0E-01	5.2E-03	7.0E-01	9.2E-01	9.9E-01	9.9E-01	9.5E-01	9.8E-01	9.8E-01	9.8E-01
501	N			T	AAT	ACT	5.2E-04	2.5E-03	3.0E-06	6.1E-04	2.3E-01	3.5E-02	2.0E-06	6.8E-05	2.6E-05	3.0E-06	2.3E-05	1.0E-06
501	N	Y	Y	Y	AAT	TAT	1.8E-03	9.6E-01	9.8E-01	2.4E-03	7.3E-01	9.6E-01	9.9E-01	9.9E-01	9.9E-01	9.8E-01	9.9E-01	9.8E-01
505	Y	H	H	H	TAC	CAC	2.1E-03	3.6E-01	6.2E-02	3.6E-03	1.2E-01	3.6E-02	9.9E-01	9.6E-01	6.6E-01	9.8E-01	9.9E-01	9.5E-01

Mutant codon frequencies (Freq), as determined from NGS of the non-selected first library (Lib) in comparison to the 2nd and 4th round of library propagation and selection (2nd and 4th). Codons with frequencies of >0.1 in at least one library are shown. Mutants are in relation to WT. Blue is for low and red for high frequencies.

Table S5. Frequencies of amino-acid mutations in RBD of the Spike protein of SARS-CoV-2 compared to frequencies of mutations in HSS selected clones

Residue	WT	BA1	BA2	var	1<br>Frequency<br>SARS-<br>CoV-2	2<br>Frequency<br>SARS-<br>CoV-2<br>(2023+)	3<br>Frequency<br>HSS WT	4<br>Frequency<br>HSS I358F	5<br>Frequency<br>HSS BA.1	6<br>Frequency<br>HSS BA.2
440	N	K	K	K	0.638	0.935	0.684	0.857	0.973	0.974
444	K			T	0.037	0.000	0.198	0.762	0.109	0.009
445	V			P	0.079	0.000	0.002	0.024	0.000	0.000
445	V			H	0.014	0.283				
446	G	S	G	S	0.444	0.819	0.012	0.034	0.778	0.005
446	G			G	0.543	0.181	0.961	0.940	0.453	0.975
450	N			D	0.018	0.298	0.694	0.004	0.733	0.816
452	L			R	0.269	0.167	0.265	0.913	0.256	0.013
452	L			Q	0.023	0.001	0.001	0.003	0.000	0.019
452	L			W	0.014	0.292				
455	L			S	0.013	0.282				
456	F			L	0.020	0.341	0.001	0.011	0.000	0.000
460	N			K	0.156	0.902	0.973	0.945	0.976	0.974
477	S	N	N	N	0.867	0.951	0.978	0.980	0.989	0.990
478	T	K	K	K	0.927	0.849	0.971	0.951	0.983	0.974
478	T			R	0.011	0.096				
481	N			K	0.010	0.298	0.011	0.001	0.006	0.001
484	E			R	0.001	0.002	0.900	0.105	0.903	0.709
484	E	A	A	A	0.860	0.651	0.063	0.641	0.062	0.271
484	E			K	0.018	0.290	0.003	0.078	0.003	0.002
486	F			V	0.108	0.114	0.026	0.052	0.013	0.003
486	F			P	0.068	0.777				
490	F			S	0.070	0.506	0.017	0.035	0.004	0.198
493	Q	R	R	R	0.675	0.000	0.273	0.670	0.016	0.146
496	G	S	G	S	0.541	0.000	0.008	0.013	0.067	0.002
496	G			G	0.453	0.999	0.972	0.951	0.794	0.967
498	Q	R	R	R	0.872	0.951	0.972	0.953	0.985	0.994
501	N	Y	Y	Y	0.894	0.953	0.983	0.958	0.994	0.993
505	Y	H	H	H	0.851	0.950	0.062	0.036	0.667	0.982

WT corresponds to the amino-acid in the isolate Wuhan-Hu-1 (MN908947.3) sequence. In 5th column named “var” is the aa which frequencies are shown in 1-6 for the corresponding variants or selections.

¹ Frequencies calculated from GISAID database in spikenuc1207 released by GISAID on Dec 7 2024.

² Frequencies calculated from GISAID database in spikenuc1207, deposited after 2023.

³ Frequencies as calculated from HSS of WT.

⁴ Frequencies as calculated from HSS of WT+I358F.

⁵ Frequencies as calculated from HSS of BA.1.

⁶ Frequencies as calculated from HSS of BA.2. Only frequencies >0.01 are shown. Empty field (ND) is for HSS frequencies which were <0.01.

Table S6 – Frequencies of combinations of mutations in the RBD between residues 498 and 501.

Library name	Sequence<br>498-QPTN-501<br>Occurrence (%)	Sequence<br>498-QPTY-501<br>Occurrence (%)	Sequence<br>498-HPTN-501<br>Occurrence (%)	Sequence<br>498-HPTY-501<br>Occurrence (%)	Sequence<br>498-HPTT/S-501<br>Occurrence (%)	Sequence<br>498-RPTY-501<br>Occurrence (%)	Other notable than previous > 0.01	Total sequences fully spanning the motif*
WT 7th lib LSS	0.280	14.030	4.420	0.470	56.210	15.060	('HPTS', 6.99),<br>('QPTT', 0.69),<br>('HPST', 0.31),<br>('RPPT', 0.24),<br>('HPTA', 0.17),<br>('QPTF', 0.12),<br>('HPTT', 0.1)	7865410
Alpha 7th lib LSS	1.430	38.630	11.920	0.460	6.910	36.190	('HPTS', 2.37),<br>('HPTA', 0.48),<br>('QPTT', 0.42),<br>('QPTF', 0.16),<br>('RPAV', 0.13)	12873967
Beta 7th lib LSS	0.220	2.940	0.230	0.010	0.080	93.840	('RPAV', 0.37),<br>('QPTF', 0.33),<br>('RPTD', 0.22),<br>('RPTF', 0.16),<br>('RPSV', 0.12),<br>('RSTY', 0.11)	20922
RBD v48 7th lib LSS	0.400	0.450	0.070	0.000	0.100	97.750	('RPAV', 0.24),<br>('RPSV', 0.2),<br>('RPTD', 0.1)	3211512
RBD BA.1 7th lib LSS	0.430	0.100	0.030	0.000	0.010	97.470	('RPAV', 0.54),<br>('RPSV', 0.51),<br>('LPTY', 0.14),<br>('RPTD', 0.13)	6918311
WT 4th lib HSS	0.910	0.130	0.000	0.000	0.000	96.360	('RRTY', 0.55),<br>('RHTY', 0.35),<br>('RSTY', 0.26),<br>('RPAV', 0.25),<br>('RPSV', 0.24),<br>('RPTN', 0.11)	749658
I358F 4th lib HSS	0.010	0.240	0.030	0.010	2.860	94.580	('YPTT', 0.39),<br>('RPSV', 0.18),<br>('RPAV', 0.18),<br>('RTTY', 0.17),<br>('LPTY', 0.15),<br>('RHTY', 0.13),<br>('RPTC', 0.12),<br>('RPTT', 0.11),<br>('RRTY', 0.11)	756757
BA.1 4th lib HSS	0.130	0.090	0.000	0.000	0.000	97.920	('RPAV', 0.37),<br>('RPSV', 0.36)	614899
BA.2 4th lib HSS	0.000	0.120	0.000	0.000	0.000	98.070	('RPSV', 0.34),<br>('RHTY', 0.32),<br>('RRTY', 0.16),<br>('RPAV', 0.14),<br>('RPTH', 0.14),<br>('RPTF', 0.13),<br>('RSTY', 0.12)	817816
GISAID (Spikenuc_1207)	37.720	11.580	0.000	0.000	0.000	47.990		646698

WT corresponds to the amino-acid in the isolate Wuhan-Hu-1 (MN908947.3) sequence and was used as a reference with a sequence 498-QPTN-501.

Table S7. Binding affinities and sequences of clones from Fig. 4.

Lineages	KD (nM)	339	345	346	356	358	368	371	373	375	376	378	392	405	408	417	428	440	444	445	446	450	452	459	460	462	470	477	478	483	484	486	493	496	498	501	505	
WT aa	4.36 (0.3)	G	T	R	K	I	L	S	S	S	T	K	F	D	R	K	D	N	K	V	G	N	L	S	N	K	T	S	T	V	E	F	Q	G	Q	N	Y	
WT I358F	4.24 (0.5)					F																																
Alpha	4.4 (0.5)																																				Y	
Beta	3.15 (0.3)															N														K						Y		
BA.1	5.9 (0.3)	D					L	P	F						N	K		S									N	K		A		R	S	R	Y	H		
BA.2	7.0 (0.6)	D					F	P	F	A			N	S	N	K											N	K		A		R		R	Y	H		
XBB.1.5	1.9 (0.2)	H	T			I	F	P	F	A			N	S	N	K	P	S						K			N	K		A	P			R	Y	H		
**LSS**																																						
WT-c35	2.737 (0.33)																																		G	H		
WT-cJ452	2.956 (0.23)					F																		T	K								G		Y			
WT-cJ428	2.613 (0.18)																						Q										L	G	H			
WT-c46	1.45 (0.14)																																	G	H	T		
WT-c71	2.316 (0.11)												E			K								K					A				G	H	S			
Alpha-c46	3.834 (0.34)																																	G	R	Y		
Alpha-c49	2.856 (0.4)																																	G		Y		
Alpha-c71	3.383 (0.4)																										N							G		Y		
Alpha-cJ71	3.804 (0.4)																											N							G		Y	
Beta-c56	2.9 (0.3)														N														N		K			G	R	Y		
Beta-c72	3.4 (0.3)														N	K								K							K			G	R	Y		
BA1-cJ43	4.235 (0.15)	D					L	P			I			N	K									E	N	K		A		R		R	Y	H				
BA1-cJ44	3.659 (0.12)	D					L	P			S			I	K		S									N	K		A		R	G	R	Y	H			
BA1-cJ715	4.589 (0.1)	D					L	P			L			I	K		S										N	K		A				R	Y			
BA1-c72	3.637 (0.08)	D	N				L	P						N	G	K										A	N	K		A				R	Y	H		
BA1-c73	1.973 (0.1)	A					L	P			R			I	K		S									R	N	K		A				R	Y	H		
**HSS**																																						
WT-c21	0.69 (0.04)															K	R		D	M	K		N	K		R		Q	G	R	Y	H						
WT-c22	0.41 (0.03)															K	R		D	M	K		N	K		R		Q	G	R	Y							
WT-c41	0.6 (0.03)																		D	M	K		N	K		R							R	Y				
WT-c42	0.5 (0.04)															K	R		D	M	K		N	K		R								R	Y			
WT-c43	0.49 (0.06)															K	R		D	K	K		N	K		R		Q	G	R	Y							
WT-c44	0.44 (0.03)															K	T			R	K		N	K		R		Q	G	R	Y							
I358F-c21	0.75 (0.06)					F										K	T			R	K		N	K									Q	G	R	Y		
I358F-c22	0.61 (0.04)					F										K	T			R	K		N	K		A	V					Q	G	R	Y			
I358F-c41	0.6 (0.07)					F										K	T			R	K		N	K		A		R		R	Y							
I358F-c42	0.44 (0.05)					F										K	T			R	K		N	K		R		R		R	Y							
I358F-c43	0.73 (0.07)					F										K	T			R	K		N	K		K							R	Y				
I358F-c44	0.5 (0.05)					F														R	K		N	K		A		R	G	R	Y							
I358F-c45	0.59 (0.06)					F										K	T			R	K		N	K		V		R	G	R	Y							
BA1-c21	1.2 (0.12)	D					L	P	F					N	K	T				R	K		N	K		A		G		R	Y	H						
BA1-c41	1.7 (0.16)	D					L	P	F					N	K		S	D	M	K		N	K		R								R	Y	H			
BA1-c42	1.67 (0.17)	D					L	P	F					N	K		S	D	M	K		N	K		R									R	Y			
BA1-c43	1.68 (0.17)	D					L	P	F					N	K	A			R	K		N	K		R									R	Y	H		
BA1-c44	0.96 (0.1)	D					L	P	F					N	K		S	D	M	K		N	K	I	R		Q	G	R	Y								
BA2-c21	1.1 (0.08)	D					F	P	F	A			N	S	N	K	R		D	M	K		N	K		R		Q	G	R	Y	H						
BA2-c41	0.86 (0.06)	D					F	P	F	A			N	S	N	K	R		D	K	K		N	K		R								R	Y	H		
BA2-c42	1.3 (0.16)	D					F	P	F	A			N	S	N	K	R		D	M	K		N	K		A		G	G	R	Y	H						
BA2-c43	1.1 (0.14)	D					F	P	F	A			N	S	N	K	R		D	K	K		N	K		R		Q	G	R	Y	H						

Binding affinities were calculated from at least 3 independent replicates using GraphPad v.10 and one site specific binding model (see Fig. 4 and the related source file). In parenthesis are $\pm$ 95% CI values. Mutant residues shown only for given position are relative to WT (first seq. with residues shown), as determined by Sanger sequencing of the selected clones. For HSS residues in gray are those in the stability determining region, which were not mutated.

Supporting information part PS2 – Analysis of error prone libraries for high stringency selections

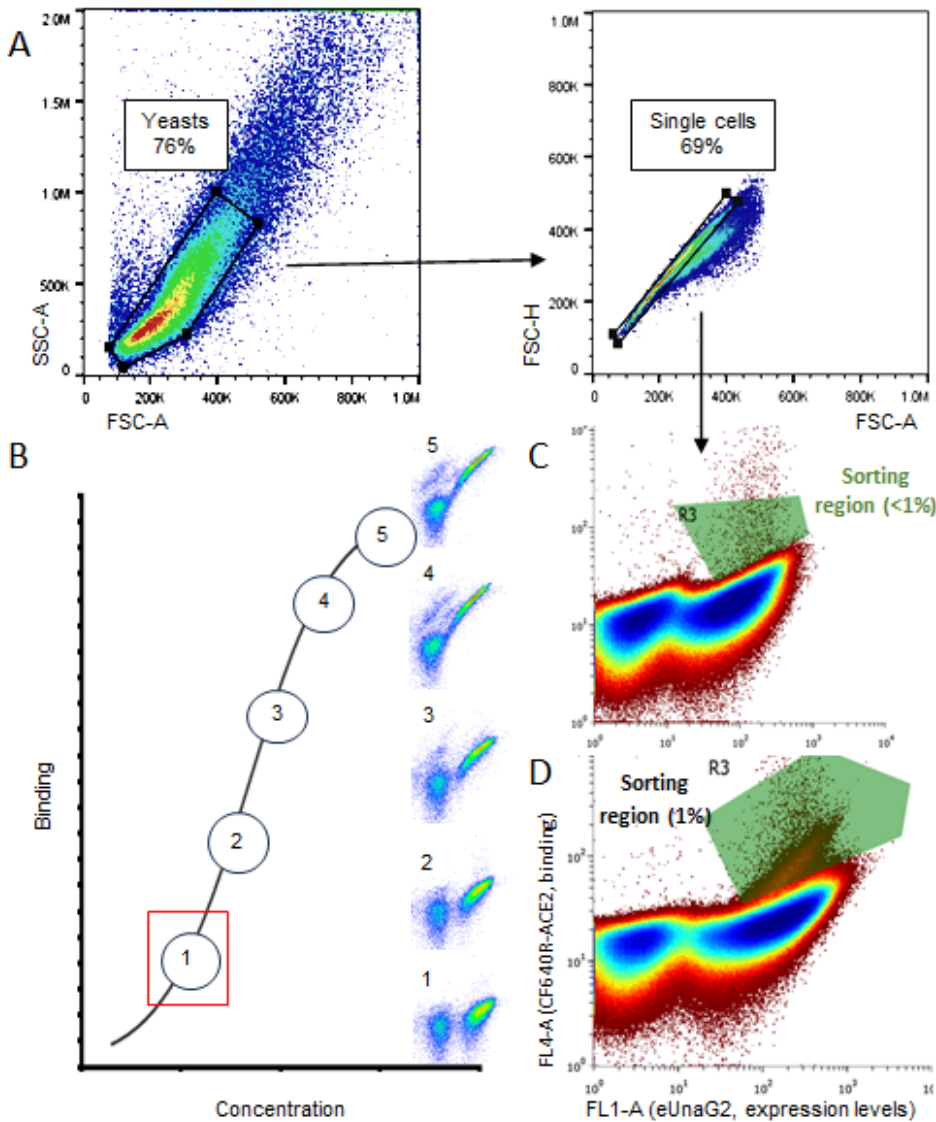

Fig. S1 – Gating and selection strategies for *in vitro* evolution of the SARS-CoV-2 RBD domain. (A) Gating strategy used during FACS sorting. Yeast cells were selected based on their forward and side scatter area parameters (FSC-A and SSC-A) and subsequently, single cells were gated using a diagonal plot of FCS-A versus height (FCS-H). (B) Sorting under different ACE-2 concentrations. The library was incubated with a range of ACE2 concentrations to identify the minimal concentration that promotes elevated signal (inset 1-3). Under these conditions, clones with stronger binding affinity had the highest competitive/signal advantage over the parental population. For LSS higher concentration conditions were used (insets 4–5). (C) Stepwise sorting strategy for HSS to enhance discrimination between improved clones and the parental population. Initially, below 1% of top signal cells were sorted in HSS. These selected cells were then cultured, expressed and subjected to a second round of sorting for higher enrichment of the enhanced binding population (D).

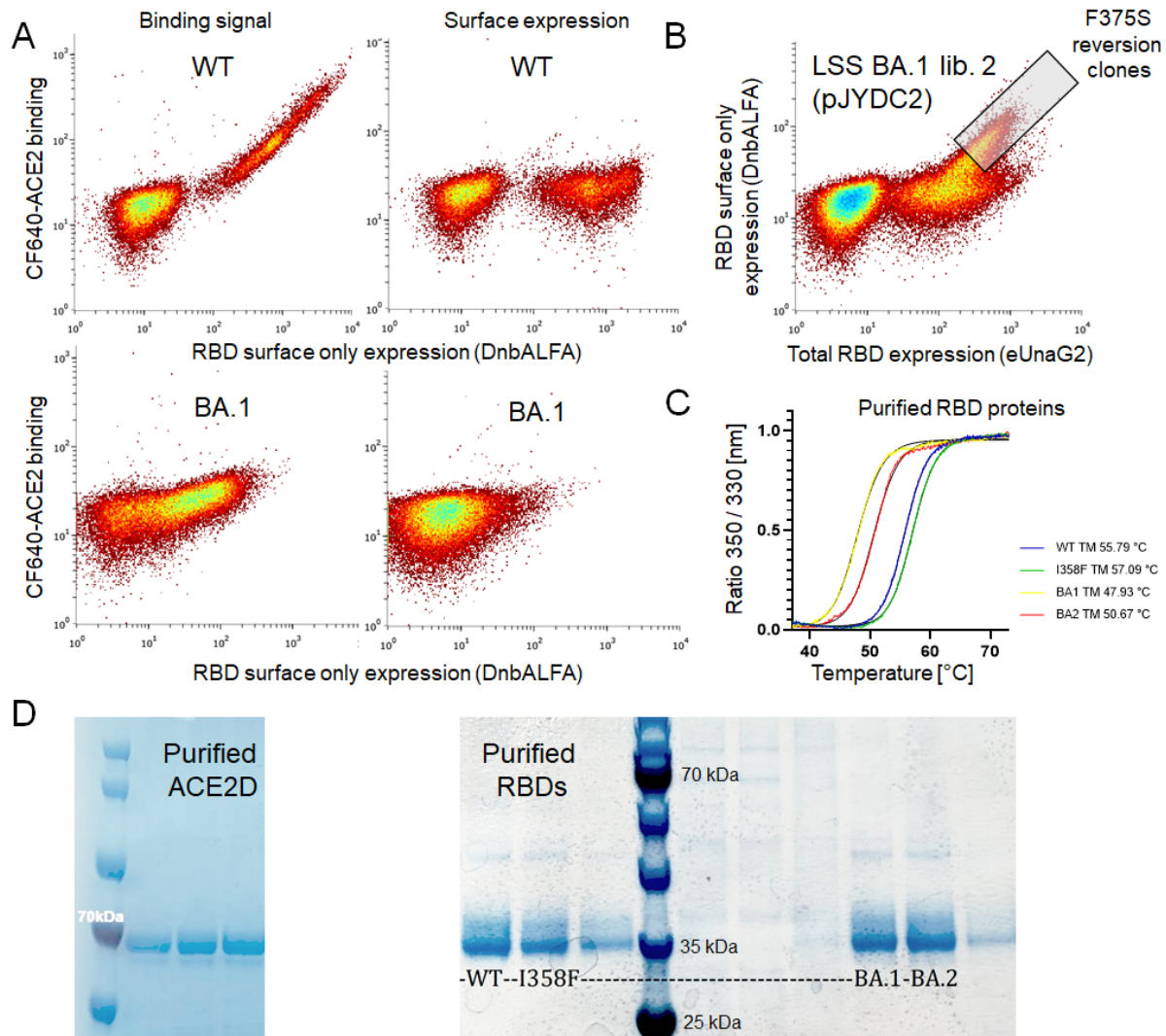

Fig. S2 – Yeast surface display and stability analysis of purified proteins. (A) Flow cytometry dot plot showing strong ACE2 binding and robust surface expression for the wild-type (WT) RBD, in contrast to markedly reduced binding and surface expression observed for the BA.1 RBD. (B) A dominant population displaying a reverted phenotype, relative to BA.1 in panel A, dominated in the population as early as the second round of selection. Surface versus total expression was assessed using the pJYDC2 plasmid. Sequencing identified the F375S reversion as responsible for the restored phenotype. (C) Tycho NT.6 (NanoTemper) thermal unfolding profiles of purified WT, WT+I358F, BA.1, and BA.2 RBDs, demonstrating substantial destabilization of both Omicron lineages relative to WT. (D) SDS-PAGE analysis of purified ACE2D and RBDs proteins with marker (BlueRay Prestained Protein Marker). Uncropped gels are provided in file: Source data for Fig. S2 after Supplementary Data Tables and Figures (this document).

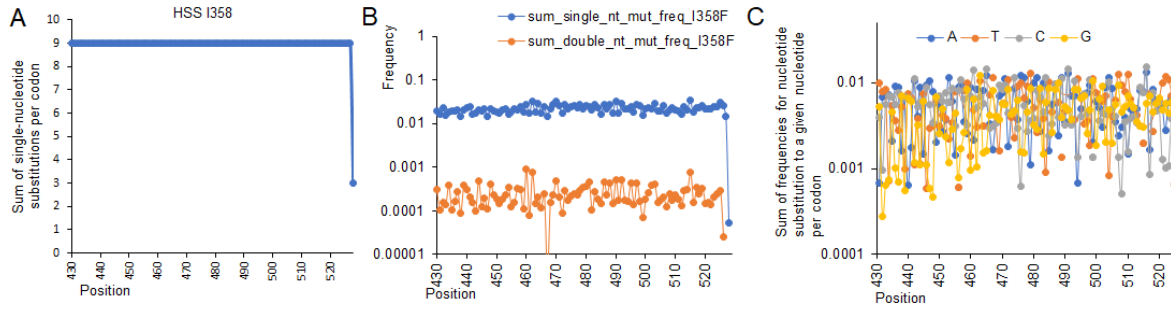

Fig. S3 – WT+I358F library quality assessment. (A) The number of observed single nucleotide substitutions per codon along the RBM, with 9 representing all mutations in all three positions. (B) Frequencies for all single- and double-nucleotide substitutions per codon, showing minimal positional deviations. (C) Sum of single-nucleotide mutation frequencies per mutant nucleotide and codon.

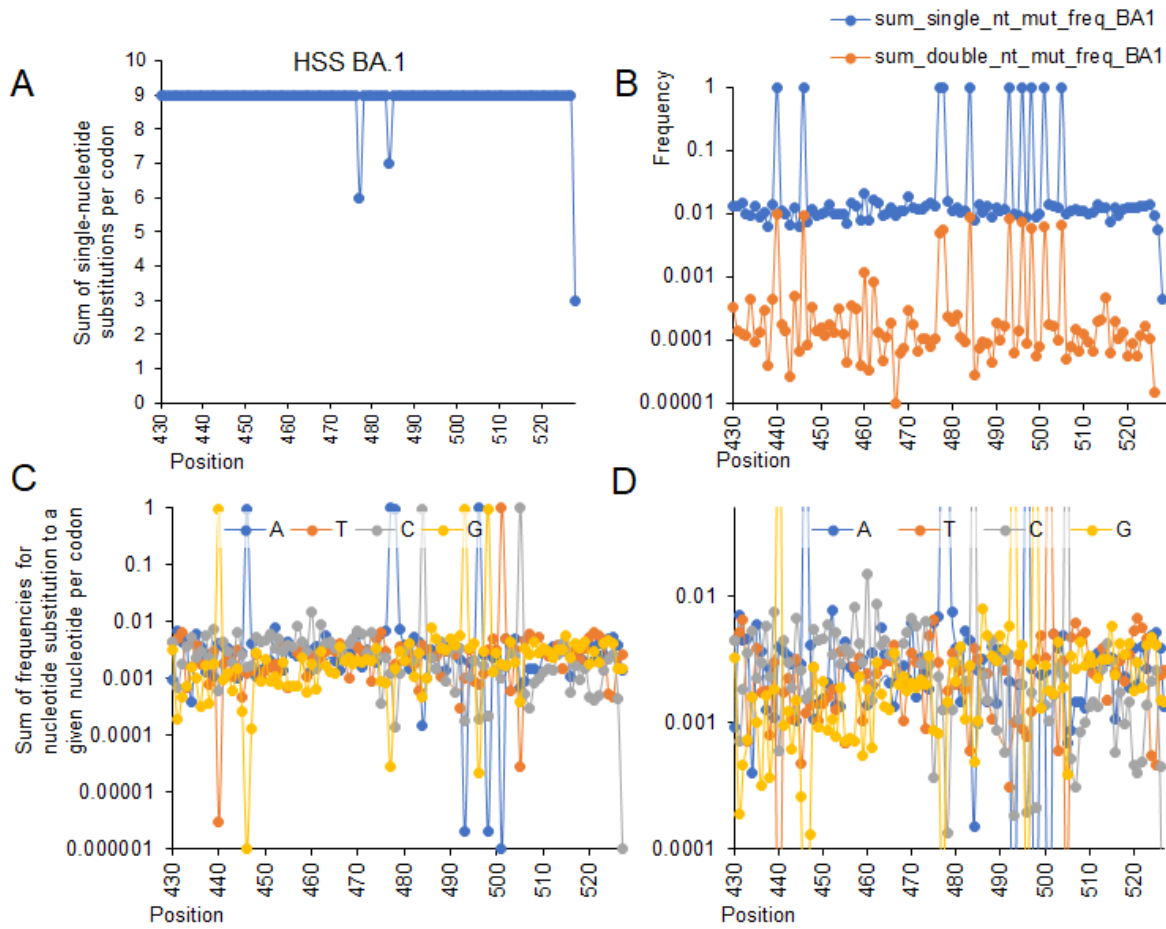

Fig. S4 – BA.1 library quality assessment. (A) The number of observed single nucleotide substitutions per codon along the RBM, with 9 representing all mutations in all three positions. (B) Frequencies for all single- and double-nucleotide substitutions per codon, showing minimal positional deviations. Note that the high frequencies in certain positions are of the BA.1 mutations. WT sequence was used as a reference. (C) Sum of single-nucleotide mutation frequencies per mutant nucleotide and codon with WT reference sequence. (D) Sum of single-nucleotide mutation frequencies per mutant nucleotide and codon – detail for frequencies from 0.001 to 0.01.

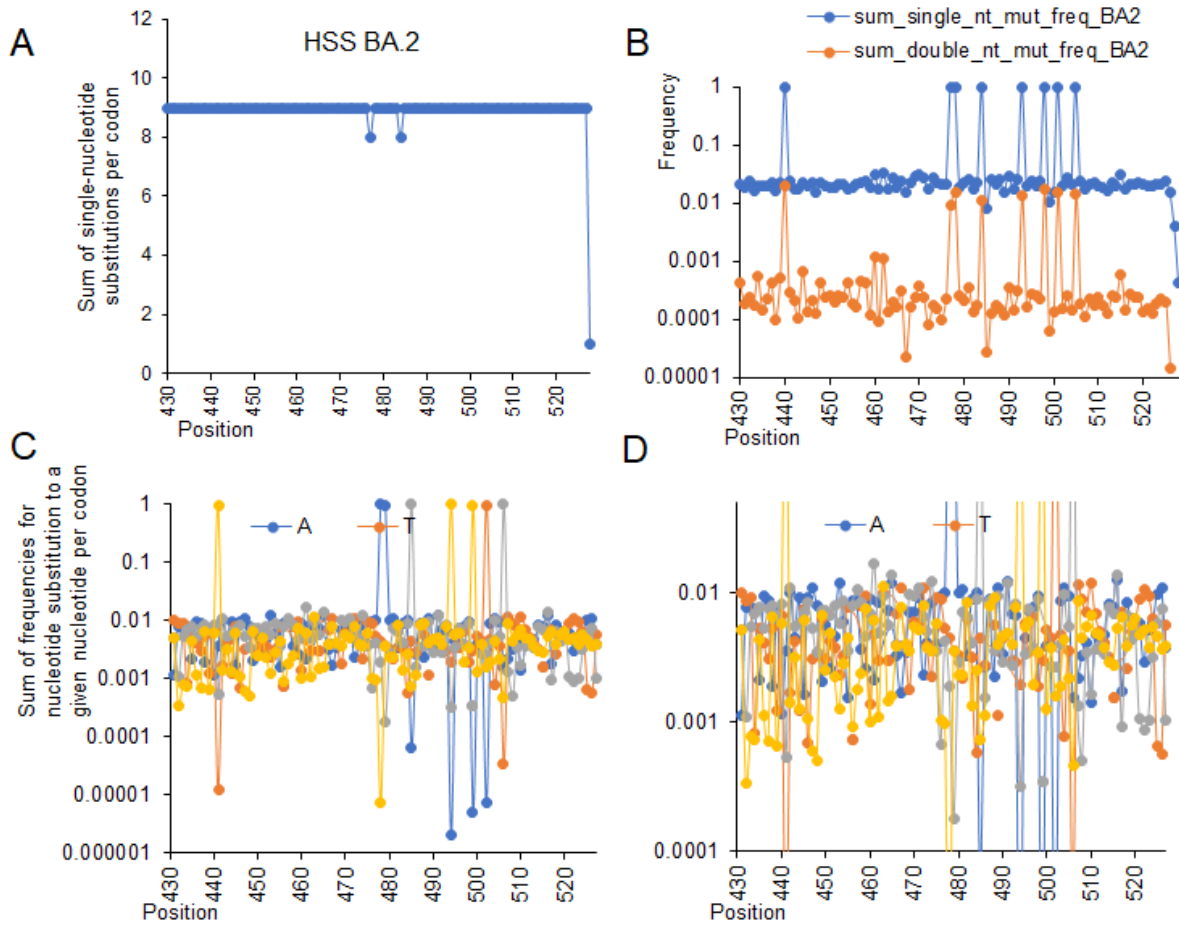

Fig. S5 – BA.2 library quality assessment. (A) The number of observed single nucleotide substitutions per codon along the RBM, with 9 representing all mutations in all three positions. (B) Frequencies for all single- and double-nucleotide substitutions per codon, showing minimal positional deviations. Note that the high frequencies in certain positions are of the BA.2 mutations. WT sequence was used as reference. (C) Sum of single-nucleotide mutation frequencies per mutant nucleotide and codon with WT reference sequence. (D) Sum of single-nucleotide mutation frequencies per mutant nucleotide and codon – detail for frequencies from 0.001 to 0.01.

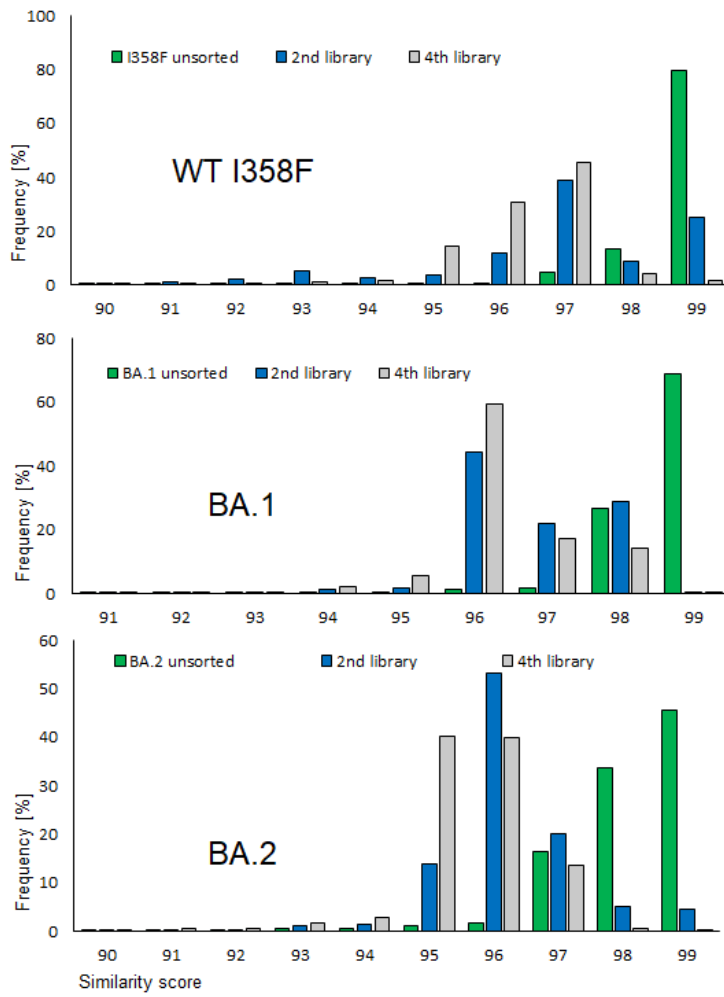

Fig. S6 – Comparison of similarity scores for individual sequencing reads in HSS libraries. Merged paired-end reads into a single sequence with WT, BA.1 and BA.2 sequences as the reference for the unsorted initial library. Populations after sorting in the 2nd and 4th rounds of the *in vitro* evolution are shown. The sequenced regions and corresponding merged paired-end read length for individual libraries differ, and therefore, similarity scores cannot be compared across libraries. More detailed distributions are shown in Fig. S7.

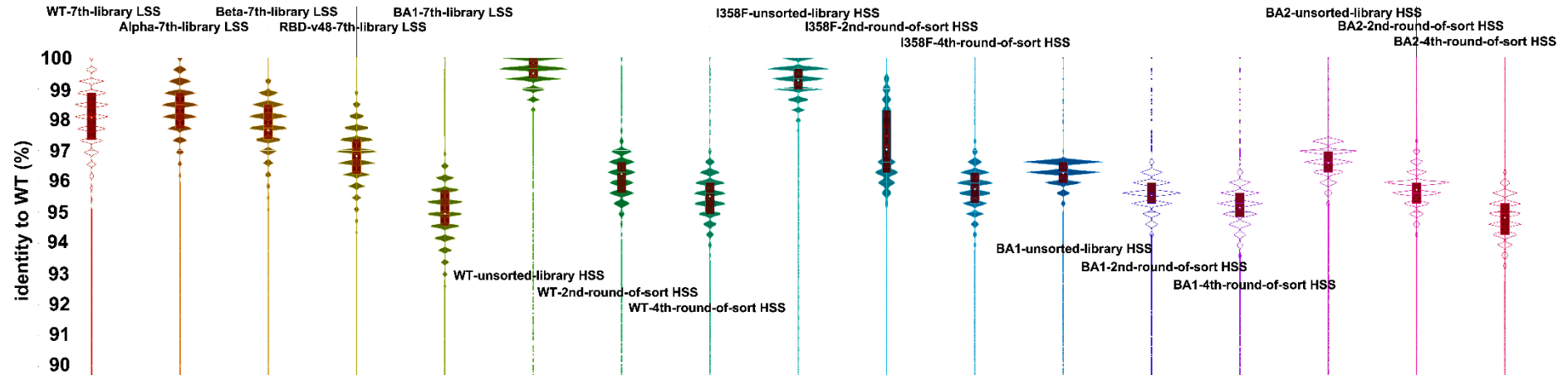

Fig. S7 – Distribution of nucleotide sequence identities among libraries. Sequences were aligned to Wuhan-Hu-1 sequence reference. The individual violin plots combined with Whisker's box plots show detailed distribution of sequence identities in each sample dataset. Notably, a white spot in the middle represents mean value. The bottom part of the violin with lower bounds has been cut away for clarity, considered uninformative. The figure shows decreasing sequence similarity with additional PCR-mutagenesis steps followed by functional selection for ACE2 receptor binding. The mutation load in 7th LSS BA.1 library closely matches with the one observed for 4th HSS BA.1. The sequence identities to the Wuhan S protein gene at the DNA-level of the RBM amplicons were obtained by BLASTN v2.17.0+ and the violin plots were rendered using ggViolin function from the ggiraphExtra v0.3.0 R package.

Shoshany *et al.* (2026) Stringent Selection Drives Convergence Toward Omicron-like SARS-CoV-2 Receptor-Binding Motifs

WT	1	2	3	4	5	6	7	8	9	10	11	12	13	14	15	16	17	18	19	20	21	22	23	24	25	26	27	28	29	30	31	32	33	34	35	36	37	38	39	40	41	42	43	44	45	46	47	48	49	50	51	52	53	54	55	56	57	58	59	60	61	62	63	64	65	66	67	68	69	70	71	72	73	74	75	76	77	78	79	80	81	82	83	84	85	86	87	88	89	90	91	92	93	94	95	96	97	98	99	100	101	102	103	104	105	106	107	108	109	110	111	112	113	114	115	116	117	118	119	120	121	122	123	124	125	126	127	128	129	130	131	132	133	134	135	136	137	138	139	140	141	142	143	144	145	146	147	148	149	150	151	152	153	154	155	156	157	158	159	160	161	162	163	164	165	166	167	168	169	170	171	172	173	174	175	176	177	178	179	180	181	182	183	184	185	186	187	188	189	190	191	192	193	194	195	196	197	198	199	200	201	202	203	204	205	206	207	208	209	210	211	212	213	214	215	216	217	218	219	220	221	222	223	224	225	226	227	228	229	230	231	232	233	234	235	236	237	238	239	240	241	242	243	244	245	246	247	248	249	250	251	252	253	254	255	256	257	258	259	260	261	262	263	264	265	266	267	268	269	270	271	272	273	274	275	276	277	278	279	280	281	282	283	284	285	286	287	288	289	290	291	292	293	294	295	296	297	298	299	300	301	302	303	304	305	306	307	308	309	310	311	312	313	314	315	316	317	318	319	320	321	322	323	324	325	326	327	328	329	330	331	332	333	334	335	336	337	338	339	340	341	342	343	344	345	346	347	348	349	350	351	352	353	354	355	356	357	358	359	360	361	362	363	364	365	366	367	368	369	370	371	372	373	374	375	376	377	378	379	380	381	382	383	384	385	386	387	388	389	390	391	392	393	394	395	396	397	398	399	400	401	402	403	404	405	406	407	408	409	410	411	412	413	414	415	416	417	418	419	420	421	422	423	424	425	426	427	428	429	430	431	432	433	434	435	436	437	438	439	440	441	442	443	444	445	446	447	448	449	450	451	452	453	454	455	456	457	458	459	460	461	462	463	464	465	466	467	468	469	470	471	472	473	474	475	476	477	478	479	480	481	482	483	484	485	486	487	488	489	490	491	492	493	494	495	496	497	498	499	500	501	502	503	504	505	506	507	508	509	510	511	512	513	514	515	516	517	518	519	520	521	522	523	524	

Fig. S8 – Translated Sanger sequencing results for selected clones for WT LSS libraries 1 to 7. Residues identical to the WT sequence are shown in blue, whereas mutated amino acids are indicated in white.

Shoshany *et al.* (2026) Stringent Selection Drives Convergence Toward Omicron-like SARS-CoV-2 Receptor-Binding Motifs[illegible]

Fig. S9 – Translated Sanger sequencing results for selected clones for Alpha LSS libraries 1 to 7. Residues identical to the WT sequence are shown in blue, whereas mutated amino acids are indicated in white. Characteristic residue for the Alpha variant relative to the WT RBD is highlighted in yellow (N501Y mutation).

Shoshany *et al.* (2026) Stringent Selection Drives Convergence Toward Omicron-like SARS-CoV-2 Receptor-Binding Motifs

Index	Gene	Accession	Length	GC Content	GC Skew	GC Bias	GC Bias2	GC Bias3	GC Bias4	GC Bias5	GC Bias6	GC Bias7	GC Bias8	GC Bias9	GC Bias10	GC Bias11	GC Bias12	GC Bias13	GC Bias14	GC Bias15	GC Bias16	GC Bias17	GC Bias18	GC Bias19	GC Bias20	GC Bias21	GC Bias22	GC Bias23	GC Bias24	GC Bias25	GC Bias26	GC Bias27	GC Bias28	GC Bias29	GC Bias30	GC Bias31	GC Bias32	GC Bias33	GC Bias34	GC Bias35	GC Bias36	GC Bias37	GC Bias38	GC Bias39	GC Bias40	GC Bias41	GC Bias42	GC Bias43	GC Bias44	GC Bias45	GC Bias46	GC Bias47	GC Bias48	GC Bias49	GC Bias50	GC Bias51	GC Bias52	GC Bias53	GC Bias54	GC Bias55	GC Bias56	GC Bias57	GC Bias58	GC Bias59	GC Bias60	GC Bias61	GC Bias62	GC Bias63	GC Bias64	GC Bias65	GC Bias66	GC Bias67	GC Bias68	GC Bias69	GC Bias70	GC Bias71	GC Bias72	GC Bias73	GC Bias74	GC Bias75	GC Bias76	GC Bias77	GC Bias78	GC Bias79	GC Bias80	GC Bias81	GC Bias82	GC Bias83	GC Bias84	GC Bias85	GC Bias86	GC Bias87	GC Bias88	GC Bias89	GC Bias90	GC Bias91	GC Bias92	GC Bias93	GC Bias94	GC Bias95	GC Bias96	GC Bias97	GC Bias98	GC Bias99	GC Bias100	GC Bias101	GC Bias102	GC Bias103	GC Bias104	GC Bias105	GC Bias106	GC Bias107	GC Bias108	GC Bias109	GC Bias110	GC Bias111	GC Bias112	GC Bias113	GC Bias114	GC Bias115	GC Bias116	GC Bias117	GC Bias118	GC Bias119	GC Bias120	GC Bias121	GC Bias122	GC Bias123	GC Bias124	GC Bias125	GC Bias126	GC Bias127	GC Bias128	GC Bias129	GC Bias130	GC Bias131	GC Bias132	GC Bias133	GC Bias134	GC Bias135	GC Bias136	GC Bias137	GC Bias138	GC Bias139	GC Bias140	GC Bias141	GC Bias142	GC Bias143	GC Bias144	GC Bias145	GC Bias146	GC Bias147	GC Bias148	GC Bias149	GC Bias150	GC Bias151	GC Bias152	GC Bias153	GC Bias154	GC Bias155	GC Bias156	GC Bias157	GC Bias158	GC Bias159	GC Bias160	GC Bias161	GC Bias162	GC Bias163	GC Bias164	GC Bias165	GC Bias166	GC Bias167	GC Bias168	GC Bias169	GC Bias170	GC Bias171	GC Bias172	GC Bias173	GC Bias174	GC Bias175	GC Bias176	GC Bias177	GC Bias178	GC Bias179	GC Bias180	GC Bias181	GC Bias182	GC Bias183	GC Bias184	GC Bias185	GC Bias186	GC Bias187	GC Bias188	GC Bias189	GC Bias190	GC Bias191	GC Bias192	GC Bias193	GC Bias194	GC Bias195	GC Bias196	GC Bias197	GC Bias198	GC Bias199	GC Bias200	GC Bias201	GC Bias202	GC Bias203	GC Bias204	GC Bias205	GC Bias206	GC Bias207	GC Bias208	GC Bias209	GC Bias210	GC Bias211	GC Bias212	GC Bias213	GC Bias214	GC Bias215	GC Bias216	GC Bias217	GC Bias218	GC Bias219	GC Bias220	GC Bias221	GC Bias222	GC Bias223	GC Bias224	GC Bias225	GC Bias226	GC Bias227	GC Bias228	GC Bias229	GC Bias230	GC Bias231	GC Bias232	GC Bias233	GC Bias234	GC Bias235	GC Bias236	GC Bias237	GC Bias238	GC Bias239	GC Bias240	GC Bias241	GC Bias242	GC Bias243	GC Bias244	GC Bias245	GC Bias246	GC Bias247	GC Bias248	GC Bias249	GC Bias250	GC Bias251	GC Bias252	GC Bias253	GC Bias254	GC Bias255	GC Bias256	GC Bias257	GC Bias258	GC Bias259	GC Bias260	GC Bias261	GC Bias262	GC Bias263	GC Bias264	GC Bias265	GC Bias266	GC Bias267	GC Bias268	GC Bias269	GC Bias270	GC Bias271	GC Bias272	GC Bias273	GC Bias274	GC Bias275	GC Bias276	GC Bias277	GC Bias278	GC Bias279	GC Bias280	GC Bias281	GC Bias282	GC Bias283	GC Bias284	GC Bias285	GC Bias286	GC Bias287	GC Bias288	GC Bias289	GC Bias290	GC Bias291	GC Bias292	GC Bias293	GC Bias294	GC Bias295	GC Bias296	GC Bias297	GC Bias298	GC Bias299	GC Bias300	GC Bias301	GC Bias302	GC Bias303	GC Bias304	GC Bias305	GC Bias306	GC Bias307	GC Bias308	GC Bias309	GC Bias310	GC Bias311	GC Bias312	GC Bias313	GC Bias314	GC Bias315	GC Bias316	GC Bias317	GC Bias318	GC Bias319	GC Bias320	GC Bias321	GC Bias322	GC Bias323	GC Bias324	GC Bias325	GC Bias326	GC Bias327	GC Bias328	GC Bias329	GC Bias330	GC Bias331	GC Bias332	GC Bias333	GC Bias334	GC Bias335	GC Bias336	GC Bias337	GC Bias338	GC Bias339	GC Bias340	GC Bias341	GC Bias342	GC Bias343	GC Bias344	GC Bias345	GC Bias346	GC Bias347	GC Bias348	GC Bias349	GC Bias350	GC Bias351	GC Bias352	GC Bias353	GC Bias354	GC Bias355	GC Bias356	GC Bias357	GC Bias358	GC Bias359	GC Bias360	GC Bias361	GC Bias362	GC Bias363	GC Bias364	GC Bias365	GC Bias366	GC Bias367	GC Bias368	GC Bias369	GC Bias370	GC Bias371	GC Bias372	GC Bias373	GC Bias374	GC Bias375	GC Bias376	GC Bias377	GC Bias378	GC Bias379	GC Bias380	GC Bias381	GC Bias382	GC Bias383	GC Bias384	GC Bias385	GC Bias386	GC Bias387	GC Bias388	GC Bias389	GC Bias390	GC Bias391	GC Bias392	GC Bias393	GC Bias394	GC Bias395	GC Bias396	GC Bias397	GC Bias398	GC Bias399	GC Bias400	GC Bias401	GC Bias402	GC Bias403	GC Bias404	GC Bias405	GC Bias406	GC Bias407	GC Bias408	GC Bias409	GC Bias410	GC Bias411	GC Bias412	GC Bias413	GC Bias414	GC Bias415	GC

Fig. S10 – Translated Sanger sequencing results for selected clones for Beta LSS libraries 1 to 7. Residues identical to the WT sequence are shown in blue, whereas mutated amino acids are indicated in white. Characteristic Beta variant residues relative to the WT RBD are highlighted in yellow (K417N, E484K, N501Y mutations).

Shoshany *et al.* (2026) Stringent Selection Drives Convergence Toward Omicron-like SARS-CoV-2 Receptor-Binding Motifs

RBD-v48 library	clone	WT
		335 337 338 339 340 341 342 343 344 345 346 347 348 349 350 351 352 353 354 355 356 357 358 359 360 361 362 363 364 365 366 367 368 369 370 371 372 373 374 375 376 377 378 379 380 381 382 383 384 385 386 387 388 389 390 391 392 393 394 395 396 397 398 399 400 401 402 403 404 405 406 407 408 409 410 411 412 413 414 415 416 417 418 419 420 421 422 423 424 425 426 427 428 429 430 431 432 433 434 435 436 437 438 439 440 441 442 443 444 445 446 447 448 449 450 451 452 453 454 455 456 457 458 459 460 461 462 463 464 465 466 467 468 469 470 471 472 473 474 475 476 477 478 479 480 481 482 483 484 485 486 487 488 489 490 491 492 493 494 495 496 497 498 499 500 501 502 503 504 505 506 507 508 509 510 511 512 513 514 515 516 517 518 519 520 521 522 523 524 525 526 527 528

Fig. S11 – Translated Sanger sequencing results for selected clones for RBD-v48 LSS libraries 1 to 7. Residues identical to the WT sequence are shown in blue, whereas mutated amino acids are indicated in white. Characteristic RBD-v48 variant residues relative to the WT RBD are highlighted in yellow (K417N, E484K, Q498R, N501Y mutations).

Shoshany *et al.* (2026) Stringent Selection Drives Convergence Toward Omicron-like SARS-CoV-2 Receptor-Binding Motifs

BA1

unsorted

1

2

3

4

5

6

7

8

9

10

11

12

13

14

15

16

17

18

19

20

21

22

23

24

25

26

27

28

29

30

31

32

33

34

35

36

37

38

39

40

41

42

43

44

45

46

47

48

49

50

51

CPDEVNATRFASVYVWNNKRKISNCVDYSVLYLAPFTFKYGVSPSTKLNLCFTNNVADS FVIRGDEVRIQAPGGTGADYNYLKLPDFDTGCVIAWNSLDSKVGNNYNYLYLFRKSNLKPFFERDITEIYQAGNKPNGVAGNCFYFPLSSPTTGVGQPPRRVVLSFELLHAPATVCGPK

CPDEVNATRFASVYVWNNKRKISNCVDYSVLYLAPFTFKYGVSPSTKLNLCFTNNVADS FVIRGDEVRIQAPGGTGADYNYLKLPDFDTGCVIAWNSLDSKVGNNYLYLFRKSNLKPFFERDITEIYQAGNKPNGVAGNCFYFPLSSPTTGVGQPPRRVVLSFELLHAPATVCGPK

CPDEVNATRFASVYVWNNKRKISNCVDYSVLYLAPFTFKYGVSPSTKLNLCFTNNVADS FVIRGDEVRIQAPGGTGADYNYLKLPDFDTGCVIAWNSLDSKVGNNYLYLFRKSNLKPFFERDITEIYQAGNKPNGVAGN

Shoshany *et al.* (2026) Stringent Selection Drives Convergence Toward Omicron-like SARS-CoV-2 Receptor-Binding Motifs

BA.1 library	residue	WT	BA.1
36		C	P
37		P	E
38		E	V
39		V	N
40		N	A
41		A	K
42		K	R
43		R	K
44		K	R
45		R	K
46		K	R
47		R	K
48		K	R
49		R	K
50		K	R
51		R	K
52		K	R
53		R	K
54		K	R
55		R	K
56		K	R
57		R	K
58		K	R
59		R	K
60		K	R
61		R	K
62		K	R
63		R	K
64		K	R
65		R	K
66		K	R
67		R	K
68		K	R
69		R	K
70		K	R
71		R	K
72		K	R
73		R	K
74		K	R
75		R	K
76		K	R
77		R	K
78		K	R
79		R	K
80		K	R
81		R	K
82		K	R
83		R	K
84		K	R
85		R	K
86		K	R
87		R	K
88		K	R
89		R	K
90		K	R
91		R	K
92		K	R
93		R	K
94		K	R
95		R	K
96		K	R
97		R	K
98		K	R
99		R	K
100		K	R
101		R	K
102		K	R
103		R	K
104		K	R
105		R	K
106		K	R
107		R	K
108		K	R
109		R	K
110		K	R
111		R	K
112		K	R
113		R	K
114		K	R
115		R	K
116		K	R
117		R	K
118		K	R
119		R	K
120		K	R
121		R	K
122		K	R
123		R	K
124		K	R
125		R	K
126		K	R
127		R	K
128		K	R
129		R	K
130		K	R
131		R	K
132		K	R
133		R	K
134		K	R
135		R	K
136		K	R
137		R	K
138		K	R
139		R	K
140		K	R
141		R	K
142		K	R
143		R	K
144		K	R
145		R	K
146		K	R
147		R	K
148		K	R
149		R	K
150		K	R
151		R	K
152		K	R
153		R	K
154		K	R
155		R	K
156		K	R
157		R	K
158		K	R
159		R	K
160		K	R
161		R	K
162		K	R
163		R	K
164		K	R
165		R	K
166		K	R
167		R	K
168		K	R
169		R	K
170		K	R
171		R	K
172		K	R
173		R	K
174		K	R
175		R	K
176		K	R
177		R	K
178		K	R
179		R	K
180		K	R
181		R	K
182		K	R
183		R	K
184		K	R
185		R	K
186		K	R
187		R	K
188		K	R
189		R	K
190		K	R
191		R	K
192		K	R
193		R	K
194		K	R
195		R	K
196		K	R
197		R	K
198		K	R
199		R	K
200		K	R
201		R	K
202		K	R
203		R	K
204		K	R
205		R	K
206		K	R
207		R	K
208		K	R
209		R	K
210		K	R
211		R	K
212		K	R
213		R	K
214		K	R
215		R	K
216		K	R
217		R	K
218		K	R
219		R	K
220		K	R
221		R	K
222		K	R
223		R	K
224		K	R
225		R	K
226		K	R
227		R	K
228		K	R
229		R	K
230		K	R
231		R	K
232		K	R
233		R	K
234		K	R
235		R	K
236		K	R
237		R	K
238		K	R
239		R	K
240		K	R
241		R	K
242		K	R
243		R	K
244		K	R
245		R	K
246		K	R
247		R	K
248		K	R
249		R	K
250		K	R
251		R	K
252		K	R
253		R	K
254		K	R
255		R	K
256		K	R
257		R	K
258		K	R
259		R	K
260		K	R
261		R	K
262		K	R
263		R	K
264		K	R
265		R	K
266		K	R
267		R	K
268		K	R
269		R	K
270		K	R
271		R	K
272		K	R
273		R	K
274		K	R
275		R	K
276		K	R
277		R	K
278		K	R
279		R	K
280		K	R
281		R	K
282		K	R
283		R	K
284		K	R
285		R	K
286		K	R
287		R	K
288		K	R
289		R	K
290		K	R
291		R	K
292		K	R
293		R	K
294		K	R
295		R	K
296		K	R
297		R	K
298		K	R
299		R	K
300		K	R
301		R	K
302		K	R
303		R	K
304		K	R
305		R	K
306		K	R
307		R	K
308		K	R
309		R	K
310		K	R
311		R	K
312		K	R
313		R	K
314		K	R
315		R	K
316		K	R
317		R	K
318		K	R
319		R	K
320		K	R
321		R	K
322		K	R
323		R	K
324		K	R
325		R	K
326		K	R
327		R	K
328		K	R
329		R	K
330		K	R
331		R	K
332		K	R
333		R	K
334		K	R
335		R	K
336		K	R
337		R	K
338		K	R
339		R	K
340		K	R
341		R	K
342		K	R
343		R	K
344		K	R
345		R	K
346		K	R
347		R	K
348		K	R
349		R	K
350		K	R
351		R	K
352		K	R
353		R	K
354		K	R
355		R	K
356		K	R
357		R	K
358		K	R
359		R	K
360		K	R
361		R	K
362		K	R
363		R	K
364		K	R
365		R	K
366		K	R
367		R	K
368		K	R
369		R	K
370		K	R
371		R	K
372		K	R
373		R	K
374		K	R
375		R	K
376		K	R
377		R	K
378		K	R
379		R	K
380		K	R
381		R	K
382		K	R
383		R	K
384		K	R
385		R	K
386		K	R
387		R	K
388		K	R
389		R	K
390		K	R
391		R	K
392		K	R
393		R	K
394		K	R
395		R	K
396		K	R
397		R	K
398		K	R
399		R	K
400		K	R
401		R	K
402		K	R
403		R	K
404		K	R
405		R	K
406		K	R
407		R	K
408		K	R
409		R	K
410		K	R
411		R	K
412		K	R
413		R	K
414		K	R
415		R	K
416		K	R
417		R	K
418		K	R
419		R	K
420		K	R
421		R	K
422		K	R
423		R	K
424		K	R
425		R	K
426		K	R
427		R	K
428		K	R
429		R	K
430		K	R
431		R	K
432		K	R
433		R	K
434		K	R
435		R	K
436		K	R
437		R	K
438		K	R
439		R	K
440		K	R
441		R	K
442		K	R
443		R	K
444		K	R
445		R	K
446		K	R
447		R	K
448		K	R
449		R	K
450		K	R
451		R	K
452		K	R
453		R	K
454		K	R
455		R	K
456		K	R
457		R	K
458		K	R
459		R	K
460		K	R
461		R	K
462		K	R
463		R	K
464		K	R
465		R	K
466		K	R
467		R	K
468		K	R
469		R	K
470		K	R
471		R	K
472		K	R
473		R	K
474		K	R
475	</		

Shoshany *et al.* (2026) Stringent Selection Drives Convergence Toward Omicron-like SARS-CoV-2 Receptor-Binding Motifs

Lib	WT	1	2	3	4	5	6	7	8	9	10	11	12	13	14	15	16	17	18	19	20	21	22	23	24	25	26	27	28	29	30	31	32	33	34	35	36	37	38	39	40	41	42	43	44	45	46	47	48	49	50	51	52	53	54	55	56	57	58	59	60	61	62	63	64	65	66	67	68	69	70	71	72	73	74	75	76	77	78	79	80	81	82	83	84	85	86	87	88	89	90	91	92	93	94	95	96	97	98	99	100
WT	T	G	C	C	A	A	N	S	N	L	D	S	K	V	G	G	N	N	Y	R	L	F	R	K	S	K	L	K	P	F	F	E	R	D	I	S	T	E	Y	A	G	A	N	K	P	C	N	G	V	R	G	N	C	V	F	P	L	S	G	Y	G	R	P	P	G	V	G	Y	Q	P	P	R	V	V	L	S	F	E	L	L	H	A	P	A	T	V	G														
WT3	T	G	C	C	A	A	N	S	N	L	D	S	K	V	G	G	N	N	D	M	M	Y	R	L	F	R	K	S	K	L	K	P	F	F	E	R	D	I	S	T	E	Y	A	G	A	N	K	P	C	N	G	V	R	G	N	C	V	F	P	L	S	G	Y	G	R	P	P	G	V	G	Y	Q	P	P	R	V	V	L	S	F	E	L	L	H	A	P	A	T	V	G											
WT4	T	G	C	C	A	A	N	S	N	L	D	S	K	V	G	G	N	N	D	M	M	Y	R	L	F	R	K	S	K	L	K	P	F	F	E	R	D	I	S	T	E	Y	A	G	A	N	K	P	C	N	G	V	R	G	N	C	V	F	P	L	S	G	Y	G	R	P	P	G	V	G	Y	Q	P	P	R	V	V	L	S	F	E	L	L	H	A	P	A	T	V	G											
1358F2	T	G	C	C	A	A	N	S	N	L	D	S	K	V	G	G	N	N	D	M	M	Y	R	L	F	R	K	S	K	L	K	P	F	F	E	R	D	I	S	T	E	Y	A	G	A	N	K	P	C	N	G	V	R	G	N	C	V	F	P	L	S	G	Y	G	R	P	P	G	V	G	Y	Q	P	P	R	V	V	L	S	F	E	L	L	H	A	P	A	T	V	G											
1358F3	T	G	C	C	A	A	N	S	N	L	D	S	K	V	G	G	N	N	D	M	M	Y	R	L	F	R	K	S	K	L	K	P	F	F	E	R	D	I	S	T	E	Y	A	G	A	N	K	P	C	N	G	V	R	G	N	C	V	F	P	L	S	G	Y	G	R	P	P	G	V	G	Y	Q	P	P	R	V	V	L	S	F	E	L	L	H	A	P	A	T	V	G											
1358F4	T	G	C	C	A	A	N	S	N	L	D	S	K	V	G	G	N	N	D	M	M	Y	R	L	F	R	K	S	K	L	K	P	F	F	E	R	D	I	S	T	E	Y	A	G	A	N	K	P	C	N	G	V	R	G	N	C	V	F	P	L	S	G	Y	G	R	P	P	G	V	G	Y	Q	P	P	R	V	V	L	S	F	E	L	L	H	A	P	A	T	V	G											
BA12	T	G	C	C	A	A	N	S	N	L	D	S	K	V	G	G	N	N	D	M	M	Y	R	L	F	R	K	S	K	L	K	P	F	F	E	R	D	I	S	T	E	Y	A	G	A	N	K	P	C	N	G	V	R	G	N	C	V	F	P	L	S	G	Y	G	R	P	P	G	V	G	Y	Q	P	P	R	V	V	L	S	F	E	L	L	H	A	P	A	T	V	G											
BA13	T	G	C	C	A	A	N	S	N	L	D	S	K	V	G	G	N	N	D	M	M	Y	R	L	F	R	K	S	K	L	K	P	F	F	E	R	D	I	S	T	E	Y	A	G	A	N	K	P	C	N	G	V	R	G	N	C	V	F	P	L	S	G	Y	G	R	P	P	G	V	G	Y	Q	P	P	R	V	V	L	S	F	E	L	L	H	A	P	A	T	V	G											
BA14	T	G	C	C	A	A	N	S	N	L	D	S	K	V	G	G	N	N	D	M	M	Y	R	L	F	R	K	S	K	L	K	P	F	F	E	R	D	I	S	T	E	Y	A	G	A	N	K	P	C	N	G	V	R	G	N	C	V	F	P	L	S	G	Y	G	R	P	P	G	V	G	Y	Q	P	P	R	V	V	L	S	F	E	L	L	H	A	P	A	T	V	G											
BA22	T	G	C	C	A	A	N	S	N	L	D	S	K	V	G	G	N	N	D	M	M	Y	R	L	F	R	K	S	K	L	K	P	F	F	E	R	D	I	S	T	E	Y	A	G	A	N	K	P	C	N	G	V	R	G	N	C	V	F	P	L	S	G	Y	G	R	P	P	G	V	G	Y	Q	P	P	R	V	V	L	S	F	E	L	L	H	A	P	A	T	V	G											
BA23	T	G	C	C	A	A	N	S	N	L	D	S	K	V	G	G	N	N	D	M	M	Y	R	L	F	R	K	S	K	L	K	P	F	F	E	R	D	I	S	T	E	Y	A	G	A	N	K	P	C	N	G	V	R	G	N	C	V	F	P	L	S	G	Y	G	R	P	P	G	V	G	Y	Q	P	P	R	V	V	L	S	F	E	L	L	H	A	P	A	T	V	G											
BA24	T	G	C	C	A	A	N	S	N	L	D	S	K	V	G	G	N	N	D	M	M	Y	R	L	F	R	K	S	K	L	K	P	F	F	E	R	D	I	S	T	E	Y	A	G	A	N	K	P	C	N	G	V	R	G	N	C	V	F	P	L	S	G	Y	G	R	P	P	G	V	G	Y	Q	P	P	R	V	V	L	S	F	E	L	L	H	A	P	A	T	V	G											

Fig. S13 – Translated Sanger sequencing results for selected HSS libraries clones Residues identical to the WT sequence are shown in blue, whereas mutated amino acids are indicated in white. Residues characteristic for Omicron BA.1 variant are highlighted in yellow (G339D, S371L, S373P, S375F, K417N, N440K, G446S, S477N, T478K, E484A, Q493R, G496S, Q498R, N501Y and Y505H mutations).

Shoshany *et al.* (2026) Stringent Selection Drives Convergence Toward Omicron-like SARS-CoV-2 Receptor-Binding Motifs

res. Number.	430	431	432	433	434	435	436	437	438	439	440	441	442	443	444	445	446	447	448	449	450	451	452	453	454	455	456	457	458	459	460	461	462	463	464	465	466	467	468	469	470	471	472	473	474	475	476	477	478	479	480	481	482	483	484	485	486	487	488	489	490	491	492	493	494	495	496	497	498	499	500	501	502	503	504	505	506	507	508	509	510	511	512	513	514	515	516	517	518	519	520	521	522	523	524	525	526	527	528
WT	T	G	C	V	I	A	W	N	S	N	L	D	S	K	V	G	G	N	Y	L	R	L	R	F	K	S	N	L	K	P	F	E	R	D	I	S	T	E	I	Y	Q	A	G	S	T	P	C	N	G	V	G	F	N	C	Y	F	F	L	Q	S	Y	G	F	A	P	T	N	G	V	G	O	P	Y	R	V	V	L	S	F	E	L	L	H	A	P	A	T	V	C	G	P	K							
WT	T	G	C	V	I	A	W	N	S	N	L	D	S	K	V	G	G	N	Y	L	R	L	R	F	K	S	N	L	K	P	F	E	R	D	I	S	T	E	I	Y	Q	A	G	S	T	P	C	N	G	V	G	F	N	C	Y	F	F	L	Q	S	Y	G	F	A	P	T	N	G	V	G	O	P	Y	R	V	V	L	S	F	E	L	L	H	A	P	A	T	V	C	G	P	K							
Alpha	T	G	C	V	I	A	W	N	S	N	L	D	S	K	V	G	G	N	Y	L	R	L	R	F	K	S	N	L	K	P	F	E	R	D	I	S	T	E	I	Y	Q	A	G	S	T	P	C	N	G	V	G	F	N	C	Y	F	F	L	Q	S	Y	G	F	A	P	T	N	G	V	G	O	P	Y	R	V	V	L	S	F	E	L	L	H	A	P	A	T	V	C	G	P	K							
WT	T	G	C	V	I	A	W	N	S	N	L	D	S	K	V	G	G	N	Y	L	R	L	R	F	K	S	N	L	K	P	F	E	R	D	I	S	T	E	I	Y	Q	A	G	S	T	P	C	N	G	V	G	F	N	C	Y	F	F	L	Q	S	Y	G	F	A	P	T	N	G	V	G	O	P	Y	R	V	V	L	S	F	E	L	L	H	A	P	A	T	V	C	G	P	K							
WT	T	G	C	V	I	A	W	N	S	N	L	D	S	K	V	G	G	N	Y	L	R	L	R	F	K	S	N	L	K	P	F	E	R	D	I	S	T	E	I	Y	Q	A	G	S	T	P	C	N	G	V	G	F	N	C	Y	F	F	L	Q	S	Y	G	F	A	P	T	N	G	V	G	O	P	Y	R	V	V	L	S	F	E	L	L	H	A	P	A	T	V	C	G	P	K							
WT	T	G	C	V	I	A	W	N	S	N	L	D	S	K	V	G	G	N	Y	L	R	L	R	F	K	S	N	L	K	P	F	E	R	D	I	S	T	E	I	Y	Q	A	G	S	T	P	C	N	G	V	G	F	N	C	Y	F	F	L	Q	S	Y	G	F	A	P	T	N	G	V	G	O	P	Y	R	V	V	L	S	F	E	L	L	H	A	P	A	T	V	C	G	P	K							
WT	T	G	C	V	I	A	W	N	S	N	L	D	S	K	V	G	G	N	Y	L	R	L	R	F	K	S	N	L	K	P	F	E	R	D	I	S	T	E	I	Y	Q	A	G	S	T	P	C	N	G	V	G	F	N	C	Y	F	F	L	Q	S	Y	G	F	A	P	T	N	G	V	G	O	P	Y	R	V	V	L	S	F	E	L																		

Shoshany *et al.* (2026) Stringent Selection Drives Convergence Toward Omicron-like SARS-CoV-2 Receptor-Binding Motifs

[illegible]

Fig. S14 – Multiple sequence alignment of selected 30 highest-frequency clones identified by Illumina sequencing from the LSS libraries (WT, Alpha, Beta, RBD v48, BA.1). Positions identical to wild-type (WT) are indicated by a dot, while amino acid substitutions are shown explicitly. The WT, specific library starting sequence (Alpha, Beta, RBD v48), and BA.1 reference sequences are shown in the header. Residues corresponding to WT are highlighted in blue, while residues carrying mutations characteristic for a given variant are highlighted in yellow. The number displayed to the left of each sequence indicates the absolute number of occurrences of that identical clone observed in the dataset.

Shoshany *et al.* (2026) Stringent Selection Drives Convergence Toward Omicron-like SARS-CoV-2 Receptor-Binding Motifs

WT unsorted	position																																																																																																																																																																																																																																																																																																																																																																																																																																																																																																																																																																																																																																																																																																																																																																																																																																																																																																																																																																																																																																																																																																																																																																																																																																																																																																																																																																																																																																																																																																																																																																																																																																																																																																																																																																																																																																																																																																																																																																																																																																																																																																																																																																																																																																																																																																																																																																																																																																						
WT	BA.1	BA.2																																																																																																																																																																																																																																																																																																																																																																																																																																																																																																																																																																																																																																																																																																																																																																																																																																																																																																																																																																																																																																																																																																																																																																																																																																																																																																																																																																																																																																																																																																																																																																																																																																																																																																																																																																																																																																																																																																																																																																																																																																																																																																																																																																																																																																																																																																																																																																																																																																					
186398x	TGC	TC	TG	CG	VA	AWNS	N	X	L	DS	KV	GG	NY	NY	L	Y	L	R	L	R	K	S	N	L	K	P	F	E	R	D	S	T	E	I	Y	Q	A	G	N	X	L	DS	KV	GG	NY	NY	L	Y	L	R	L	R	K	S	N	L	K	P	F	E	R	D	S	T	E	I	Y	Q	A	G	N	X	L	DS	KV	GG	NY	NY	L	Y	L	R	L	R	K	S	N	L	K	P	F	E	R	D	S	T	E	I	Y	Q	A	G	N	X	L	DS	KV	GG	NY	NY	L	Y	L	R	L	R	K	S	N	L	K	P	F	E	R	D	S	T	E	I	Y	Q	A	G	N	X	L	DS	KV	GG	NY	NY	L	Y	L	R	L	R	K	S	N	L	K	P	F	E	R	D	S	T	E	I	Y	Q	A	G	N	X	L	DS	KV	GG	NY	NY	L	Y	L	R	L	R	K	S	N	L	K	P	F	E	R	D	S	T	E	I	Y	Q	A	G	N	X	L	DS	KV	GG	NY	NY	L	Y	L	R	L	R	K	S	N	L	K	P	F	E	R	D	S	T	E	I	Y	Q	A	G	N	X	L	DS	KV	GG	NY	NY	L	Y	L	R	L	R	K	S	N	L	K	P	F	E	R	D	S	T	E	I	Y	Q	A	G	N	X	L	DS	KV	GG	NY	NY	L	Y	L	R	L	R	K	S	N	L	K	P	F	E	R	D	S	T	E	I	Y	Q	A	G	N	X	L	DS	KV	GG	NY	NY	L	Y	L	R	L	R	K	S	N	L	K	P	F	E	R	D	S	T	E	I	Y	Q	A	G	N	X	L	DS	KV	GG	NY	NY	L	Y	L	R	L	R	K	S	N	L	K	P	F	E	R	D	S	T	E	I	Y	Q	A	G	N	X	L	DS	KV	GG	NY	NY	L	Y	L	R	L	R	K	S	N	L	K	P	F	E	R	D	S	T	E	I	Y	Q	A	G	N	X	L	DS	KV	GG	NY	NY	L	Y	L	R	L	R	K	S	N	L	K	P	F	E	R	D	S	T	E	I	Y	Q	A	G	N	X	L	DS	KV	GG	NY	NY	L	Y	L	R	L	R	K	S	N	L	K	P	F	E	R	D	S	T	E	I	Y	Q	A	G	N	X	L	DS	KV	GG	NY	NY	L	Y	L	R	L	R	K	S	N	L	K	P	F	E	R	D	S	T	E	I	Y	Q	A	G	N	X	L	DS	KV	GG	NY	NY	L	Y	L	R	L	R	K	S	N	L	K	P	F	E	R	D	S	T	E	I	Y	Q	A	G	N	X	L	DS	KV	GG	NY	NY	L	Y	L	R	L	R	K	S	N	L	K	P	F	E	R	D	S	T	E	I	Y	Q	A	G	N	X	L	DS	KV	GG	NY	NY	L	Y	L	R	L	R	K	S	N	L	K	P	F	E	R	D	S	T	E	I	Y	Q	A	G	N	X	L	DS	KV	GG	NY	NY	L	Y	L	R	L	R	K	S	N	L	K	P	F	E	R	D	S	T	E	I	Y	Q	A	G	N	X	L	DS	KV	GG	NY	NY	L	Y	L	R	L	R	K	S	N	L	K	P	F	E	R	D	S	T	E	I	Y	Q	A	G	N	X	L	DS	KV	GG	NY	NY	L	Y	L	R	L	R	K	S	N	L	K	P	F	E	R	D	S	T	E	I	Y	Q	A	G	N	X	L	DS	KV	GG	NY	NY	L	Y	L	R	L	R	K	S	N	L	K	P	F	E	R	D	S	T	E	I	Y	Q	A	G	N	X	L	DS	KV	GG	NY	NY	L	Y	L	R	L	R	K	S	N	L	K	P	F	E	R	D	S	T	E	I	Y	Q	A	G	N	X	L	DS	KV	GG	NY	NY	L	Y	L	R	L	R	K	S	N	L	K	P	F	E	R	D	S	T	E	I	Y	Q	A	G	N	X	L	DS	KV	GG	NY	NY	L	Y	L	R	L	R	K	S	N	L	K	P	F	E	R	D	S	T	E	I	Y	Q	A	G	N	X	L	DS	KV	GG	NY	NY	L	Y	L	R	L	R	K	S	N	L	K	P	F	E	R	D	S	T	E	I	Y	Q	A	G	N	X	L	DS	KV	GG	NY	NY	L	Y	L	R	L	R	K	S	N	L	K	P	F	E	R	D	S	T	E	I	Y	Q	A	G	N	X	L	DS	KV	GG	NY	NY	L	Y	L	R	L	R	K	S	N	L	K	P	F	E	R	D	S	T	E	I	Y	Q	A	G	N	X	L	DS	KV	GG	NY	NY	L	Y	L	R	L	R	K	S	N	L	K	P	F	E	R	D	S	T	E	I	Y	Q	A	G	N	X	L	DS	KV	GG	NY	NY	L	Y	L	R	L	R	K	S	N	L	K	P	F	E	R	D	S	T	E	I	Y	Q	A	G	N	X	L	DS	KV	GG	NY	NY	L	Y	L	R	L	R	K	S	N	L	K	P	F	E	R	D	S	T	E	I	Y	Q	A	G	N	X	L	DS	KV	GG	NY	NY	L	Y	L	R	L	R	K	S	N	L	K	P	F	E	R	D	S	T	E	I	Y	Q	A	G	N	X	L	DS	KV	GG	NY	NY	L	Y	L	R	L	R	K	S	N	L	K	P	F	E	R	D	S	T	E	I	Y	Q	A	G	N	X	L	DS	KV	GG	NY	NY	L	Y	L	R	L	R	K	S	N	L	K	P	F	E	R	D	S	T	E	I	Y	Q	A	G	N	X	L	DS	KV	GG	NY	NY	L	Y	L	R	L	R	K	S	N	L	K	P	F	E	R	D	S	T	E	I	Y	Q	A	G	N	X	L	DS	KV	GG	NY	NY	L	Y	L	R	L	R	K	S	N	L	K	P	F	E	R	D	S	T	E	I	Y	Q	A	G	N	X	L	DS	KV	GG	NY	NY	L	Y	L	R	L	R	K	S	N	L	K	P	F	E	R	D	S	T	E	I	Y	Q	A	G	N	X	L	DS	KV	GG	NY	NY	L	Y	L	R	L	R	K	S	N	L	K	P	F	E	R	D	S	T	E	I	Y	Q	A	G	N	X	L	DS	KV	GG	NY	NY	L	Y	L	R	L	R	K	S	N	L	K	P	F	E	R	D	S	T	E	I	Y	Q	A	G	N	X	L	DS	KV	GG	NY	NY	L	Y	L	R	L	R	K	S	N	L	K	P	F	E	R	D	S	T	E	I	Y	Q	A	G	N	X	L	DS	KV	GG	NY	NY	L	Y	L	R	L	R	K	S	N	L	K	P	F	E	R	D	S	T	E	I	Y	Q	A	G	N	X	L	DS	KV	GG	NY	NY	L	Y	L	R	L	R	K	S	N	L	K	P	F	E	R	D	S	T	E	I	Y	Q	A	G	N	X	L	DS	KV	GG	NY	NY	L	Y	L	R	L	R	K	S	N	L	K	P	F	E	R	D	S	T	E	I	Y	Q	A	G	N	X	L	DS	KV	GG	NY	NY	L	Y	L	R	L	R	K	S	N	L	K	P	F	E	R	D	S	T	E	I	Y	Q	A	G	N	X	L	DS	KV	GG	NY	NY	L	Y	L	R	L	R	K	S	N	L	K	P	F	E	R	D	S	T	E	I	Y	Q	A	G	N	X	L	DS	KV	GG	NY	NY	L	Y	L	R	L	R	K	S	N	L	K	P	F	E	R	D	S	T	E	I	Y	Q	A	G	N	X	L	DS	KV	GG	NY	NY	L	Y	L	R	L	R	K	S	N	L	K	P	F	E	R	D	S	T	E	I	Y	Q	A	G	N	X	L	DS	KV	GG	NY	NY	L	Y	L	R	L	R	K	S	N	L	K	P	F	E	R	D	S	T	E	I	Y	Q	A	G	N	X	L	DS	KV	GG	NY	NY	L	Y	L	R	L	R	K	S	N	L	K	P	F	E	R	D	S	T	E	I	Y	Q	A	G	N	X	L	DS	KV	GG	NY	NY	L	Y	L	R	L	R	K	S	N	L	K	P	F	E	R	D	S	T	E	I	Y	Q	A	G	N	X	L	DS	KV	GG	NY	NY	L	Y	L	R	L	R	K	S	N	L	K	P	F	E	R	D	S	T	E	I	Y	Q	A	G	N	X	L	DS	KV	GG	NY	NY	L	Y	L	R	L	R	K	S	N	L	K	P	F	E	R	D	S	T	E	I	Y	Q	A	G	N	X	L	DS	KV	GG	NY	NY	L	Y	L	R	L	R	K	S	N	L	K	P	F	E	R	D	S	T	E	I	Y	Q	A	G	N	X	L	DS	KV	GG	NY	NY	L	Y	L	R	L	R	K	S	N	L	K	P	F	E	R	D	S	T	E	I	Y	Q	A	G	N	X	L	DS	KV	GG	NY	NY	L	Y	L	R	L	R	K	S	N	L	K	P	F	E	R	D	S	T	E	I	Y	Q	A	G	N	X	L	DS	KV	GG	NY	NY	L	Y	L	R	L	R	K	S	N	L	K	P	F	E	R	D	S	T	E	I	Y	Q	A	G	N	X	L	DS	KV	GG	NY	NY	L	Y	L	R	L	R	K	S	N	L	K	P	F	E	R	D	S	T	E	I	Y	Q	A	G	N	X	L	DS	KV	GG	NY	NY	L	Y	L	R	L	R	K	S	N	L	K	P	F	E	R	D	S	T	E	I	Y	Q	A	G	N	X	L	DS	KV	GG	NY	NY	L	Y	L	R	L	R	K	S	N	L	K	P	F	E	R	D	S	T	E	I	Y	Q	A	G	N	X	L	DS	KV	GG	NY	NY	L	Y	L	R	L	R	K	S	N	L	K	P	F	E	R	D	S	T	E	I	Y	Q	A	G	N	X	L	DS	KV	GG	NY	NY	L	Y	L	R	L	R	K	S	N	L	K	P	F	E	R	D	S	T	E	I	Y	Q	A	G	N	X	L	DS	KV	GG	NY	NY	L	Y	L	R	L	R	K	S	N	L	K	P	F	E	R	D	S	T	E	I	Y	Q	A	G	N	X	L	DS	KV	GG	NY	NY	L	Y	L	R	L	R	K	S	N	L	K	P	F	E	R	D	S	T	E	I	Y	Q	A	G	N	X	L	DS	KV	GG	NY	NY	L	Y	L	R	L	R	K	S	N	L	K	P	F	E	R	D	S	T	E	I	Y	Q	A	G	N	X	L	DS	KV	GG	NY	NY	L	Y	L	R	L	R	K	S	N	L	K	P	F	E	R	D	S	T	E	I	Y	Q	A	G	N	X	L	DS	KV	GG	NY	NY	L	Y	L	R	L	R	K	S	N	L	K	P	F	E	R	D	S	T	E	I	Y	Q	A	G	N	X	L	DS	KV	GG	NY	NY	L	Y	L	R	L	R	K	S	N	L	K	P	F	E	R	D	S	T	E	I	Y	Q	A	G	N	X	L	DS	KV	GG	NY	NY	L	Y	L	R	L	R	K	S	N	L	K	P	F	E	R	D	S	T	E	I	Y	Q	A	G	N	X	L	DS	KV	GG	NY	NY	L	Y	L	R	L	R	K	S	N	L	K	P	F	E	R	D	S	T	E	I	Y	Q	A	G	N	X	L	DS	KV	GG	NY	NY	L	Y	L	R	L	R	K	S	N	L	K	P	F	E	R	D	S	T	E	I	Y	Q	A	G	N	X	L	DS	KV	GG	NY	NY	L	Y	L	R	L	R	K	S	N	L	K	P	F	E	R	D	S	T	E	I	Y	Q	A	G	N	X	L	DS	KV	GG	NY	NY	L	Y	L	R	L	R	K	S	N	L	K	P	F	E	R	D	S	T	E	I	Y	Q	A	G	N	X	L	DS	KV	GG	NY	NY	L	Y	L	R	L	R	K	S	N	L	K	P	F	E	R	D	S	T	E	I	Y	Q	A	G	N

Fig. S15 – Multiple sequence alignment of selected 30 highest-frequency clones identified by Illumina sequencing from the WT HSS libraries (unsorted, round 2 and round 4). Positions identical to wild-type (WT) are indicated by a dot, while amino acid substitutions are shown explicitly. The WT, BA.1, and BA.2 reference sequences are shown in the header. Residues corresponding to WT are highlighted in blue, while residues carrying mutations characteristic for a given variant are highlighted in yellow. The number displayed to the left of each sequence indicates the absolute number of occurrences of that identical clone observed in the dataset.

Shoshany *et al.* (2026) Stringent Selection Drives Convergence Toward Omicron-like SARS-CoV-2 Receptor-Binding Motifs

135F unsorted									
res. Number.	WT	BA.1	BA.2						
430	TGC	TGC	TGC						
431	CG	CG	CG						
432	CA	CA	CA						
433	CA	CA	CA						
434	CA	CA	CA						
435	CA	CA	CA						
436	CA	CA	CA						
437	CA	CA	CA						
438	CA	CA	CA						
439	CA	CA	CA						
440	CA	CA	CA						
441	CA	CA	CA						
442	CA	CA	CA						
443	CA	CA	CA						
444	CA	CA	CA						
445	CA	CA	CA						
446	CA	CA	CA						
447	CA	CA	CA						
448	CA	CA	CA						
449	CA	CA	CA						
450	CA	CA	CA						
451	CA	CA	CA						
452	CA	CA	CA						
453	CA	CA	CA						
454	CA	CA	CA						
455	CA	CA	CA						
456	CA	CA	CA						
457	CA	CA	CA						
458	CA	CA	CA						
459	CA	CA	CA						
460	CA	CA	CA						
461	CA	CA	CA						
462	CA	CA	CA						
463	CA	CA	CA						
464	CA	CA	CA						
465	CA	CA	CA						
466	CA	CA	CA						
467	CA	CA	CA						
468	CA	CA	CA						
469	CA	CA	CA						
470	CA	CA	CA						
471	CA	CA	CA						
472	CA	CA	CA						
473	CA	CA	CA						
474	CA	CA	CA						
475	CA	CA	CA						
476	CA	CA	CA						
477	CA	CA	CA						
478	CA	CA	CA						
479	CA	CA	CA						
480	CA	CA	CA						
481	CA	CA	CA						
482	CA	CA	CA						
483	CA	CA	CA						
484	CA	CA	CA						
485	CA	CA	CA						
486	CA	CA	CA						
487	CA	CA	CA						
488	CA	CA	CA						
489	CA	CA	CA						
490	CA	CA	CA						
491	CA	CA	CA						
492	CA	CA	CA						
493	CA	CA	CA						
494	CA	CA	CA						
495	CA	CA	CA						
496	CA	CA	CA						
497	CA	CA	CA						
498	CA	CA	CA						
499	CA	CA	CA						
500	CA	CA	CA						
501	CA	CA	CA						
502	CA	CA	CA						
503	CA	CA	CA						
504	CA	CA	CA						
505	CA	CA	CA						
506	CA	CA	CA						
507	CA	CA	CA						
508	CA	CA	CA						
509	CA	CA	CA						
510	CA	CA	CA						
511	CA	CA	CA						
512	CA	CA	CA						
513	CA	CA	CA						
514	CA	CA	CA						
515	CA	CA	CA						
516	CA	CA	CA						
517	CA	CA	CA						
518	CA	CA	CA						
519	CA	CA	CA						
520	CA	CA	CA						
521	CA	CA	CA						
522	CA	CA	CA						
523	CA	CA	CA						
524	CA	CA	CA						
525	CA	CA	CA						
526	CA	CA	CA						
527	CA	CA	CA						
528	CA	CA	CA						

135F 2nd									
res. Number.	WT	BA.1	BA.2						
430	TGC	TGC	TGC						
431	CG	CG	CG						
432	CA	CA	CA						
433	CA	CA	CA						
434	CA	CA	CA						
435	CA	CA	CA						
436	CA	CA	CA						
437	CA	CA	CA						
438	CA	CA	CA						
439	CA	CA	CA						
440	CA	CA	CA						
441	CA	CA	CA						
442	CA	CA	CA						
443	CA	CA	CA						
444	CA	CA	CA						
445	CA	CA	CA						
446	CA	CA	CA						
447	CA	CA	CA						
448	CA	CA	CA						
449	CA	CA	CA						
450	CA	CA	CA						
451	CA	CA	CA						
452	CA	CA	CA						
453	CA	CA	CA						
454	CA	CA	CA						
455	CA	CA	CA						
456	CA	CA	CA						
457	CA	CA	CA						
458	CA	CA	CA						
459	CA	CA	CA						
460	CA	CA	CA						
461	CA	CA	CA						
462	CA	CA	CA						
463	CA	CA	CA						
464	CA	CA	CA						
465	CA	CA	CA						
466	CA	CA	CA						
467	CA	CA	CA						
468	CA	CA	CA						
469	CA	CA	CA						
470	CA	CA	CA						
471	CA	CA	CA						
472	CA	CA	CA						
473	CA	CA	CA						
474	CA	CA	CA						
475	CA	CA	CA						
476	CA	CA	CA						
477	CA	CA	CA						
478	CA	CA	CA						
479	CA	CA	CA						
480	CA	CA	CA						
481	CA	CA	CA						
482	CA	CA	CA						
483	CA	CA	CA						
484	CA	CA	CA						
485	CA	CA	CA						
486	CA	CA	CA						
487	CA	CA	CA						
488	CA	CA	CA						
489	CA	CA	CA						
490	CA	CA	CA						
491	CA	CA	CA						
492	CA	CA	CA						
493	CA	CA	CA						
494	CA	CA	CA						
495	CA	CA	CA						
496	CA	CA	CA						
497	CA	CA	CA						
498	CA	CA	CA						
499	CA	CA	CA						
500	CA	CA	CA						
501	CA	CA	CA						
502	CA	CA	CA						
503	CA	CA	CA						
504	CA	CA	CA						
505	CA	CA	CA						
506	CA	CA	CA						
507	CA	CA	CA						
508	CA	CA	CA						
509	CA	CA	CA						
510	CA	CA	CA						
511	CA	CA	CA						
512	CA	CA	CA						
513	CA	CA	CA						
514	CA	CA	CA						
515	CA	CA	CA						
516	CA	CA	CA						
517	CA	CA	CA						
518	CA	CA	CA						
519	CA	CA	CA						
520	CA	CA	CA						
521	CA	CA	CA						
522	CA	CA	CA						
523	CA	CA	CA						
524	CA	CA	CA						
525	CA	CA	CA						
526	CA	CA	CA						
527	CA	CA	CA						
528	CA	CA	CA						

135F 4th									
res. Number.	WT	BA.1	BA.2						
430	TGC	TGC	TGC						
431	CG	CG	CG						
432	CA	CA	CA						
433	CA	CA	CA						
434	CA	CA	CA						
435	CA	CA	CA						
436	CA	CA	CA						
437	CA	CA	CA						
438	CA	CA	CA						
439	CA	CA	CA						
440	CA	CA	CA						
441	CA	CA	CA						
442	CA	CA	CA						
443	CA	CA	CA						
444	CA	CA	CA						
445	CA	CA	CA						
446	CA	CA	CA						
447	CA	CA	CA						
448	CA	CA	CA						
449	CA	CA	CA						
450	CA	CA	CA						
451	CA	CA	CA						
452	CA	CA	CA						
453	CA	CA	CA						
454	CA	CA	CA						
455	CA	CA	CA						
456	CA	CA	CA						
457	CA	CA	CA				</		

Fig. S16 – Multiple sequence alignment of selected 30 highest-frequency clones identified by Illumina sequencing from the I358F HSS libraries (unsorted, round 2 and round 4). Positions identical to wild-type (WT) are indicated by a dot, while amino acid substitutions are shown explicitly. The WT, BA.1, and BA.2 reference sequences are shown in the header. Residues corresponding to WT are highlighted in blue, while residues carrying mutations characteristic for a given variant are highlighted in yellow. The number displayed to the left of each sequence indicates the absolute number of occurrences of that identical clone observed in the dataset.

Shoshany *et al.* (2026) Stringent Selection Drives Convergence Toward Omicron-like SARS-CoV-2 Receptor-Binding Motifs

[illegible]

Fig. S17 – Multiple sequence alignment of selected 30 highest-frequency clones identified by Illumina sequencing from the BA.1 HSS libraries (unsorted, round 2 and round 4). Positions identical to wild-type (WT) are indicated by a dot, while amino acid substitutions are shown explicitly. The WT, BA.1, and BA.2 reference sequences are shown in the header. Residues corresponding to WT are highlighted in blue, while residues carrying mutations characteristic for a given variant are highlighted in yellow. The number displayed to the left of each sequence indicates the absolute number of occurrences of that identical clone observed in the dataset.

Shoshany *et al.* (2026) Stringent Selection Drives Convergence Toward Omicron-like SARS-CoV-2 Receptor-Binding Motifs

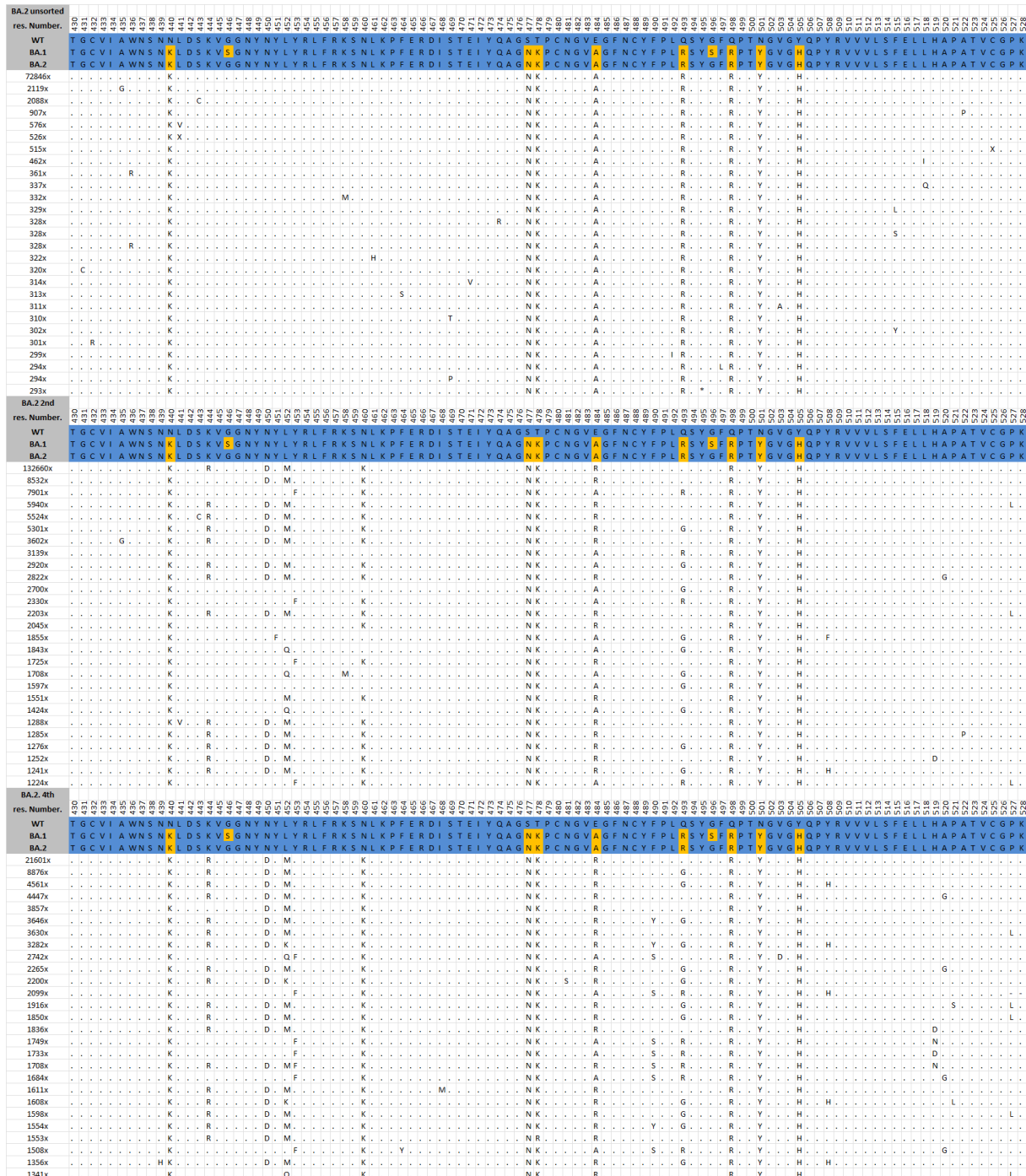

Fig. S18 – Multiple sequence alignment of selected 30 highest-frequency clones identified by Illumina sequencing from the BA.2 HSS libraries (unsorted, round 2 and round 4). Positions identical to wild-type (WT) are indicated by a dot, while amino acid substitutions are shown explicitly. The WT, BA.1, and BA.2 reference sequences are shown in the header. Residues corresponding to WT are highlighted in blue, while residues carrying mutations characteristic for a given variant are highlighted in yellow. The number displayed to the left of each sequence indicates the absolute number of occurrences of that identical clone observed in the dataset.

Supporting information part PS3 – Analysis of mutations in low stringency selection libraries

A WT 7th library low stringency selection (LSS)

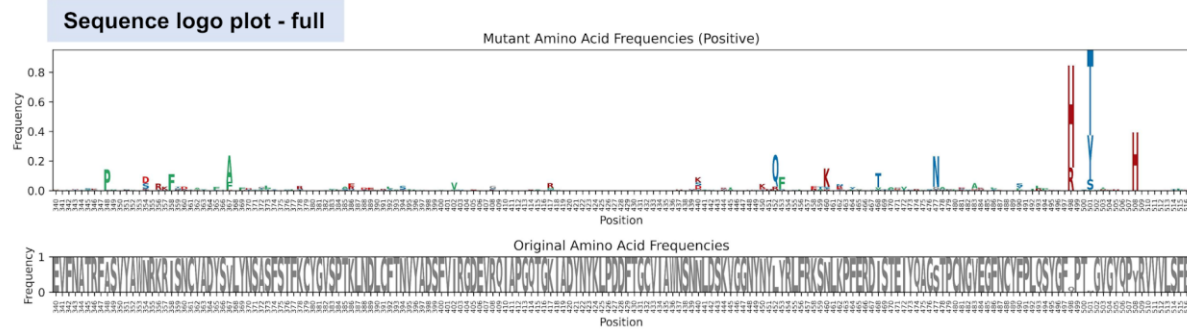

B Sequence logo plot – low freq detail

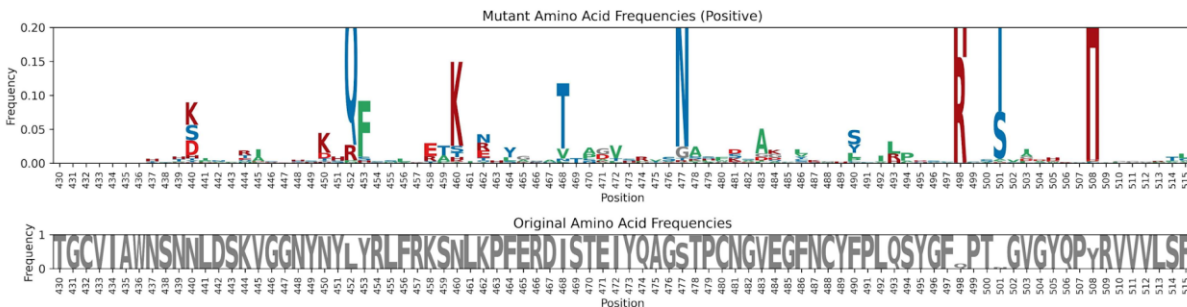

C Mutation scatter plot

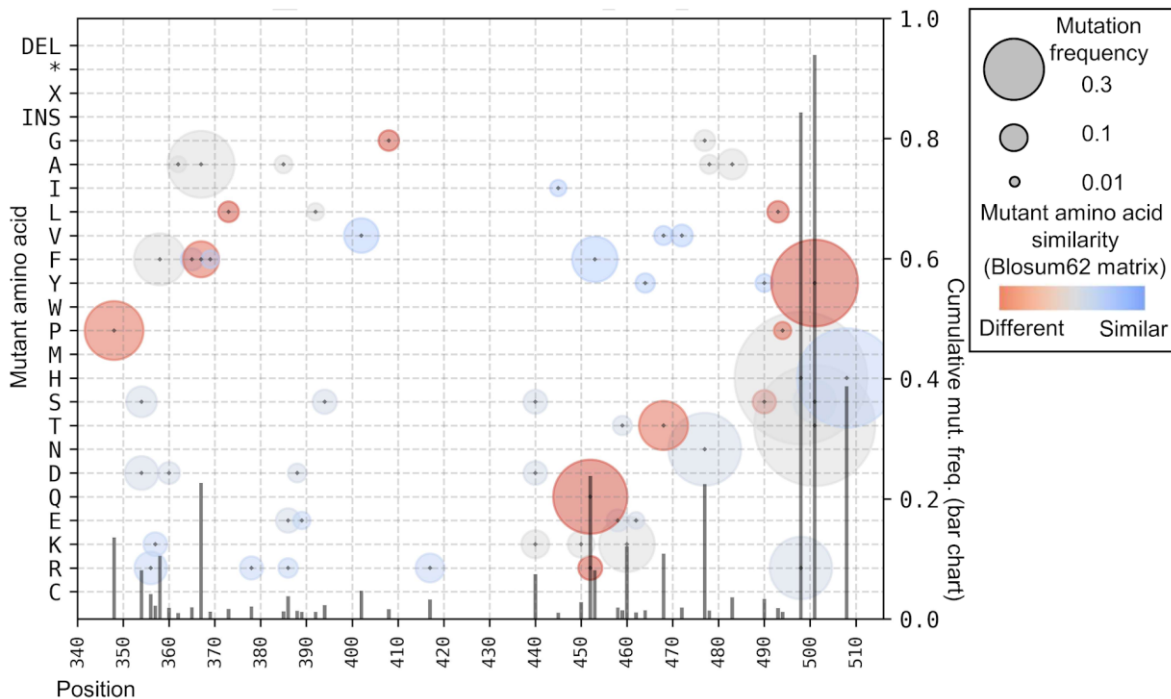

Fig. S19 – Logo plots and mutation scatter plot for WT 7th library under LSS. (A) Sequence logo plot (top panel) showing mutations in the library relative to the WT sequence. The bottom panel (in gray) displays the complementary frequency of the original amino acid at each position. (B) Sequence logo plot focusing on less frequent mutations in the library, with the y-axis frequency range set to 0–0.2. (C) Mutation scatter plot illustrating mutations in the population with a frequency ≥ 0.01 and their evolutionary distance from the original residues.

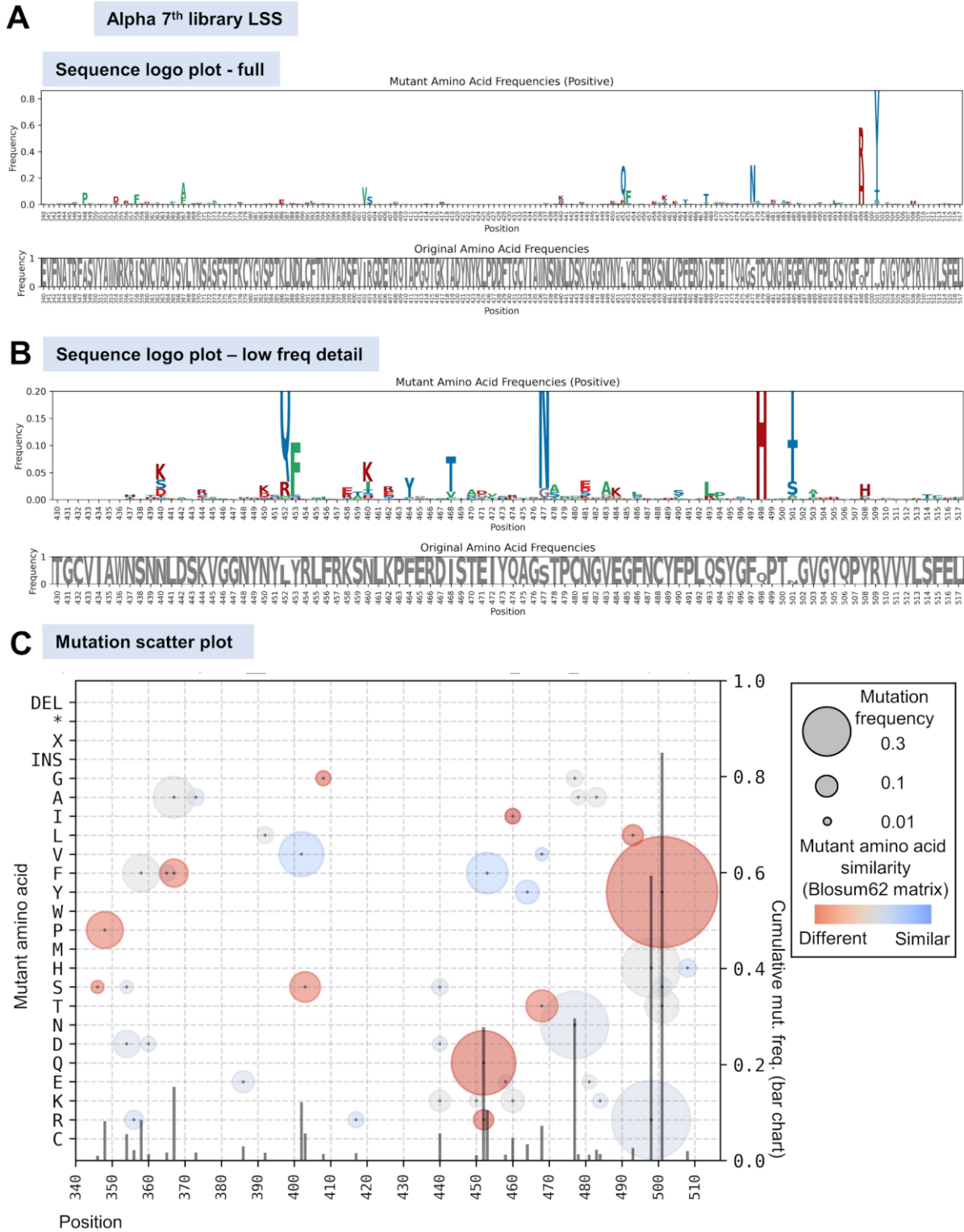

Fig. S20 – Logo plots and mutation scatter plot for Alpha 7th library under LSS. (A) Sequence logo plot (top panel) showing mutations in the library relative to the WT sequence. The bottom panel (in gray) displays the complementary frequency of the original amino acid at each position. (B) Sequence logo plot focusing on less frequent mutations in the library, with the y-axis frequency range set to 0–0.2. (C) Mutation scatter plot illustrating mutations in the population with a frequency ≥ 0.01 and their evolutionary distance from the original residues.

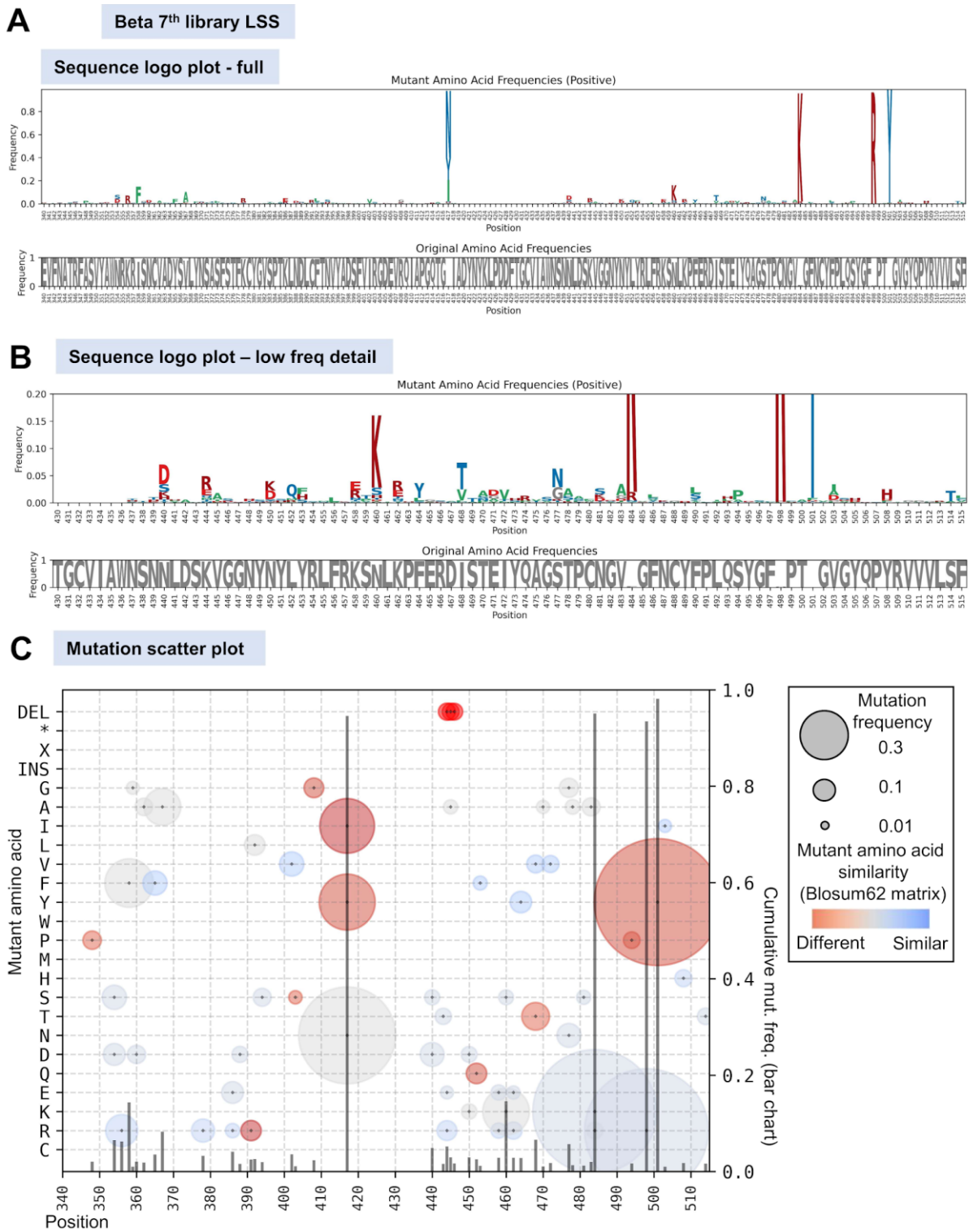

Fig. S21 – Logo plots and mutation scatter plot for Beta 7th library under LSS. (A) Sequence logo plot (top panel) showing mutations in the library relative to the WT sequence. The bottom panel (in gray) displays the complementary frequency of the original amino acid at each position. (B) Sequence logo plot focusing on less frequent mutations in the library, with the y-axis frequency range set to 0–0.2. (C) Mutation scatter plot illustrating mutations in the population with a frequency ≥ 0.01 and their evolutionary distance from the original residues.

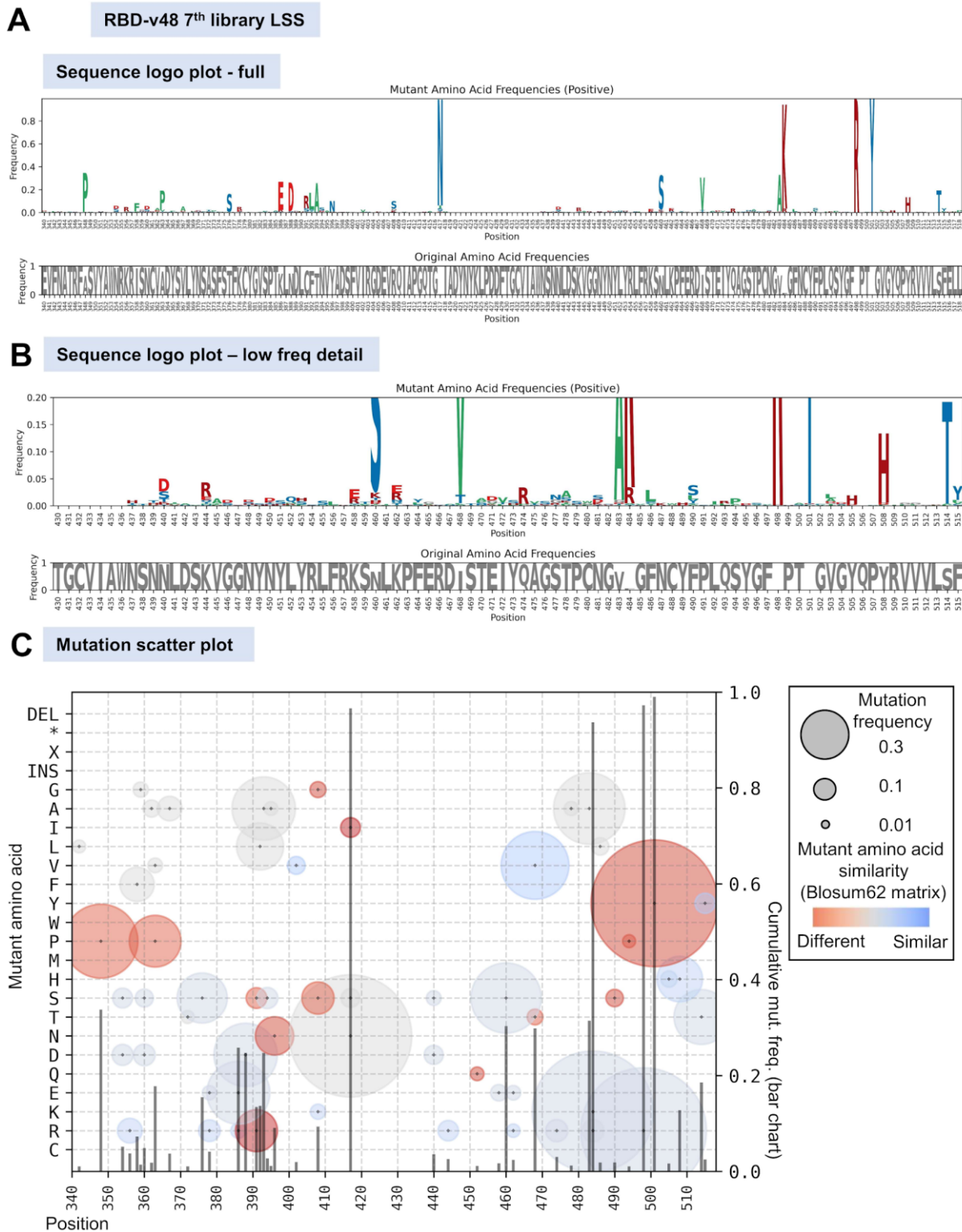

Fig. S22 – Logo plots and mutation scatter plot for v48 7th library under LSS. (A) Sequence logo plot (top panel) showing mutations in the library relative to the WT sequence. The bottom panel (in gray) displays the complementary frequency of the original amino acid at each position. (B) Sequence logo plot focusing on less frequent mutations in the library, with the y-axis frequency range set to 0–0.2. (C) Mutation scatter plot illustrating mutations in the population with a frequency ≥ 0.01 and their evolutionary distance from the original residues.

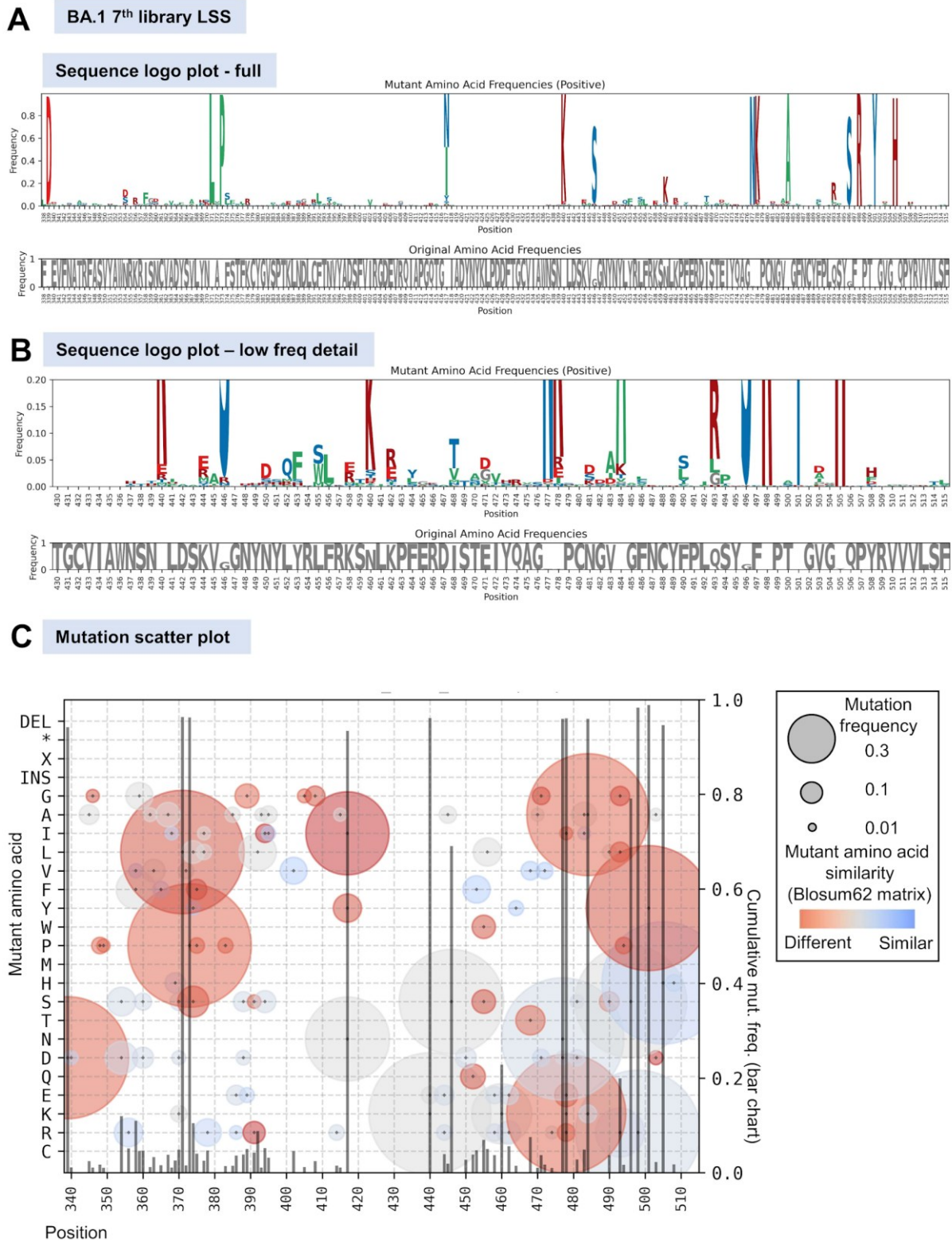

Fig. S23 – Logo plots and mutation scatter plot for BA.1 7th library under LSS. (A) Sequence logo plot (top panel) showing mutations in the library relative to the WT sequence. The bottom panel (in gray) displays the complementary frequency of the original amino acid at each position. (B) Sequence logo plot focusing on less frequent mutations in the library, with the y-axis frequency range set to 0–0.2. (C) Mutation scatter plot illustrating mutations in the population with a frequency ≥ 0.01 and their evolutionary distance from the original residues.

Supporting information part PS4 – Analysis of mutations at different stages of high stringency selection libraries

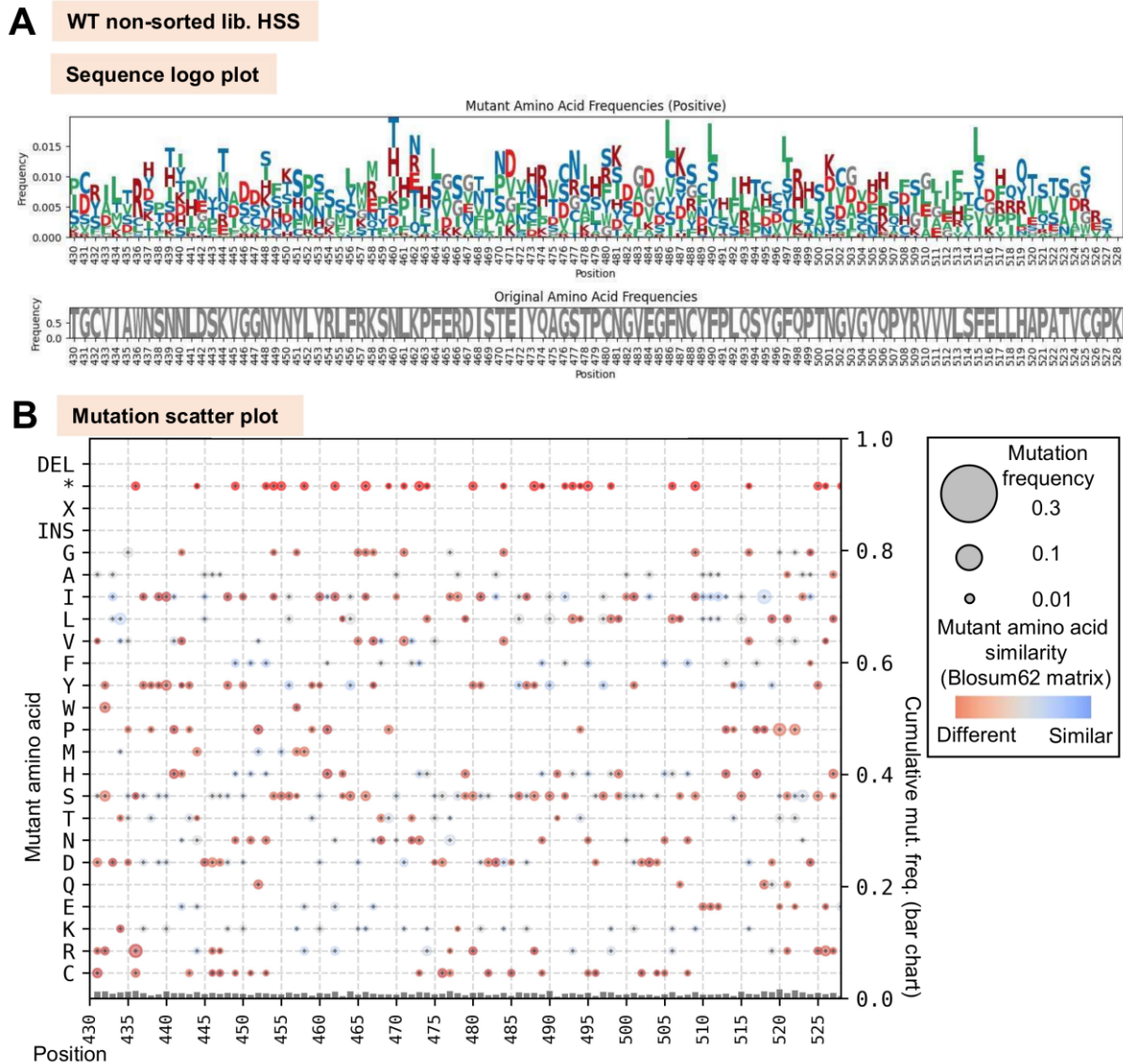

Fig. S24 – Logo plot and mutation scatter plot for WT non-selected library for HSS. (A) Sequence logo plot (top panel) showing mutations in the library relative to the WT sequence. The bottom panel (in gray) displays the complementary frequency of the original amino acid at each position. (B) Mutation scatter plot illustrating mutations in the population and their evolutionary distance from the original residues.

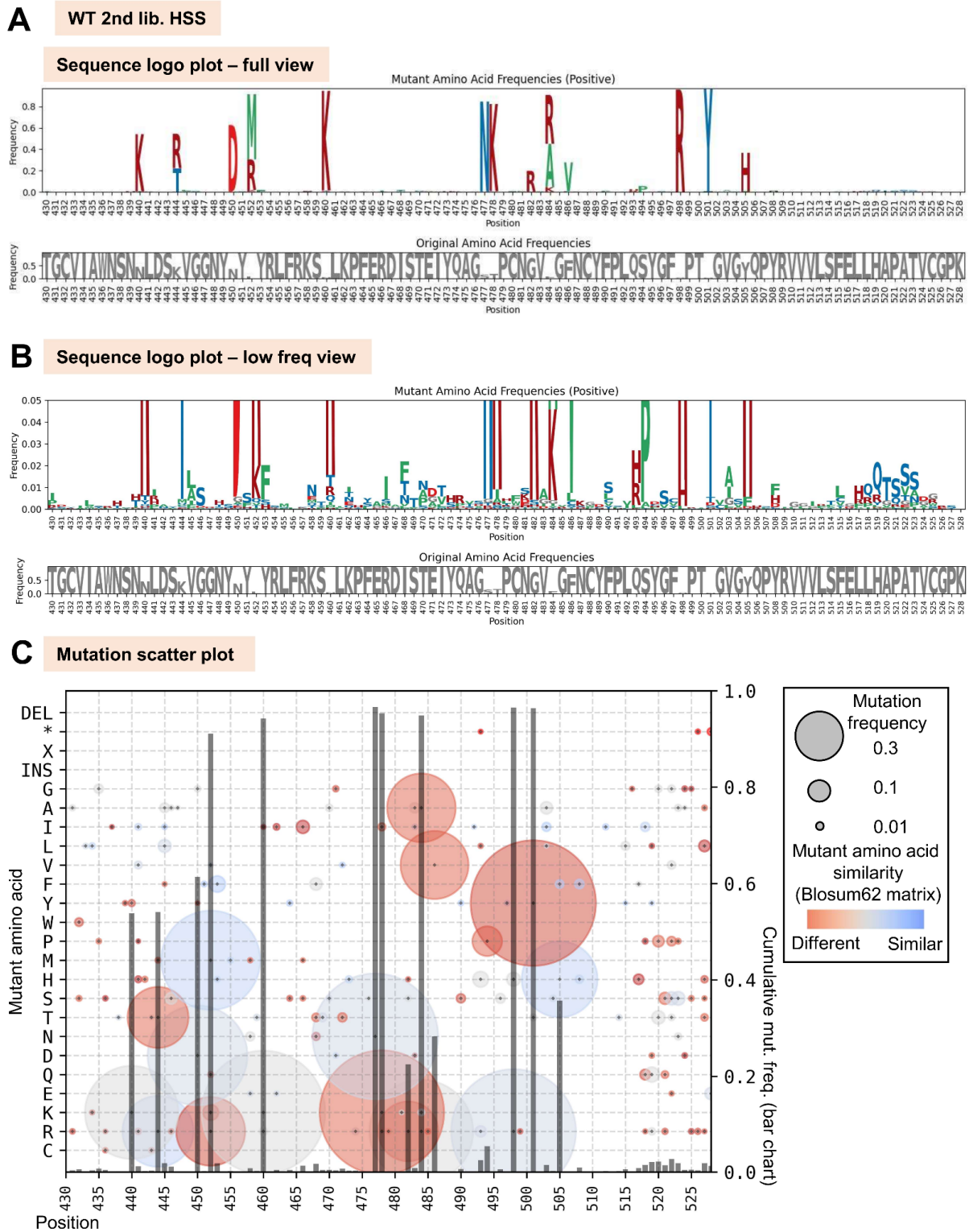

Fig. S25 – Logo plots and mutation scatter plot for WT 2nd library selected under HSS. (A) Sequence logo plot (top panel) showing mutations in the library relative to the WT sequence. The bottom panel (in gray) displays the complementary frequency of the original amino acid at each position. (B) Sequence logo plot focusing on less frequent mutations in the library, with the y-axis frequency range set to 0–0.2. (C) Mutation scatter plot illustrating mutations in the population and their evolutionary distance from the original residues.

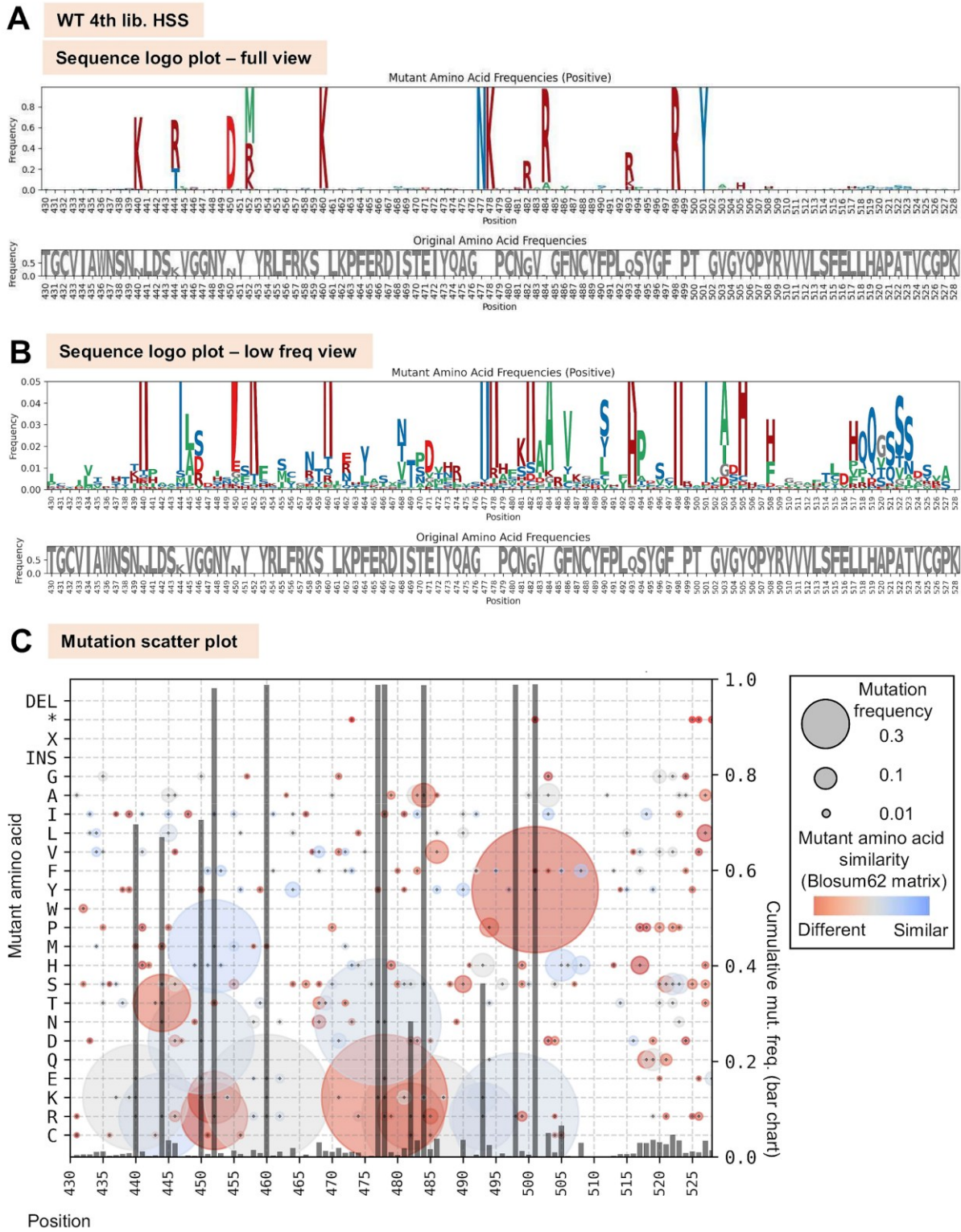

Fig. S26 – Logo plots and mutation scatter plot for WT 4th library selected under HSS. (A) Sequence logo plot (top panel) showing mutations in the library relative to the WT sequence. The bottom panel (in gray) displays the complementary frequency of the original amino acid at each position. (B) Sequence logo plot focusing on less frequent mutations in the library, with the y-axis frequency range set to 0–0.2. (C) Mutation scatter plot illustrating mutations in the population and their evolutionary distance from the original residues.

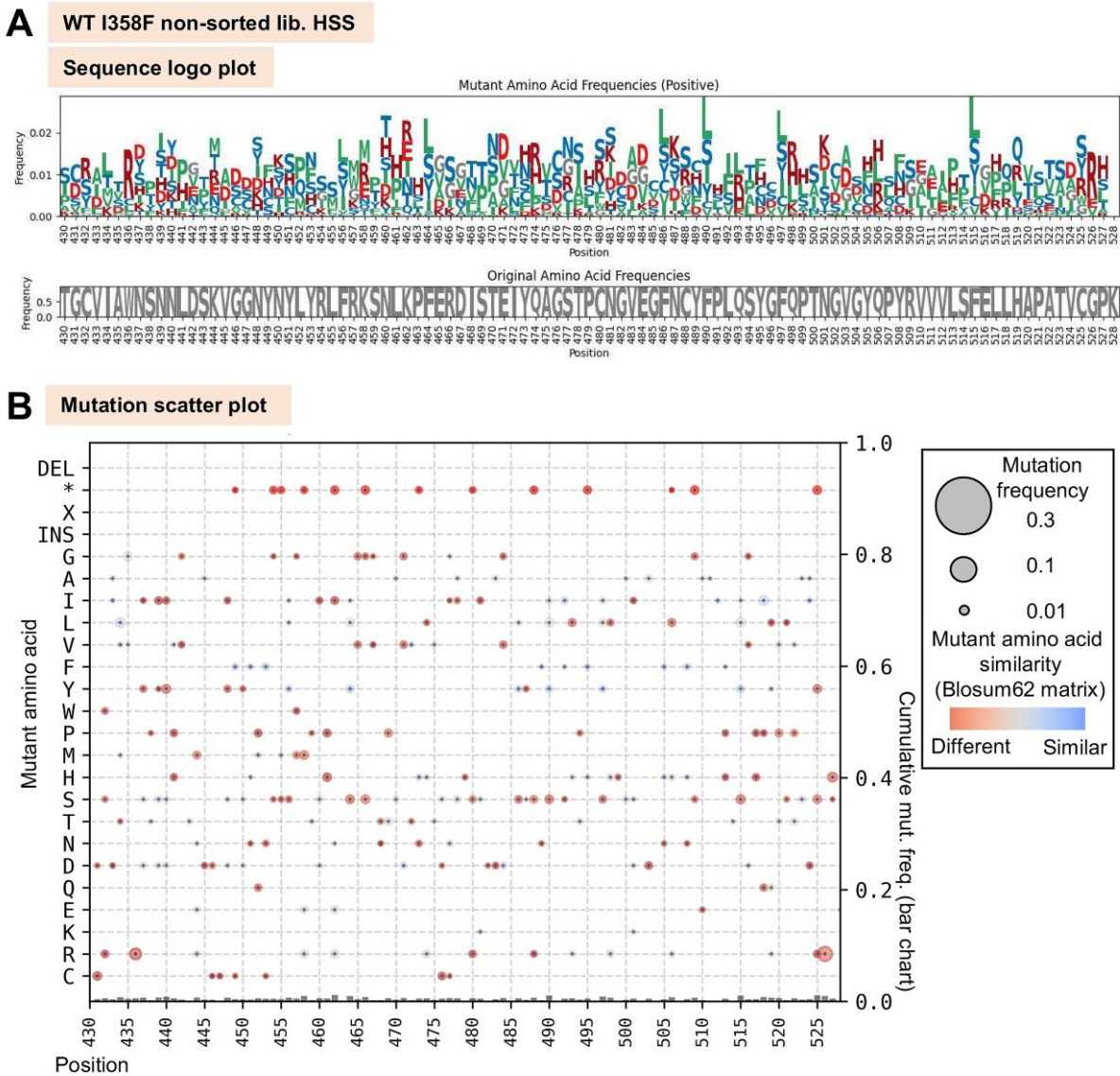

Fig. S27 – Logo plot and mutation scatter plot for WT I358F non-selected library for HSS. (A) Sequence logo plot (top panel) showing mutations in the library relative to the WT sequence. The bottom panel (in gray) displays the complementary frequency of the original amino acid at each position. (B) Mutation scatter plot illustrating mutations in the population and their evolutionary distance from the original residues.

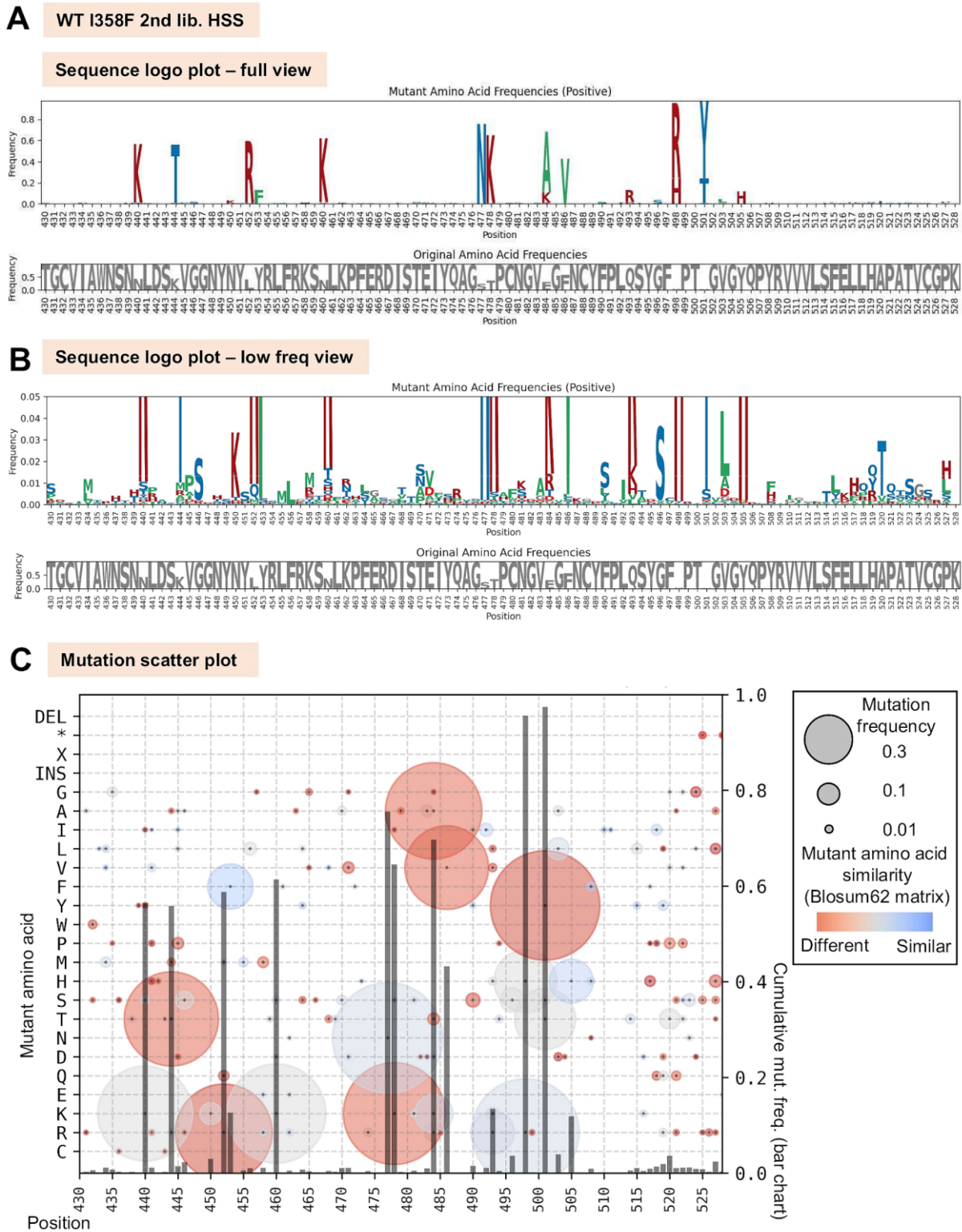

Fig. S28 – Logo plots and mutation scatter plot for WT I358F 2nd library selected under HSS. (A) Sequence logo plot (top panel) showing mutations in the library relative to the WT sequence. The bottom panel (in gray) displays the complementary frequency of the original amino acid at each position. (B) Sequence logo plot focusing on less frequent mutations in the library, with the y-axis frequency range set to 0–0.2. (C) Mutation scatter plot illustrating mutations in the population and their evolutionary distance from the original residues.

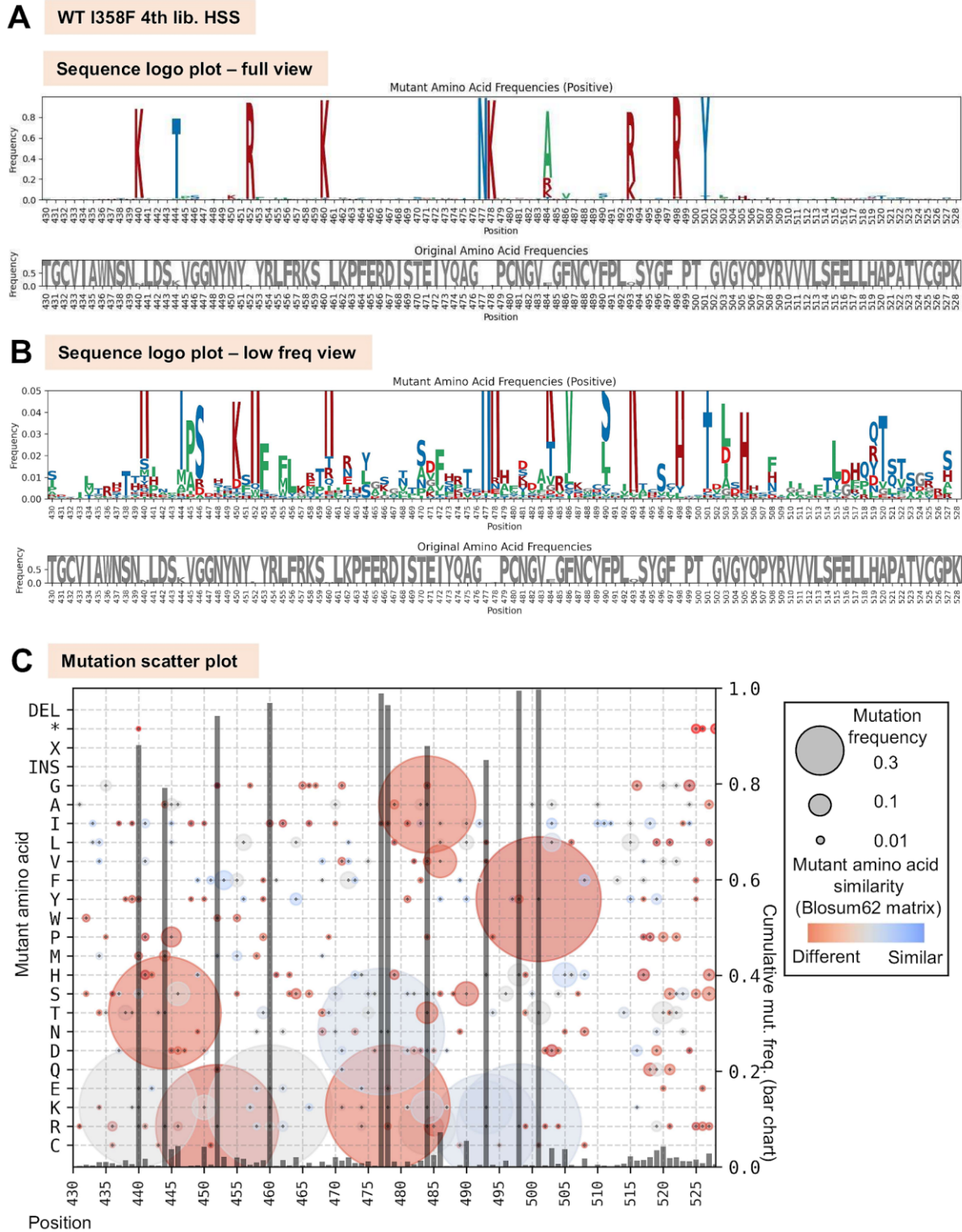

Fig. S29 – Logo plots and mutation scatter plot for WT I358F 4th library selected under HSS. (A) Sequence logo plot (top panel) showing mutations in the library relative to the WT sequence. The bottom panel (in gray) displays the complementary frequency of the original amino acid at each position. (B) Sequence logo plot focusing on less frequent mutations in the library, with the y-axis frequency range set to 0–0.2. (C) Mutation scatter plot illustrating mutations in the population and their evolutionary distance from the original residues.

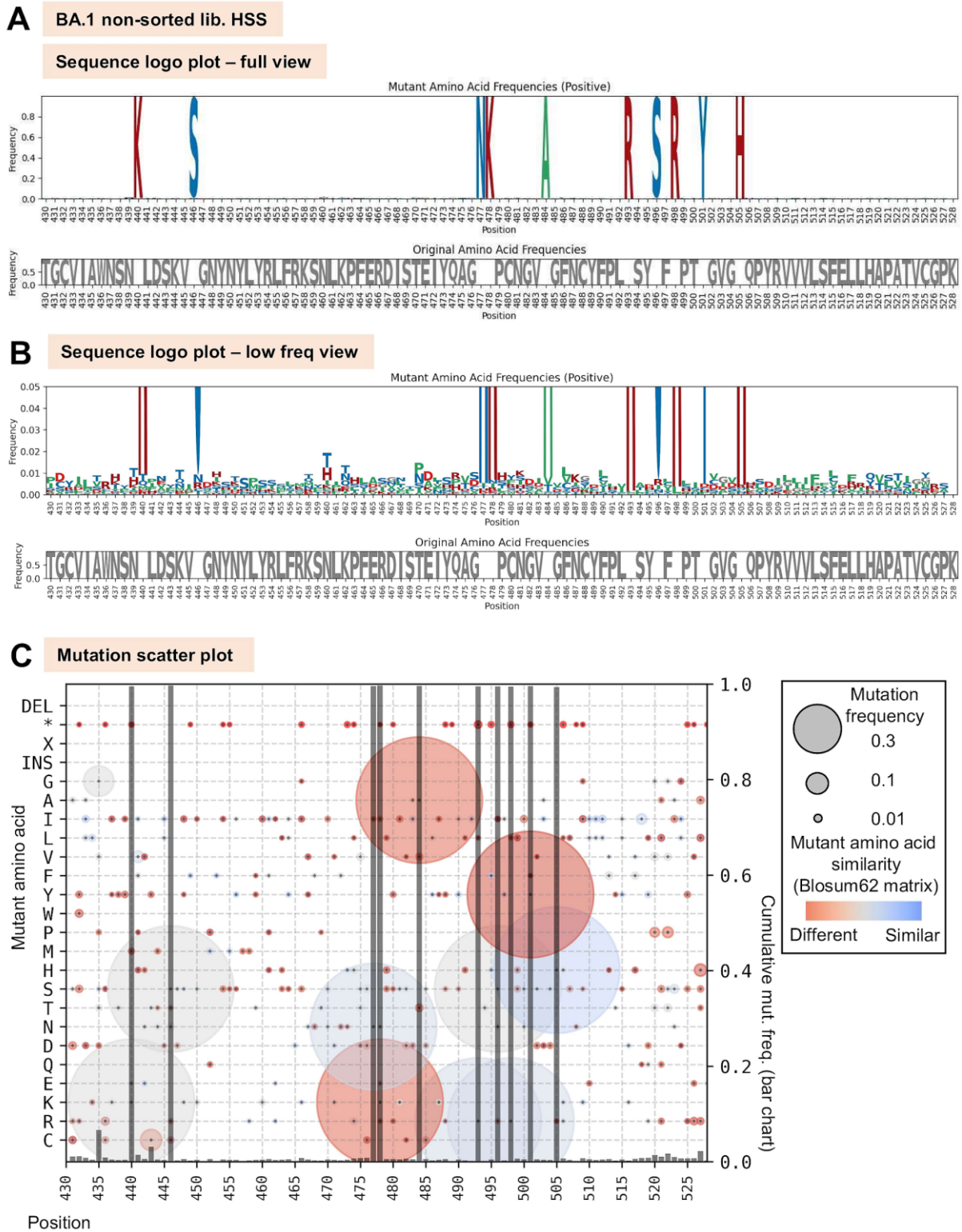

Fig. S30 – Logo plots and mutation scatter plot for BA.1 non-selected library selected under HSS. (A) Sequence logo plot (top panel) showing mutations in the library relative to the WT sequence. The bottom panel (in gray) displays the complementary frequency of the original amino acid at each position. (B) Sequence logo plot focusing on less frequent mutations in the library, with the y-axis frequency range set to 0–0.2. (C) Mutation scatter plot illustrating mutations in the population and their evolutionary distance from the original residues.

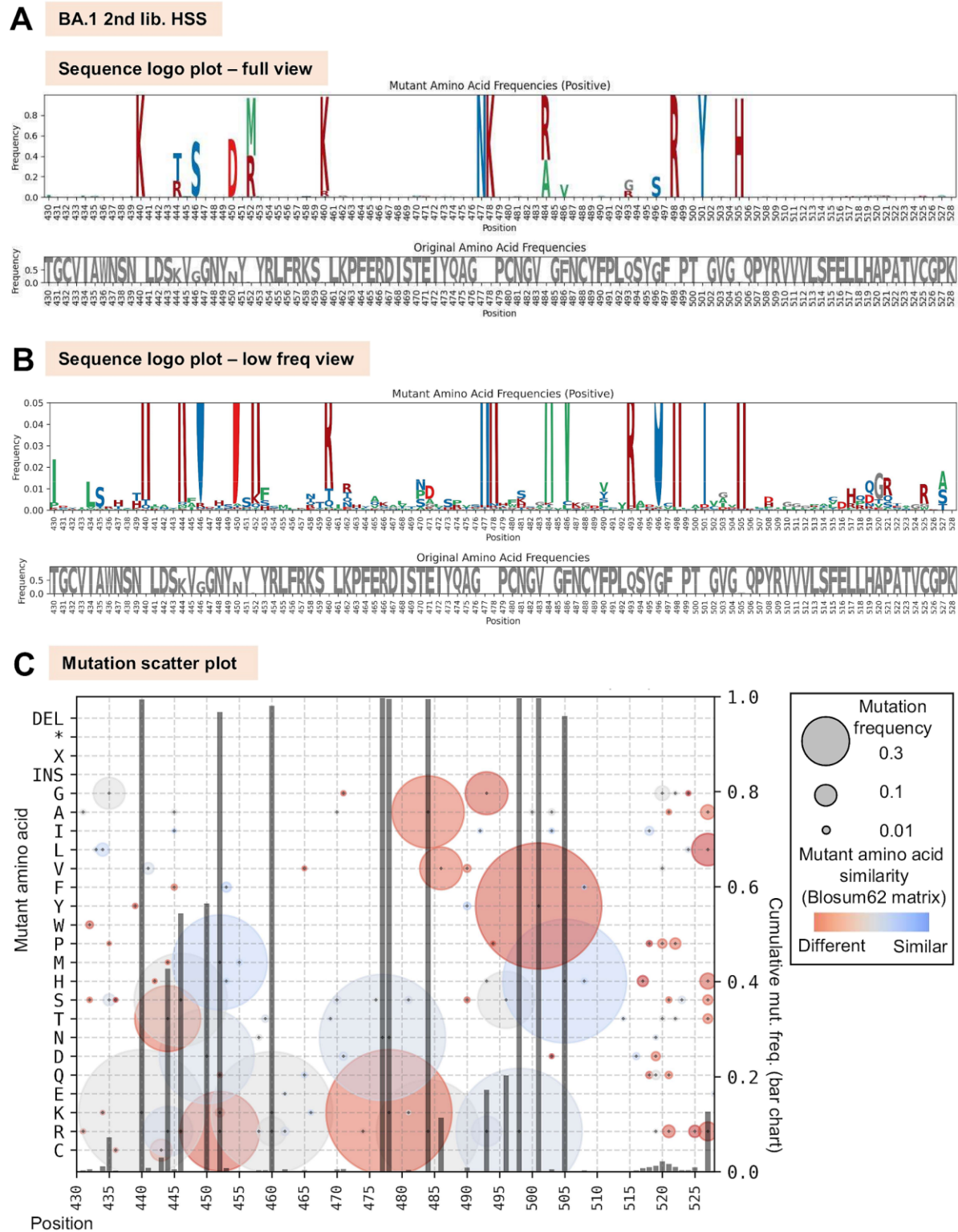

Fig. S31 – Logo plots and mutation scatter plot for BA.1 2nd library selected under HSS. (A) Sequence logo plot (top panel) showing mutations in the library relative to the WT sequence. The bottom panel (in gray) displays the complementary frequency of the original amino acid at each position. (B) Sequence logo plot focusing on less frequent mutations in the library, with the y-axis frequency range set to 0–0.2. (C) Mutation scatter plot illustrating mutations in the population and their evolutionary distance from the original residues.

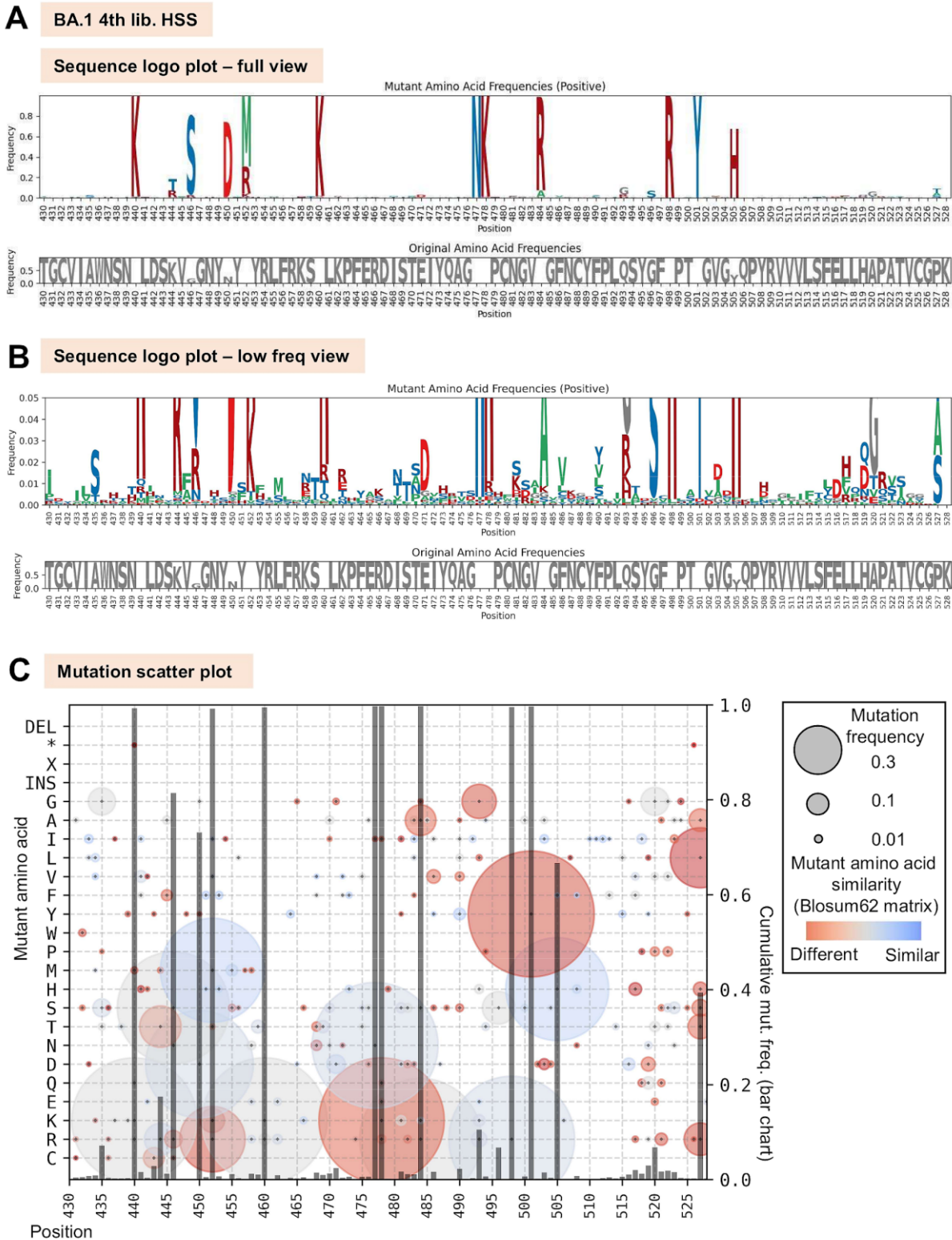

Fig. S32 – Logo plots and mutation scatter plot for BA.1 4th library selected under HSS. (A) Sequence logo plot (top panel) showing mutations in the library relative to the WT sequence. The bottom panel (in gray) displays the complementary frequency of the original amino acid at each position. (B) Sequence logo plot focusing on less frequent mutations in the library, with the y-axis frequency range set to 0–0.2. (C) Mutation scatter plot illustrating mutations in the population and their evolutionary distance from the original residues.

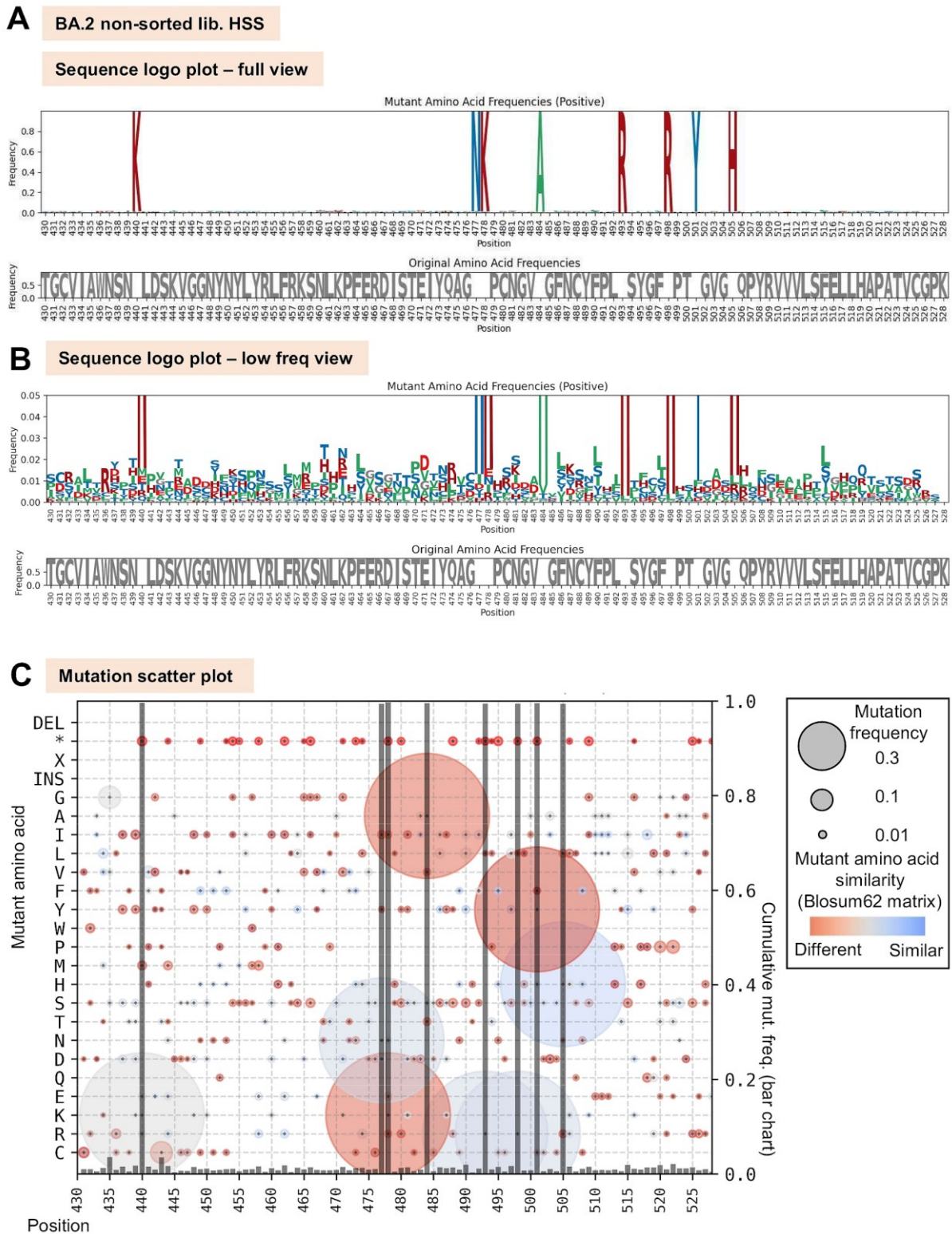

Fig. S33 – Logo plots and mutation scatter plot for BA.2 non-selected library for HSS. (A) Sequence logo plot (top panel) showing mutations in the library relative to the WT sequence. The bottom panel (in gray) displays the complementary frequency of the original amino acid at each position. (B) Sequence logo plot focusing on less frequent mutations in the library, with the y-axis frequency range set to 0–0.2. (C) Mutation scatter plot illustrating mutations in the population and their evolutionary distance from the original residues.

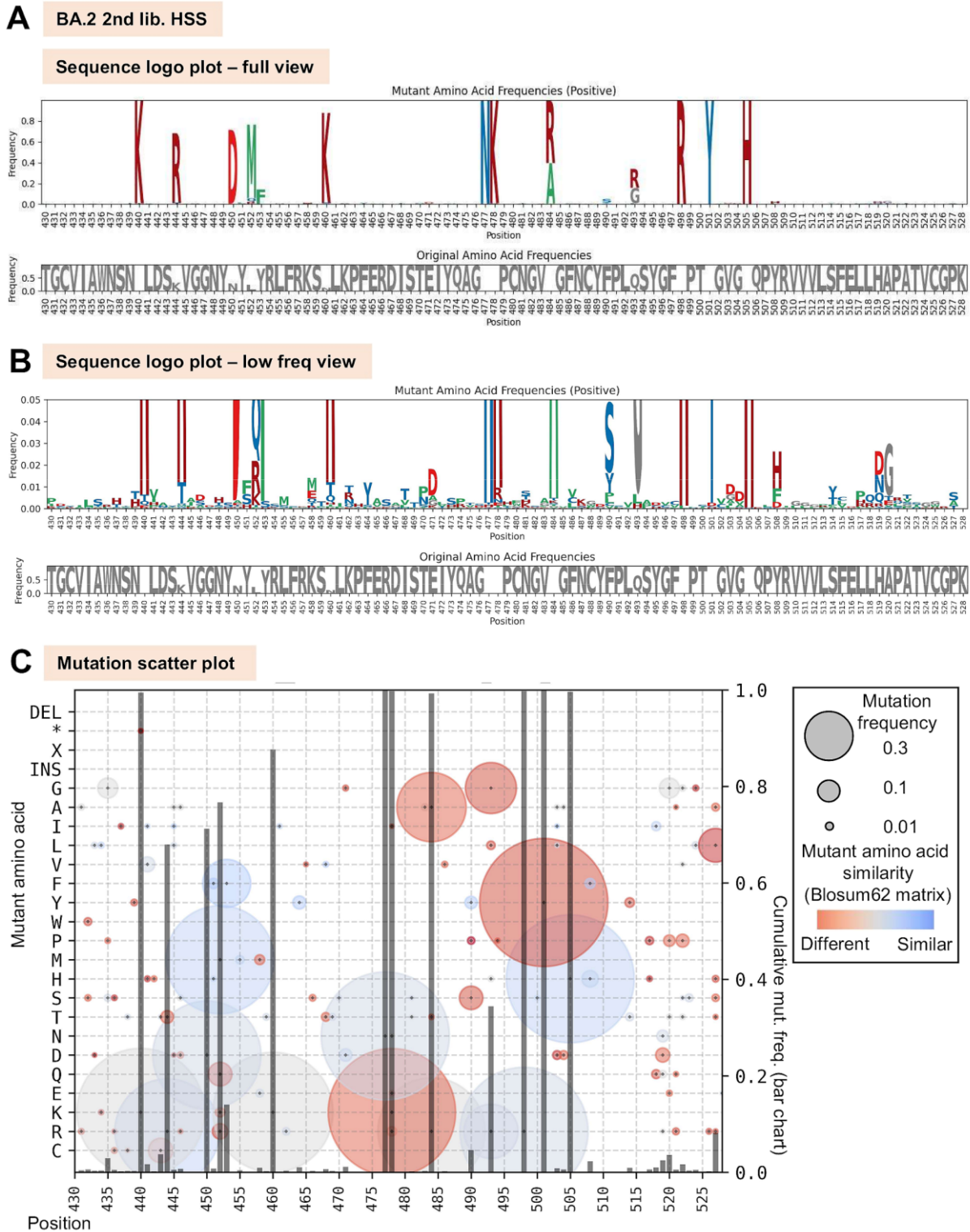

Fig. S34 – Logo plots and mutation scatter plot for BA.2 2nd library selected under HSS. (A) Sequence logo plot (top panel) showing mutations in the library relative to the WT sequence. The bottom panel (in gray) displays the complementary frequency of the original amino acid at each position. (B) Sequence logo plot focusing on less frequent mutations in the library, with the y-axis frequency range set to 0–0.2. (C) Mutation scatter plot illustrating mutations in the population and their evolutionary distance from the original residues.

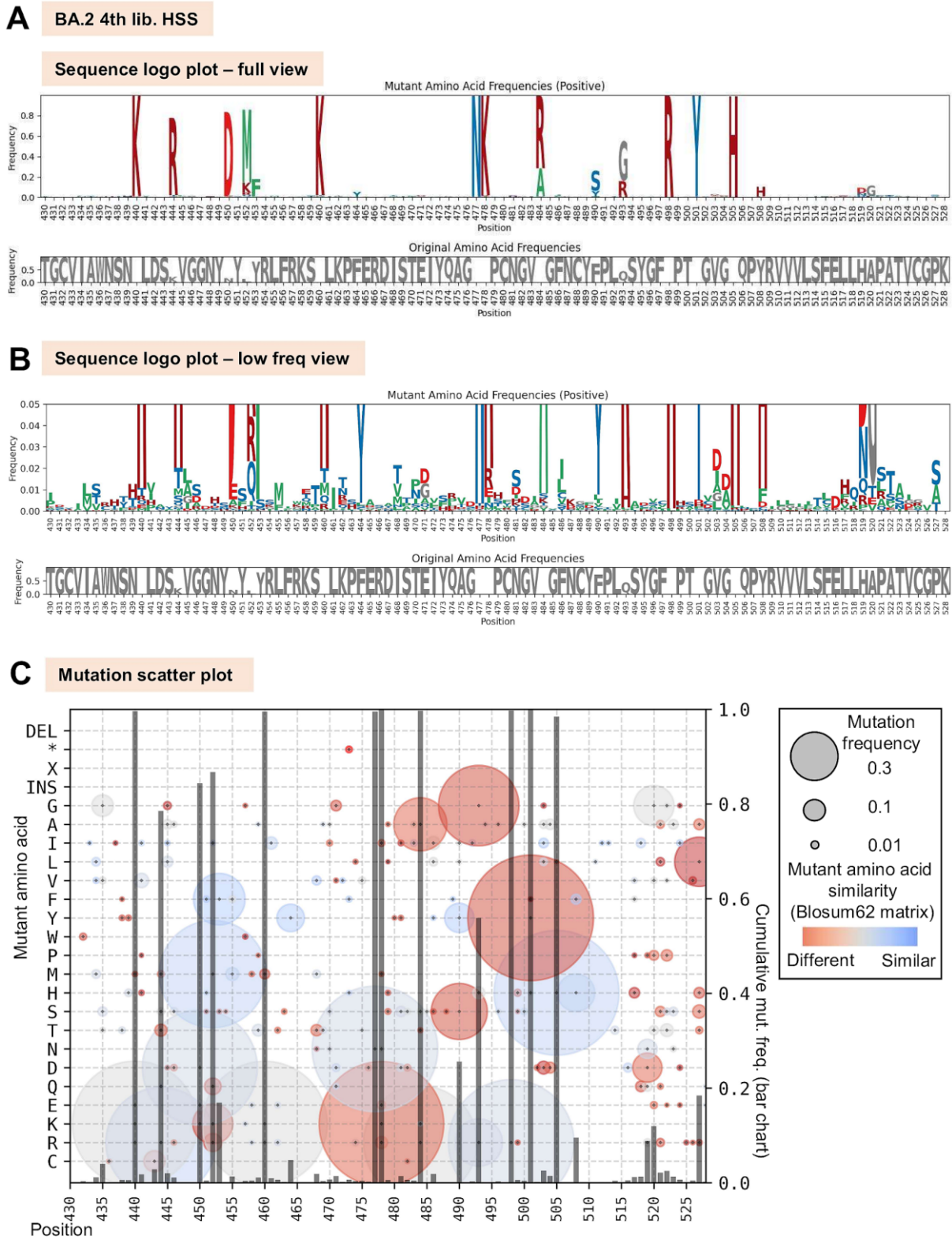

Fig. S35 – Logo plots and mutation scatter plot for BA.2 4th library selected under HSS. (A) Sequence logo plot (top panel) showing mutations in the library relative to the WT sequence. The bottom panel (in gray) displays the complementary frequency of the original amino acid at each position. (B) Sequence logo plot focusing on less frequent mutations in the library, with the y-axis frequency range set to 0–0.2. (C) Mutation scatter plot illustrating mutations in the population and their evolutionary distance from the original residues.

A All GISAID SARS-CoV-2 sequences (7th December 2024, GISAID_spikenuc1207 dataset)

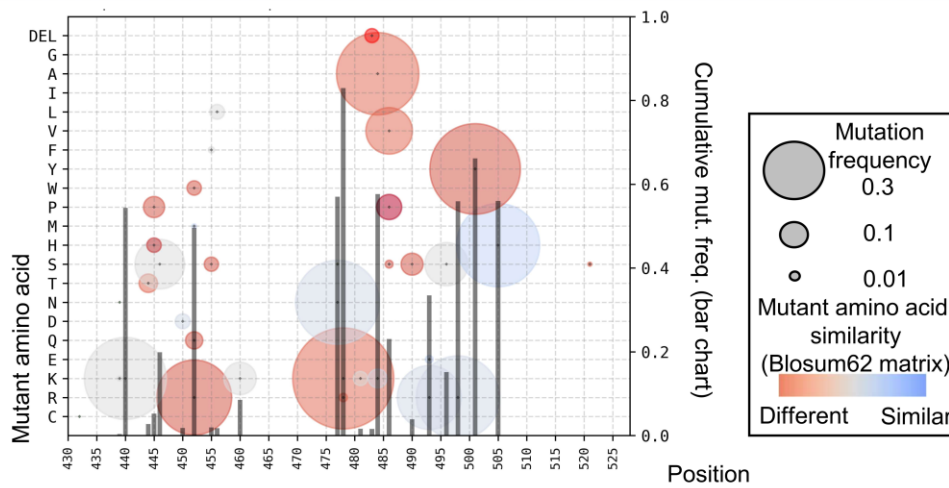

B Pango named SARS-CoV-2 lineages (12th September 2024)

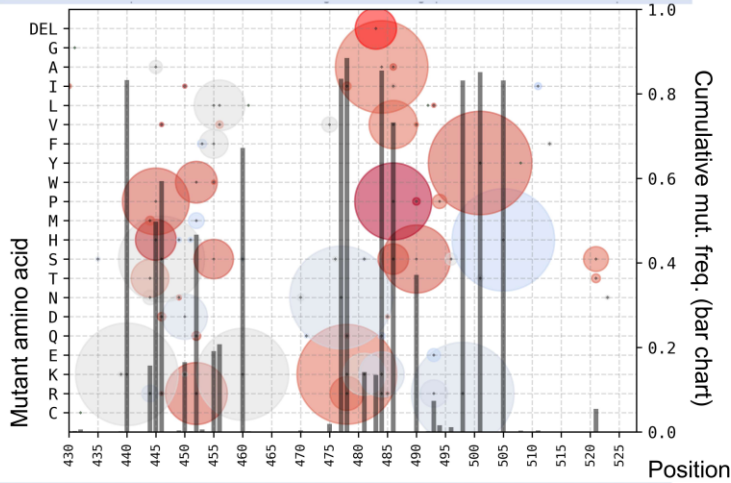

C Pango named SARS-CoV-2 lineages – sequence logo plot

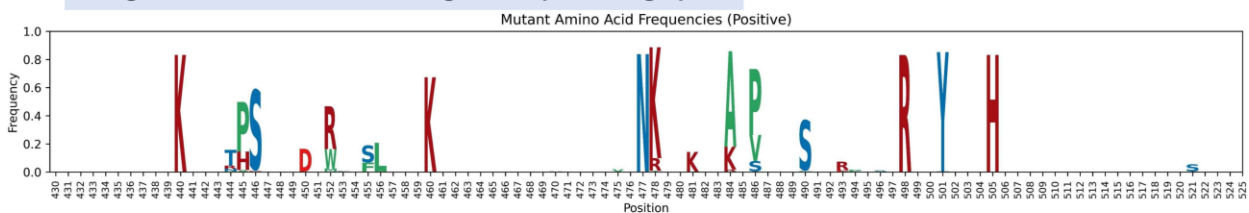

D Pango named SARS-CoV-2 lineages without BA.2.86 and descendants – sequence logo plot

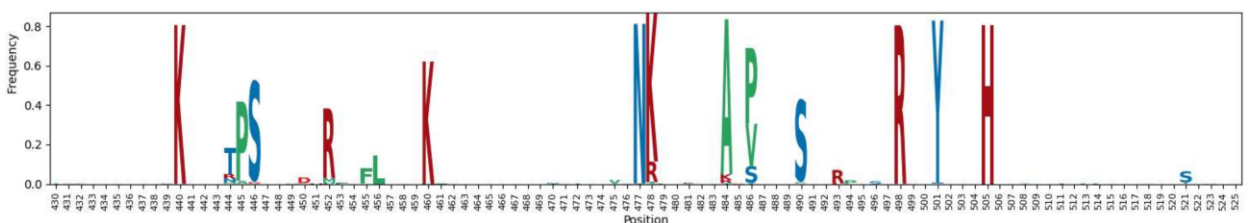

Fig. S36 – SARS-CoV-2 sequences and lineages analysis. (A) Full RBM mutation scatter plot for the complete set of all GISAID SARS-CoV-2 sequences above 0.1 % threshold obtained from a spikenuc1207 file released on December 7, 2024 by GISAID. (B) RBM (amino acids 430 – 528) mutation scatter plot for the complete set of all SARS-CoV-2 Pango named lineages (released on September 12, 2024) with lowered threshold 0.1 % due to disbalances introduced when lineages were merged into a single ancestral sequence, (C) Sequence logo plot for the complete set of all SARS-CoV-2 Pango named lineages. Deletion is not shown. (D) Sequence logo plot for the set of SARS-CoV-2 Pango named lineages without BA.2.86 and its descendants (“late” Omicron lineages).

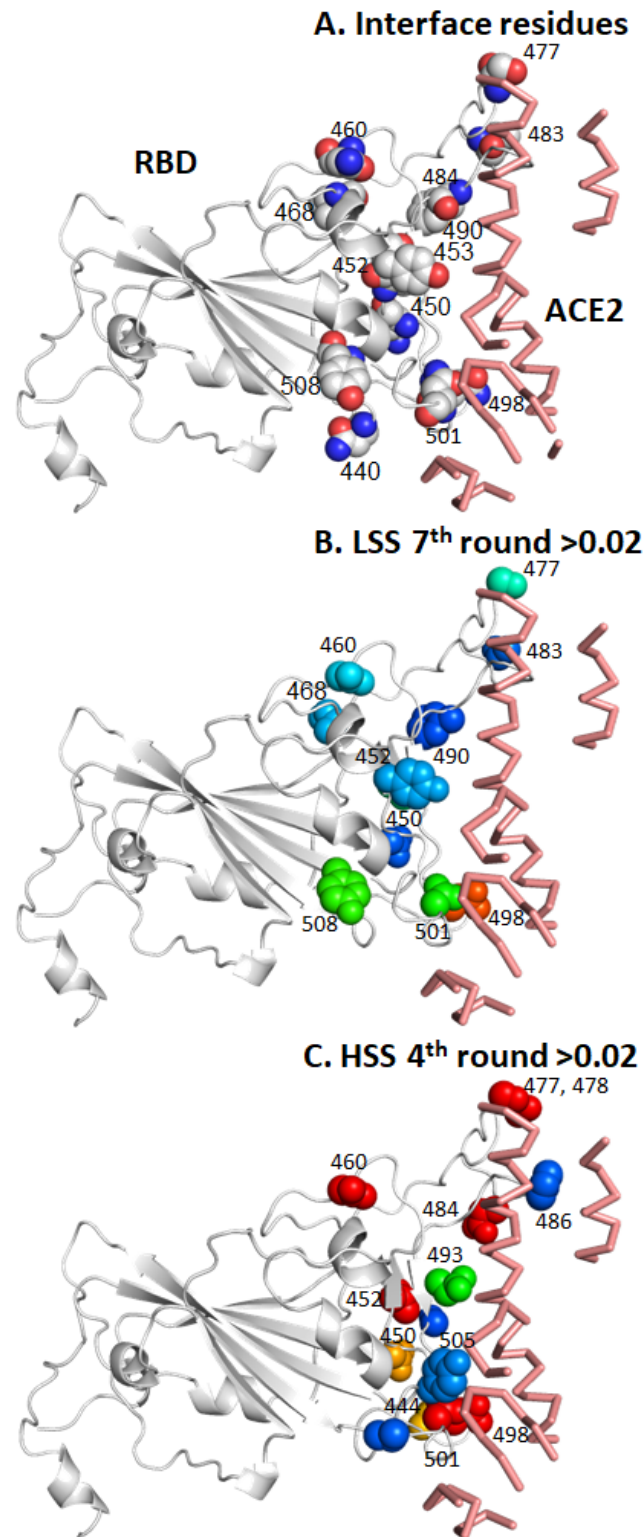

Fig. S37 – Structure representation of the mutations accumulated at higher frequencies following LSS and HSS. (A) Interface residues numbering based on Wuhan-Hu-1 (MN908947.3) Spike protein sequence similar to Jun Lan *et al.* (2020) Nature **581**: 215-220 (<https://www.nature.com/articles/s41586-020-2180-5>). (B, C) Residues with mutations frequencies >0.02, color coded according to frequency: blue (0.02) to red (1). Images were created by using PyMOL v3.1.6.1 and SARS-CoV RBD–ACE2 complex (PDB: 2AJF).

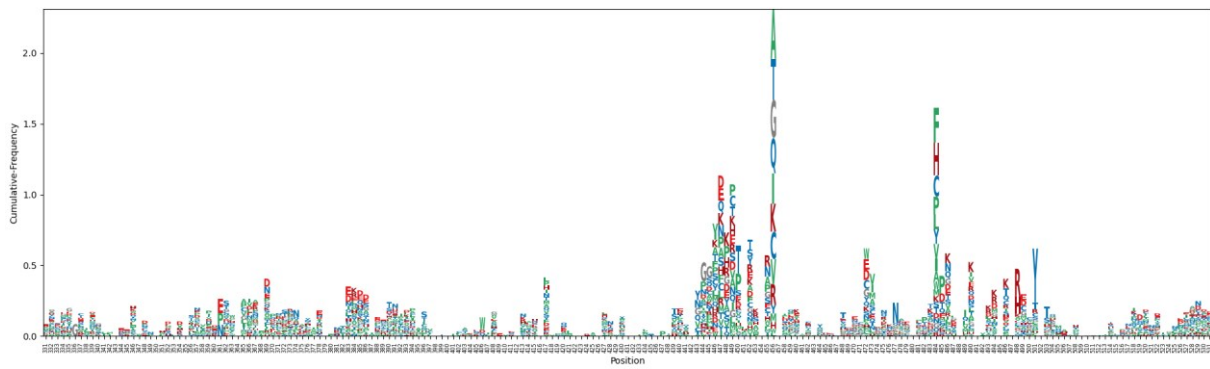

Fig. S38 – Sequence logo plot of RBD mutations that reduce human polyclonal plasma binding averaged across all individuals and time points. Data generated by Allison J. Greaney and colleagues (*Prof. Jesse D. Bloom laboratory*, ²⁴).

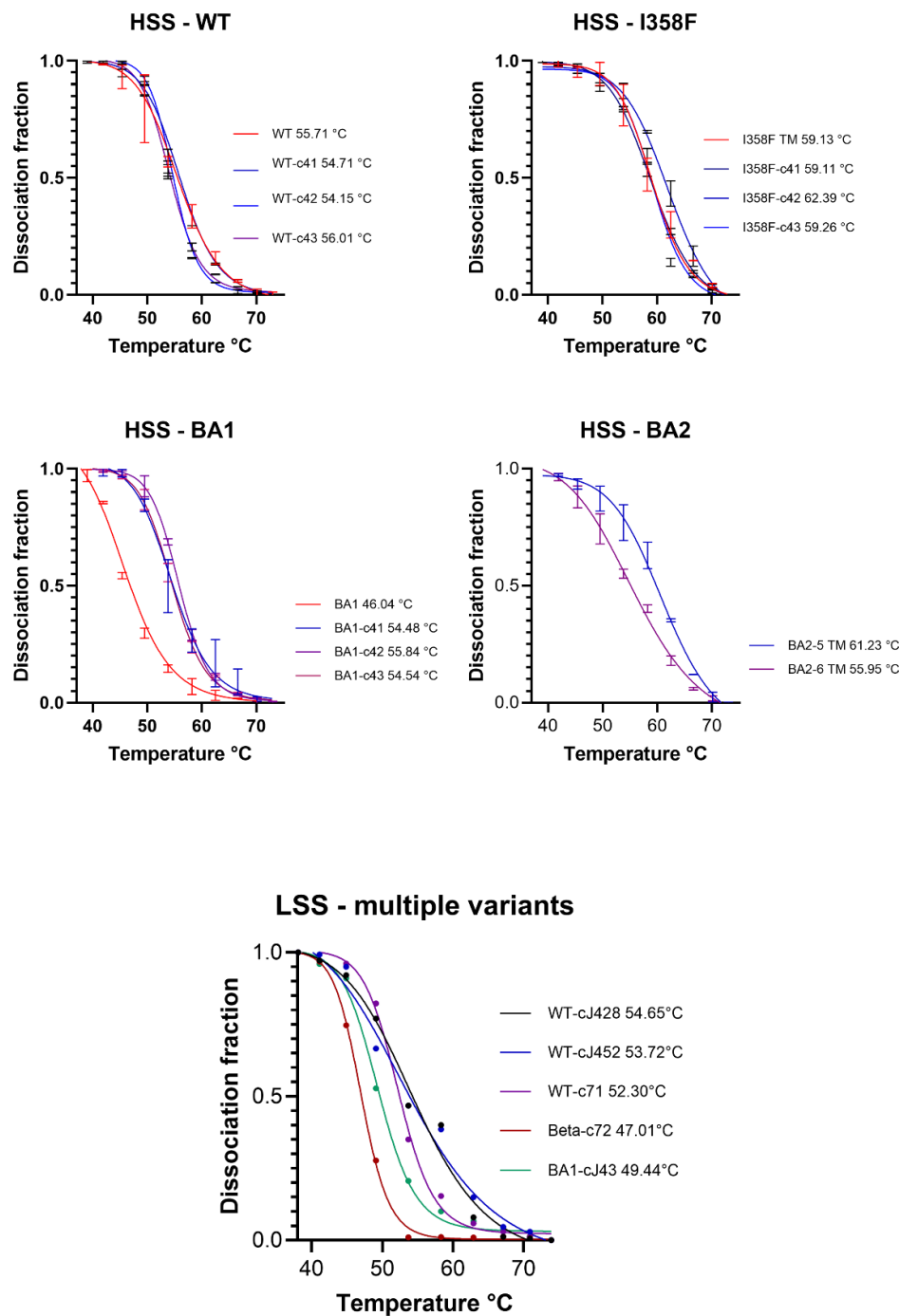

Figure S39 – Determination of the melting temperature (T_m) of RBD variants displayed on the yeast surface. Yeast cells expressing individual RBD clones on their surface were incubated at varying temperatures (38–72 °C) for 15 min to induce thermal denaturation. Following heat treatment, cells were cooled and incubated with fluorescently labeled ACE2 (10–20 nM) to assess the fraction of non-denaturated, binding-competent RBDs. After washing with PBSF, median fluorescence intensity values in the red channel were quantified by flow cytometry. Normalized values were fitted to a four-parameter logistic (4PL) sigmoidal curve using GraphPad Prism, from which T_m values were estimated.

Shoshany *et al.* (2026) Stringent Selection Drives Convergence Toward Omicron-like SARS-CoV-2 Receptor-Binding Motifs

SARS-CoV-1 library	clone	WT (CoV)
1	1	C P F S E V F N A T I F P S V Y A W E R K K I S N C V A D Y S V L N S T F S T F A C Y G V S A T L N D L C F S N V Y A D S F V K G D D V Q I A P G Q T G V I A D Y N Y L P D D F M G C V L A W N T N I D A T S T G N Y K Y R L R G K L R P F E D I S N V F S P D G K C T P A L N C W P L N D Y G F T T T G I G Y Q P R V V L S F
1	2	C P F S E V F N A T I F P S V Y A W E R K K I S N C V A D Y S V L N S T F S T F A C Y G V S A T L N D L C F S N V Y A D S F V K G D D V Q I A P G Q T G V I A D Y N Y L P D D F M G C V L A W N T N I D A T S T G N Y K Y R L R G K L R P F E D I S N V F S P D G K C T P A L N C W P L N D Y G F T T T G I G Y Q P R V V L S F
1	3	C P F S E V F N A T I F P S V Y A W E R K K I S N C V A D Y S V L N S T F S T F A C Y G V S A T L N D L C F S N V Y A D S F V K G D D V Q I A P G Q T G V I A D Y N Y L P D D F M G C V L A W N T N I D A T S T G N Y K Y R L R G K L R P F E D I S N V F S P D G K C T P A L N C W P L N D Y G F T T T G I G Y Q P R V V L S F
2	1	C P F S E V F N A T I F P S V Y A W E R K K I S N C V A D Y S V L N S T F S T F A C Y G V S A T L N D L C F S N V Y A D S F V K G D D V Q I A P G Q T G V I A D Y N Y L P D D F M G C V L A W N T N I D A T S T G N Y K Y R L R G K L R P F E D I S N V F S P D G K C T P A L N C W P L N D Y G F T T T G I G Y Q P R V V L S F
2	2	C P F S E V F N A T I F P S V Y A W E R K K I S N C V A D Y S V L N S T F S T F A C Y G V S A T L N D L C F S N V Y A D S F V K G D D V Q I A P G Q T G V I A D Y N Y L P D D F M G C V L A W N T N I D A T S T G N Y K Y R L R G K L R P F E D I S N V F S P D G K C T P A L N C W P L N D Y G F T T T G I G Y Q P R V V L S F
2	3	C P F S E V F N A T I F P S V Y A W E R K K I S N C V A D Y S V L N S T F S T F A C Y G V S A T L N D L C F S N V Y A D S F V K G D D V Q I A P G Q T G V I A D Y N Y L P D D F M G C V L A W N T N I D A T S T G N Y K Y R L R G K L R P F E D I S N V F S P D G K C T P A L N C W P L N D Y G F T T T G I G Y Q P R V V L S F
3	1	C P F S E V F N A T I F P S V Y A W E R K K I S N C V A D Y S V L N S T F S T F A C Y G V S A T L N D L C F S N V Y A D S F V K G D D V Q I A P G Q T G V I A D Y N Y L P D D F M G C V L A W N T N I D A T S T G N Y K Y R L R G K L R P F E D I S N V F S P D G K C T P A L N C W P L N D Y G F T T T G I G Y Q P R V V L S F
3	2	C P F S E V F N A T I F P S V Y A W E R K K I S N C V A D Y S V L N S T F S T F A C Y G V S A T L N D L C F S N V Y A D S F V K G D D V Q I A P G Q T G V I A D Y N Y L P D D F M G C V L A W N T N I D A T S T G N Y K Y R L R G K L R P F E D I S N V F S P D G K C T P A L N C W P L N D Y G F T T T G I G Y Q P R V V L S F
3	3	C P F S E V F N A T I F P S V Y A W E R K K I S N C V A D Y S V L N S T F S T F A C Y G V S A T L N D L C F S N V Y A D S F V K G D D V Q I A P G Q T G V I A D Y N Y L P D D F M G C V L A W N T N I D A T S T G N Y K Y R L R G K L R P F E D I S N V F S P D G K C T P A L N C W P L N D Y G F T T T G I G Y Q P R V V L S F
4	1	C P F S E V F N A T I F P S V Y A W E R K K I S N C V A D Y S V L N S T F S T F A C Y G V S A T L N D L C F S N V Y A D S F V K G D D V Q I A P G Q T G V I A D Y N Y L P D D F M G C V L A W N T N I D A T S T G N Y K Y R L R G K L R P F E D I S N V F S P D G K C T P A L N C W P L N D Y G F T T T G I G Y Q P R V V L S F
4	2	C P F S E V F N A T I F P S V Y A W E R K K I S N C V A D Y S V L N S T F S T F A C Y G V S A T L N D L C F S N V Y A D S F V K G D D V Q I A P G Q T G V I A D Y N Y L P D D F M G C V L A W N T N I D A T S T G N Y K Y R L R G K L R P F E D I S N V F S P D G K C T P A L N C W P L N D Y G F T T T G I G Y Q P R V V L S F
4	3	C P F S E V F N A T I F P S V Y A W E R K K I S N C V A D Y S V L N S T F S T F A C Y G V S A T L N D L C F S N V Y A D S F V K G D D V Q I A P G Q T G V I A D Y N Y L P D D F M G C V L A W N T N I D A T S T G N Y K Y R L R G K L R P F E D I S N V F S P D G K C T P A L N C W P L N D Y G F T T T G I G Y Q P R V V L S F
5	1	C P F S E V F N A T I F P S V Y A W E R K K I S N C V A D Y S V L N S T F S T F A C Y G V S A T L N D L C F S N V Y A D S F V K G D D V Q I A P G Q T G V I A D Y N Y L P D D F M G C V L A W N T N I D A T S T G N Y K Y R L R G K L R P F E D I S N V F S P D G K C T P A L N C W P L N D Y G F T T T G I G Y Q P R V V L S F
5	2	C P F S E V F N A T I F P S V Y A W E R K K I S N C V A D Y S V L N S T F S T F A C Y G V S A T L N D L C F S N V Y A D S F V K G D D V Q I A P G Q T G V I A D Y N Y L P D D F M G C V L A W N T N I D A T S T G N Y K Y R L R G K L R P F E D I S N V F S P D G K C T P A L N C W P L N D Y G F T T T G I G Y Q P R V V L S F
5	3	C P F S E V F N A T I F P S V Y A W E R K K I S N C V A D Y S V L N S T F S T F A C Y G V S A T L N D L C F S N V Y A D S F V K G D D V Q I A P G Q T G V I A D Y N Y L P D D F M G C V L A W N T N I D A T S T G N Y K Y R L R G K L R P F E D I S N V F S P D G K C T P A L N C W P L N D Y G F T T T G I G Y Q P R V V L S F

Fig S40 – Translated Sanger sequencing results for randomly selected SARS-CoV-1 affinity maturation clones. Residues identical to the SARS-CoV-1 WT sequence are shown in gray, whereas mutated amino acids are indicated in white.

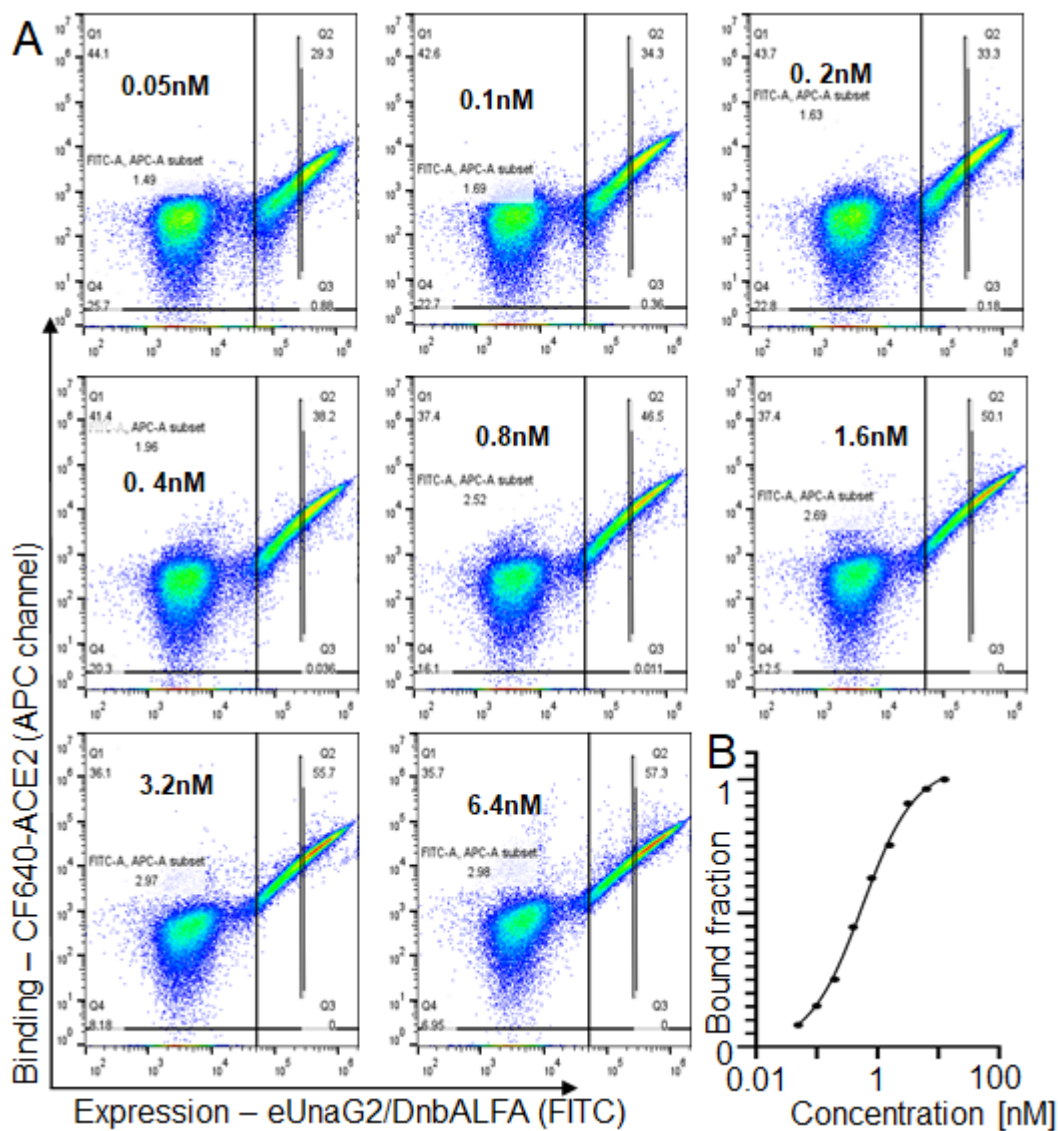

Fig. S41 – Gating strategy for yeast display-based binding affinity measurements. (A) A narrow vertical subset/subpopulation was defined to normalize the expression rates (FITC-A signal intensity, x-axis 10^0 - 10^6) across samples. Specific binding (APC-A signal intensity, y-axis 10^0 - 10^7) for each measurement was determined by subtracting the negative binding (Q1 median APC-A intensity) from the positive binding (Q2>subset median APC-A intensity). (B) Specific binding values at each ACE-2 concentration were fitted using GraphPad (Methods).

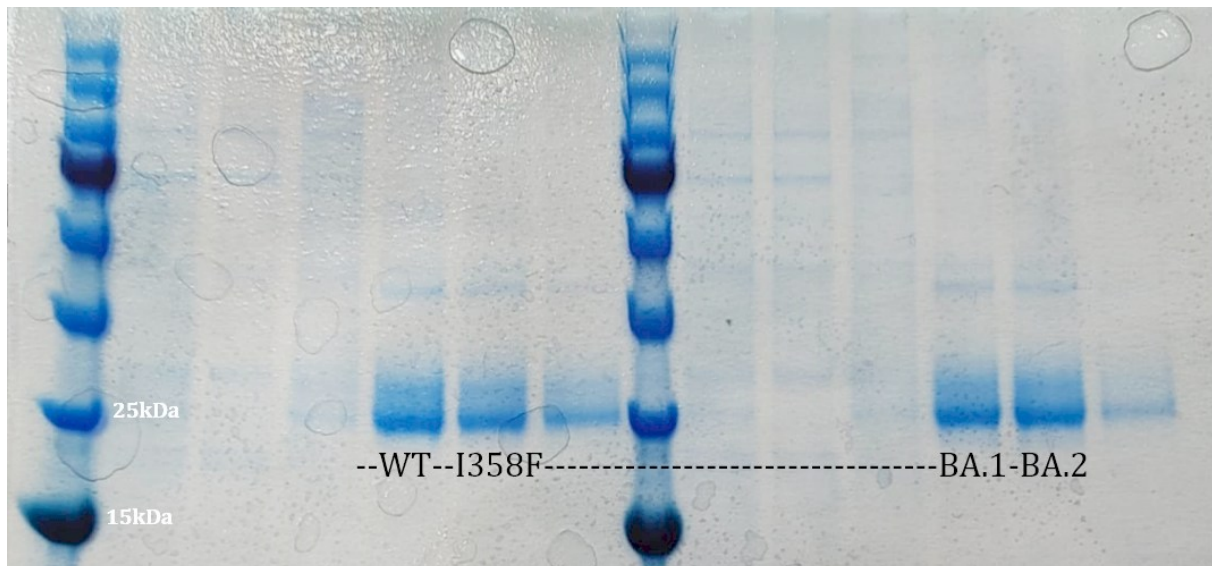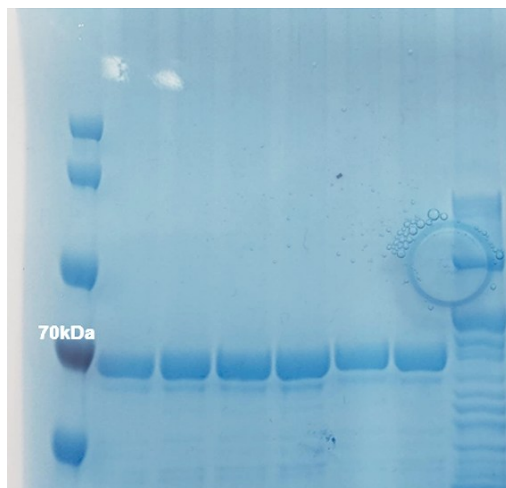

ACE2D-NiNTA-bdSUMO-cleavage

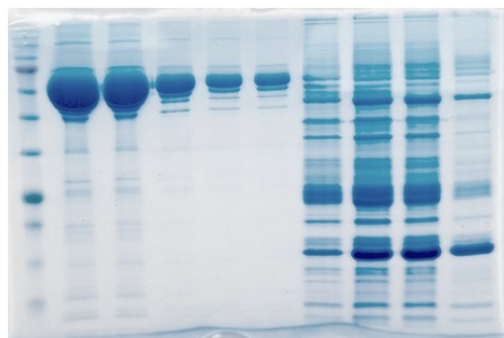

Sumo protease cleavage
fractions
(concentrated and
subjected to Superdex 75
16/600)

Imidazole elution fractions
after protease cleavage
(NiNTA resin bound fraction
control)

ACE2D-Superdex 75 16/600

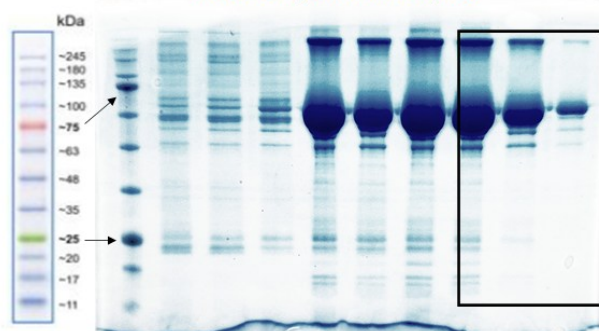

Used in subsequent
Experiments

Source data for Fig. S2 – Uncropped gels and full purification process for ACE2D.
