# Supplementary material for: Stringent selection drives convergence toward omicron-like SARS-CoV-2 receptor-binding motifs
Source: Nat Commun. 2026 Apr 25;17:5712. doi: 10.1038/s41467-026-72312-z (PMC13324855; doi:10.1038/s41467-026-72312-z)
Supplement: Supplementary file 3 — Description of Additional Supplementary Files [file 41467_2026_72312_MOESM3_ESM.pdf]

Description of Additional Supplementary Files

File Name: Supplementary Data 1
Description: This document contains a list of primers and their sequences used in the study.
