# Supplementary material for: Stringent selection drives convergence toward omicron-like SARS-CoV-2 receptor-binding motifs
Source: Nat Commun. 2026 Apr 25;17:5712. doi: 10.1038/s41467-026-72312-z (PMC13324855; doi:10.1038/s41467-026-72312-z)
Supplement: Supplementary file 5 — Reporting Summary [file 41467_2026_72312_MOESM5_ESM.pdf]

nature portfolio

Corresponding author(s): Gideon Schreiber and Jiri Zahradnik

Last updated by author(s): Feb 12, 2026

Reporting Summary

Nature Portfolio wishes to improve the reproducibility of the work that we publish. This form provides structure for consistency and transparency in reporting. For further information on Nature Portfolio policies, see our Editorial Policies and the Editorial Policy Checklist.

Statistics

For all statistical analyses, confirm that the following items are present in the figure legend, table legend, main text, or Methods section.

n/a	Confirmed	
☐	☒	The exact sample size (*n*) for each experimental group/condition, given as a discrete number and unit of measurement
☐	☒	A statement on whether measurements were taken from distinct samples or whether the same sample was measured repeatedly
☒	☐	The statistical test(s) used AND whether they are one- or two-sided<br>*Only common tests should be described solely by name; describe more complex techniques in the Methods section.*
☒	☐	A description of all covariates tested
☒	☐	A description of any assumptions or corrections, such as tests of normality and adjustment for multiple comparisons
☐	☒	A full description of the statistical parameters including central tendency (e.g. means) or other basic estimates (e.g. regression coefficient) AND variation (e.g. standard deviation) or associated estimates of uncertainty (e.g. confidence intervals)
☒	☐	For null hypothesis testing, the test statistic (e.g. *F*, *t*, *r*) with confidence intervals, effect sizes, degrees of freedom and *P* value noted<br>*Give P values as exact values whenever suitable.*
☒	☐	For Bayesian analysis, information on the choice of priors and Markov chain Monte Carlo settings
☒	☐	For hierarchical and complex designs, identification of the appropriate level for tests and full reporting of outcomes
☒	☐	Estimates of effect sizes (e.g. Cohen's *d*, Pearson's *r*), indicating how they were calculated

Our web collection on statistics for biologists contains articles on many of the points above.

Software and code

Policy information about availability of computer code

Data collection	The data were acquired by Illumina Nextseq 2000, with P1 flowcell, configuration 300/8/8/300 and software BCL Convert verze 4.2.7.
Data analysis	Demultiplexing of Illumina sequencing data was done using the standard Illumina bcl2fastq2 tool available as support resource (https://www.illumina.com/,v. 2.20). Alignment files were analyzed by “gofasta sam to MultiAlign” command (https://github.com/virus-evolution/gofasta). For the follow-up analysis we developed in Python two programs which are now available at https://github.com/host-patho-evo/mutation_scatter_plot.

For manuscripts utilizing custom algorithms or software that are central to the research but not yet described in published literature, software must be made available to editors and reviewers. We strongly encourage code deposition in a community repository (e.g. GitHub). See the Nature Portfolio guidelines for submitting code & software for further information.

Data

Policy information about availability of data

All manuscripts must include a data availability statement. This statement should provide the following information, where applicable:

- Accession codes, unique identifiers, or web links for publicly available datasets
- A description of any restrictions on data availability
- For clinical datasets or third party data, please ensure that the statement adheres to our policy

Source data for all main figures are provided in the accompanying Source Data files. All other data supporting the findings of this study on SARS-CoV-2 evolution are

available within the article, in the Supplementary Information, and via the EU Open Research Repository Zenodo under DOI: 10.5281/zenodo.19475241 including the list of GISAID sequence headers used in evolutionary comparisons. Full sequences and their metadata are available through GISAID (https://gisaid.org/). Data related to in vitro evolution of SARS-CoV-1 are subject to restricted access due to considerations of potential dual-use research of concern (DURC). Access can be granted upon request to the corresponding author, Dr. Jiri Zahradnik.

Research involving human participants, their data, or biological material

Policy information about studies with human participants or human data. See also policy information about sex, gender (identity/presentation), and sexual orientation and race, ethnicity and racism.

Reporting on sex and gender	*Use the terms sex (biological attribute) and gender (shaped by social and cultural circumstances) carefully in order to avoid confusing both terms. Indicate if findings apply to only one sex or gender; describe whether sex and gender were considered in study design; whether sex and/or gender was determined based on self-reporting or assigned and methods used. Provide in the source data disaggregated sex and gender data, where this information has been collected, and if consent has been obtained for sharing of individual-level data; provide overall numbers in this Reporting Summary. Please state if this information has not been collected. Report sex- and gender-based analyses where performed, justify reasons for lack of sex- and gender-based analysis.*
Reporting on race, ethnicity, or other socially relevant groupings	*Please specify the socially constructed or socially relevant categorization variable(s) used in your manuscript and explain why they were used. Please note that such variables should not be used as proxies for other socially constructed/relevant variables (for example, race or ethnicity should not be used as a proxy for socioeconomic status). Provide clear definitions of the relevant terms used, how they were provided (by the participants/respondents, the researchers, or third parties), and the method(s) used to classify people into the different categories (e.g. self-report, census or administrative data, social media data, etc.) Please provide details about how you controlled for confounding variables in your analyses.*
Population characteristics	*Describe the covariate-relevant population characteristics of the human research participants (e.g. age, genotypic information, past and current diagnosis and treatment categories). If you filled out the behavioural & social sciences study design questions and have nothing to add here, write "See above."*
Recruitment	*Describe how participants were recruited. Outline any potential self-selection bias or other biases that may be present and how these are likely to impact results.*
Ethics oversight	*Identify the organization(s) that approved the study protocol.*

Note that full information on the approval of the study protocol must also be provided in the manuscript.

Field-specific reporting

Please select the one below that is the best fit for your research. If you are not sure, read the appropriate sections before making your selection.

☒ Life sciences ☐ Behavioural & social sciences ☐ Ecological, evolutionary & environmental sciences

For a reference copy of the document with all sections, see nature.com/documents/nr-reporting-summary-flat.pdf

Life sciences study design

All studies must disclose on these points even when the disclosure is negative.

Sample size	*Describe how sample size was determined, detailing any statistical methods used to predetermine sample size OR if no sample-size calculation was performed, describe how sample sizes were chosen and provide a rationale for why these sample sizes are sufficient.*
Data exclusions	*Describe any data exclusions. If no data were excluded from the analyses, state so OR if data were excluded, describe the exclusions and the rationale behind them, indicating whether exclusion criteria were pre-established.*
Replication	The number of replicates is clearly identified when relevant. There was a high level of reproducibility among yeast display expression levels and binding affinities.
Randomization	*Describe how samples/organisms/participants were allocated into experimental groups. If allocation was not random, describe how covariates were controlled OR if this is not relevant to your study, explain why.*
Blinding	*Describe whether the investigators were blinded to group allocation during data collection and/or analysis. If blinding was not possible, describe why OR explain why blinding was not relevant to your study.*

Reporting for specific materials, systems and methods

We require information from authors about some types of materials, experimental systems and methods used in many studies. Here, indicate whether each material, system or method listed is relevant to your study. If you are not sure if a list item applies to your research, read the appropriate section before selecting a response.

Materials & experimental systems

n/a	Involved in the study
☒	☐ Antibodies
☐	☒ Eukaryotic cell lines
☒	☐ Palaeontology and archaeology
☒	☐ Animals and other organisms
☒	☐ Clinical data
☐	☒ Dual use research of concern
☒	☐ Plants

Methods

n/a	Involved in the study
☒	☐ ChIP-seq
☐	☒ Flow cytometry
☒	☐ MRI-based neuroimaging

Eukaryotic cell lines

Policy information about cell lines and Sex and Gender in Research

Cell line source(s)	S.cerevisiae EBY100 (https://www.atcc.org/products/mya-4941), HEK293F cells (Weizmann Institute of Science cell line repository, Cytion no:300260)
Authentication	Provided by the supplier. Yeast display efficience was tested with positive control plasmids - doi.:10.1021/acssynbio.1c00395. HEK293F cells were used for protein production only and cell authentication is therefore not required.
Mycoplasma contamination	Not tested in yeast. HEK293F cell line was negatively tested for mycoplasma contamination by Hy-mycoplasma detection PCR kit (HyLabs, Ky 50341)
Commonly misidentified lines (See ICLAC register)	*Name any commonly misidentified cell lines used in the study and provide a rationale for their use.*

Dual use research of concern

Policy information about dual use research of concern

Hazards

Could the accidental, deliberate or reckless misuse of agents or technologies generated in the work, or the application of information presented in the manuscript, pose a threat to:

No	Yes
☐	☒ Public health
☒	☐ National security
☒	☐ Crops and/or livestock
☒	☐ Ecosystems
☒	☐ Any other significant area

Hazards	This study presents affinity selections of the receptor-binding domains (RBDs) of SARS-CoV-2 and SARS-CoV-1. While some of the identified mutations may theoretically enhance viral infectivity if deliberately introduced into a live virus, this was not the subject of the research. The RBD proteins used in this study are non-infectious and pose no biological risk. On the contrary, they can block viral entry by competitively inhibiting the ACE2 receptor interaction.Most of the SARS-CoV-2 mutations identified in this work have already been observed in natural viral populations. In the case of SARS-CoV-1, the study describes convergence on specific residues, suggesting that although these mutations increase RBD affinity, they are unlikely to confer escape from antibody recognition. The RBD represents only a small portion of the Spike protein, and the mutations described here are not expected to affect immune responses targeting other regions of the Spike protein or other viral proteins.

For examples of agents subject to oversight, see the United States Government Policy for Institutional Oversight of Life Sciences Dual Use Research of Concern.

Experiments of concern

Does the work involve any of these experiments of concern:

No	Yes	
☒	☐	Demonstrate how to render a vaccine ineffective
☒	☐	Confer resistance to therapeutically useful antibiotics or antiviral agents
☒	☐	Enhance the virulence of a pathogen or render a nonpathogen virulent
☐	☒	Increase transmissibility of a pathogen
☒	☐	Alter the host range of a pathogen
☒	☐	Enable evasion of diagnostic/detection modalities
☒	☐	Enable the weaponization of a biological agent or toxin
☒	☐	Any other potentially harmful combination of experiments and agents

Precautions and benefits

Biosecurity precautions	All experiments described in this study were conducted strictly under biosafety level 1 or 2 conditions. The entirely in vitro nature of the work did not require any special biosafety precautions, and the recombinant proteins used in the study pose no biological risk.
Biosecurity oversight	Biosafety (BSL) and biosecurity oversight were provided by the Centre for Experimental Biomodels, First Faculty of Medicine, which is formally authorized to perform this function.
Benefits	The benefit of this work lies in advancing our understanding of the evolutionary pressures shaping the receptor-binding domain (RBD) of SARS-CoV-2. To demonstrate the broader applicability of our approach, we applied the same in vitro evolution strategy to the RBD of SARS-CoV-1. This enabled us to identify mutations that could plausibly arise if SARS-CoV-1 were to re-emerge and adapt to the human host. We believe that the predictive value of this method—particularly its potential to inform the development of broadly protective vaccines and to anticipate zoonotic threats—outweighs the theoretical risks associated with experimentally identifying such mutations.
Communication benefits	The communication of these results is of high importance, as the findings are applicable beyond SARS-CoV-1 and SARS-CoV-2. They may serve as a foundation for the development of broadly protective vaccines, as well as for the monitoring and risk assessment of emerging zoonotic viruses.

Plants

Seed stocks	*Report on the source of all seed stocks or other plant material used. If applicable, state the seed stock centre and catalogue number. If plant specimens were collected from the field, describe the collection location, date and sampling procedures.*
Novel plant genotypes	*Describe the methods by which all novel plant genotypes were produced. This includes those generated by transgenic approaches, gene editing, chemical/radiation-based mutagenesis and hybridization. For transgenic lines, describe the transformation method, the number of independent lines analyzed and the generation upon which experiments were performed. For gene-edited lines, describe the editor used, the endogenous sequence targeted for editing, the targeting guide RNA sequence (if applicable) and how the editor was applied.*
Authentication	*Describe any authentication procedures for each seed stock used or novel genotype generated. Describe any experiments used to assess the effect of a mutation and, where applicable, how potential secondary effects (e.g. second site T-DNA insertions, mosiacism, off-target gene editing) were examined.*

Flow Cytometry

Plots

Confirm that:

☒ The axis labels state the marker and fluorochrome used (e.g. CD4-FITC).

☒ The axis scales are clearly visible. Include numbers along axes only for bottom left plot of group (a 'group' is an analysis of identical markers).

☒ All plots are contour plots with outliers or pseudocolor plots.

☒ A numerical value for number of cells or percentage (with statistics) is provided.

Methodology

Sample preparation	Sample preparation is described in detail in the Method section. S.cerevisiae EBY100 cells expressing the RBD variant/s were washed by an ice-cold PBSB buffer (PBS with 1 g/L BSA) and incubated with various concentrations of CF-640 labeled ACE2 protein. The sample volume was adjusted (1 – 100 ml) to avoid the ligand depletion when needed as well as an equilibrium incubation time (1 – 12 h). The cells were subsequently washed (PBSF), passed through the cell strainer, and analyzed by

	cytometry. Gating strategy is shown in detail in Supporting information part S2, figure S1.
Instrument	The sorting experiments were performed on S3e Cell Sorter (BioRad, USA, serial no 777BR2079). The binding data were acquired on S3e Cell Sorter (BioRad, USA) or on CytoFLEX cytometer (Beckman Coulter Life Sciences, USA, serial 9678204)
Software	Analyses were done by ProSort™ Software (v. 1.6) or by CytExpert (v. 2.4)
Cell population abundance	*Describe the abundance of the relevant cell populations within post-sort fractions, providing details on the purity of the samples and how it was determined.*
Gating strategy	Gatin strategies are described in Supporting information part S2, Figure S1.

☒ Tick this box to confirm that a figure exemplifying the gating strategy is provided in the Supplementary Information.
