# Supplementary material for: Stringent selection drives convergence toward omicron-like SARS-CoV-2 receptor-binding motifs
Source: Nat Commun. 2026 Apr 25;17:5712. doi: 10.1038/s41467-026-72312-z (PMC13324855; doi:10.1038/s41467-026-72312-z)
Supplement: Supplementary file 2 — Peer Review file [file 41467_2026_72312_MOESM2_ESM.pdf]

nature portfolio

Peer Review File

Stringent Selection Drives Convergence Toward Omicron-like SARS-CoV-2 Receptor-Binding Motifs

Corresponding Author: Dr Jiří Zahradník

This file contains all reviewer reports in order by version, followed by all author rebuttals in order by version.

Version 0:

Reviewer comments:

Reviewer #1

(Remarks to the Author)
Shoshany et al. perform yeast display-based in vitro evolution experiments on SARS-CoV-2 RBD using different ACE2 selection pressures. These experiments identify mutations that get selected for ACE2 binding and RBD stability in several different SARS-CoV-2 variant backgrounds. They find that in the Wuhan-1 background many mutations that get fixed in experiments are the ones that occurred when the Omicron variant emerged. While the results of this study make some sense and seem reasonable, providing feedback on results is hard because I found understanding the actual method and the exact way the selection was performed really difficult due to a lack of clear general explanation of the system used.

Authors need to describe the RBD yeast display system they use much more thoroughly. Currently the results section mostly only mentions additional improvements the researchers made on top of the previous system they have published and the reader is expected to look up their previous paper to understand how the system works.
Point one applies to describing the the improvements made to the system as well, e.g. authors should not expect readers to know how SpyCatcher works, or have to read through methods section to find out what eUnaG2 and DnbALFA are, particularly given that these are not “standard” techniques and
reporters. Authors also need to explain what is the advantage of using a different reporter plasmid for each selection round, it is not obvious.
Fig. 1 legend needs to be more descriptive including defining all the acronyms in it, none of which are explained.
Fig 1C compared HSS and LSS selection strategies, but based on what authors say in the text random mutagenesis is performed between each selection round it would be good to show that in the figure as well.
Fig 1 would also benefit from showing a comparison between mutations unique to each variant background used to make libraries relative to Wuhan-1.
Can authors explain why they use different DNA libraries for HSS and LSS (both plasmid libraries and regions that are mutagenised differ) instead of starting with the same library given that they compare the outcomes of these selections
Numbers on the x-axis of Fig. 3C-D are not legible
Seems that authors used NGS to analyse original library composition but then used only Sanger sequencing to determine mutations that get selected after each selection round? The number of clones that are sequenced after each selection round seems to differ and vary between libraries, why is that how was the number of clones to be sequenced determined? If only Sanger sequencing was done instead of some form of deep sequencing does that mean the data likely underestimates lower frequency variants?
Authors say that in RBD-v48 library “increased affinity provided a “surplus” of affinity providing broader mutational tolerance, allowing the fixation of unique mutations such as A348P, A363P, K386E, N388D, N460S, I468V, V483A, and S514T identified by NGS”. I do not see where this data is shown.
I’m not sure that this figure reference is correct ”The display of RBD variants with and without this mutation revealed a striking difference, indicating its dramatic structural and functional impact (Fig. S1)”. Fig S1 shows gating strategy and Fig. S11 shows disappearance of S375F from BA.1, I’m not sure what the authors are referring to in tat sentence.
While authors say BLOSUM62 matrix is commonly used, I doubt the majority of people know what it is and this should be explained.
Authors should discuss some limitation of using RBD only system, e.g. we know ACE2 binding is affected by RBD up down movement, indeed S375F mutation that was selected against in this study is well described as mutation that leads to RBD more in the down position (https://doi.org/10.1038/s41467-022-32665-7, https://doi.org/10.1101/2021.12.21.473620).

(Remarks on code availability)
Code is available with detailed README. I did not try to rerun the analysis.

Reviewer #2

(Remarks to the Author)
This study employs an enhanced yeast surface display method to identify mutations in SARS-CoV-2 RBD/RBM that maintain or increase binding affinity for ACE2. Selections of mutants are performed under low (LSS) and high stringency (HSS) conditions for ACE2 binding. Under HSS conditions some mutations are more highly represented than under LSS conditions, and the authors report the HSS mutants to be higher affinity for ACE2. Many of these higher affinity HSS mutations are found in SARS-CoV-2 sequences in the GISAID database and in Omicron variants. The main findings of the study are that high stringency selection conditions favor rapid fixation of mutants for the selected phenotype, and that mutations selected for enhanced ACE2 binding are very common in SARS-CoV-2 Omicron variants.

The study is robust and well designed. The authors use a range of starting variants for their screens, in addition to Wt, and employ alternating selection plasmids to enhance reliability. The starting libraries are good quality. The data on HSS vs LSS fixation is convincing. Many of the mutations selected for highest ACE2 affinity binding are already known to enhance ACE2 binding affinity, supporting the validity of the selection protocol for ACE2 binding. Some of these HSS mutants are also directly assayed for ACE2 affinity, though this should be extended to a wider range of mutants.

It is known that Omicron variants exhibit increased affinity for ACE2 and possess mutations that increase ACE2 affinity. The present study confirms that mutations selected for increased ACE2 affinity are commonly found in Omicron variants. Whilst the study provides a very good demonstration of the power of their yeast display method to identify mutations leading to increased ACE2 affinity, the key findings of the study are not in themselves particularly novel.

The manuscript promotes the idea that ACE2 affinity is the main driver of Omicron evolution. However, this has not been tested or proven in the current study. The finding that mutations selected for increased ACE2 affinity are present in SARS-CoV-2 is not the same as showing ACE2 affinity is the main driver of Omicron evolution, rather it confirms ACE2 affinity as an important component of Omicron phenotype.

Other points

Figure 4 - affinity differences between LSS clones vs HSS clones are not clear as LSS clones are shown on a more compressed X-axis than HSS clones, the same dimension axes for panels A and C are required to compare clones in this figure.

The effects of HSS and LSS on clone affinity (Figure 4C and Table S7) should be more comprehensively and accurately described. The average affinity gain of the LSS clones is actually higher than the HSS clones (Table S7). Measurement of affinity gain in more LSS and HSS clones would allow a more robust conclusion about the effects of LSS vs HSS on affinity to be drawn.

Some of the figures could be clearer. Figure S13 should have the mutations introduced to create the variants BA.1 and BA.2 colored yellow for those variants.

In Figure S5 it is not immediately clear which mutations have been selected by HSS and which are present because they were in the starting variant, e.g. N440K is already present in BA.1 and BA.2. This should be clarified by use of a different color for such mutations. The superscript in the table legend should also be checked.

For analysis of convergence between in vitro and virus evolution it would it be valuable to compare frequencies of co-occurring mutations in the GISAID database with mutation co-occurrence in clones from HSS.

(Remarks on code availability)

Reviewer #3

(Remarks to the Author)
This work builds upon previous work from the same group in 2021 (Zahradnik et al, 2021) where they performed in vitro evolution and affinity maturation of the SARS-CoV-2 RBD towards ACE2 and ended up with a mutant RBD with 600-fold increase in binding affinity. Some of the mutations that they evolved were also observed in Omicron. That study had certain limitations, the codons were optimized for expression in Saccharomyces cerevisiae for yeast display and in vitro evolution included a pre-equilibrium selection step favoring faster on-rates, which may not align with natural viral evolution. Thus, they redesigned/improved their study in this paper.

This paper is actually split in two major parts. In the first one (which is most of the paper), the authors study how in vitro evolution can be used to identify SARS-CoV-2 mutations that increase binding affinity to ACE2, while maintaining structural integrity. They compare their results with mutations that occurred in real SARS-CoV-2 strains. This is actually used as a

proof of concept to study how ACE2 binding can be increased in SARS-CoV-1 related sequences, in the second part of the paper (this is only a small section at the end of results). They identified Y442S as sufficient to close the binding affinity gap between SARS-CoV-1 and SARS-CoV-2. This is a very important finding. At the same time, it's actually very close to a gain of function experiment on how to significantly increase the binding affinity of a group of Sarbecoviruses (the SARS1 group) that have much lower infectivity at this time, but significantly higher mortality. SARS-CoV-1 had a mortality rate of ~10% whereas SARS-CoV-2, at the begining had a mortality rate bellow 1% and this further decreased. Basically, this experimental evolution framework is mostly useful for identifying mutations that will allow a virus to break the species barrier and start infecting successfully humans. It is not optimized for capturing the subsequent evolutionary trajectory of the virus, that will need to evade immune response, re-infect and also change tissue tropism in order to facilitate more easy spread (preferentially moving to the upper respiratory system), in the next phases of an epidemic. In my opinion, the most important finding of this work is how easy it actually is for a member of the SARS-CoV-1 evolutionary group to significantly increase its infectivity and start a new epidemic (like that of 2002-2004 SARS outbreak) that at the early stages may also have a significantly increased lethality (possibly ten times higher than SARS-CoV-2 had at the begining). The implications are very significant. The scientific community needs to remain vigilant and continue research on Coronaviruses. Both in terms of monitoring and in terms of rapidly developing safe and effective vaccine technologies. In my opinion, RNA vaccines are the way forward, for rapid development, in order to combat any new epidemic, but safety issues need to be addressed as well. And other more conserved regions for a pan-Sarbecovirus vaccine need to be further investigated. At the same time, it shows that a new epidemic with a SARS-1 related sarbecovirus may easily evolve in nature, without the need for a lab-leak. Although, given the level of natural immunization that the human population has aquired in the last 5 years for SARS-CoV-2, it is possible that a new SARS-CoV-1 related epidemic may not be as bad (assuming significant cross-reaction). Furthermore, this work also shows how close the world was in 2002-2004 for SARS to evolve into a much more transmissible virus and generate a pandemic like that of 2019. The immediate response of the people that were involved in containing the 2002-2004 SARS outbreak was crucial in averting a catastrophic pandemic then. Thus, this highlights the importance of rapid response, collaboration, openess, sharing of information and coordinated action at the international level. My recommendation is to significantly reformat the paper accordingingly, in order to properly discuss all the above very significant findings and their even more important implications.

The authors also need to discuss further the weaknesses of this technology in terms of predicting the next phases of an epidemic. The fitness of the virus is a combination of increased transmissibility and immune evasion, while still retaining structural integrity. Increased transmissibility is in turn a combination of better binding to ACE2 and tissue tropism to the upper respiratory system, that allows it to spread more easily. While this experimental/in-vitro evolution setting can capture/explain the binding of the virus to ACE2, it does not account for tissue tropism and for immune evasion (see and discuss DOI: 10.3390/v14040707). At the early stages of the pandemic, where a large portion of the human population was still immunologically naïve, increased binding to the human ACE2 ortholog was very important. Nevertheless, in later stages, immune evasion and increased infectivity via the upper respiratory became more important. For example, almost all VoCs had not only increased binding to ACE2, but also increased immune escape and even increased re-infection rates. More specifically, the Omicron lineage demonstrated a re-infection rate 5.4 times higher than that of the already highly infectious Delta lineage. However, this increase in infectivity is not only due to higher affinity of the Spike RBD for the human ACE2 receptor. Pseudovirus assays demonstrated that the Omicron's RBD binding affinity for the human ACE2 receptor is 2.4 times higher than that of the Wuhan-Hu-1 strain (see DOI:10.1038/s41586-021-04386-2), which is lower than that of Alpha (6.2 times higher than Wuhan-Hu-1), similar to that of Beta (2.4 times higher than Wuhan-Hu-1), and higher than that of Delta (1.2 times higher than Wuhan-Hu-1). The high infection and re-infection rate of Omicron might be attributed, at least in part, to strong pressure to achieve antigenic shift, because it has emerged in an environment where many individuals already had been infected by other variants or were immunized to a large extent by Spike-targeting vaccines. The Omicron lineage could already escape 26 out of 29 monoclonal antibodies (see DOI:10.1038/s41586-021-04386-2) that target the Spike RBM. Furthermore, its tissue tropism changed from the lower to the upper respiratory system, and played a major role in increasing its overall infectivity compared to Delta. Thus, an in vitro system that focuses on optimizing the binding affinity of the Spike to ACE2 cannot account for the epistatic effects of immune-escape and tissue tropism switching.

In Introduction, the authors discuss the evolutionary rate of SARS-CoV-2. Evolutionary analyses estimate a mutation rate of $0.5 \times 10{-}3{-}1.1 \times 10{-}3$ substitutions/site/year, which translates to approximately 1.3–2.8 substitution/month for the entire genome. At the same time, the Spike region mutates almost 5 times faster than the other regions of SARS-CoV-2 (see and discuss DOI: 10.3390/v14040707). In addition, the same comparative study revealed that the amino acid substitutions of Spike were higher in the successful VoCs, compared to the other SARS-Cov-2 sequences.

The authors also have a very interesting section and discussion on the emergence of Omicron. It is noteworthy that each of the five major VoCs was not derived by a few extra mutation steps of another pre-existing VoC. This is possibly because steady-stepwise evolution may not be sufficient to achieve immune escape during the evolution of a pandemic where the vast majority of the population is no longer antigenically naïve (especially for the Spike ORF), but saltatory evolution may be necessary (see DOI:10.1073/pnas.1909031116). It is conceivable that "under-the-radar" lineage evolution may be occurring, involving a combination of host-switching and chronic infections of immune-compromised patients. Although the authors discuss the scenario involving immunocompromised patients, this alone cannot explain the intense localization of amino acid changes in the spike of Omicron. It possible that a more complex scenario of host switching accelerated changes at the Spike, followed by infection of an immunocompromised patient, that gave ample time for re-adaptation of that strain back to humans. See DOI: 10.1093/infdis/jiab622, and DOI: 10.3390/v14040707).

Several comparative studies have identified recurring mutations (homoplasies/convergent evolution) in successful VoCs (see DOI: 10.1016/j.meegid.2020.104351, DOI: 10.1073/pnas.2104241118 and DOI: 10.3390/v14040707). The authors need to discuss in more detail how many of these particular recurring mutations were identified in their experimental

evolution framework

Some minor corrections:

In vitro:Please italicize throughout the manuscript
In silico: Please italicize throughout the manuscript

Typo: Predominantly immune evasion associated mutations were not selected in vitro.

I would also recommend that the authors change the title of the paper, it doesn't read well as it is.

(Remarks on code availability)
Its fine.

Version 1:

Reviewer comments:

Reviewer #1

(Remarks to the Author)
I'm satisfied with authors responses and changes made.

(Remarks on code availability)

Reviewer #2

(Remarks to the Author)
The addition of data from Greaney et al, and comparative analysis performed by the authors, have strengthened the manuscript conclusions. Affinity differences between LSS and HSS library selections are now demonstrated in the repeated experiments and new data presented in Figure 4. The corrections made in the revised manuscript satisfactorily address the other points I raised.

(Remarks on code availability)

Reviewer #3

(Remarks to the Author)
The authors have adequately addressed all my comments and suggestions in the revised version.

(Remarks on code availability)
It is fine.

Open Access This Peer Review File is licensed under a Creative Commons Attribution 4.0 International License, which permits use, sharing, adaptation, distribution and reproduction in any medium or format, as long as you give appropriate credit to the original author(s) and the source, provide a link to the Creative Commons license, and indicate if changes were made.
In cases where reviewers are anonymous, credit should be given to 'Anonymous Referee' and the source.
The images or other third party material in this Peer Review File are included in the article's Creative Commons license, unless indicated otherwise in a credit line to the material. If material is not included in the article's Creative Commons license and your intended use is not permitted by statutory regulation or exceeds the permitted use, you will need to obtain permission directly from the copyright holder.
To view a copy of this license, visit https://creativecommons.org/licenses/by/4.0/

RESPONSES TO REVIEWER COMMENTS

Reviewer #1 (Remarks to the Author):

Shoshany et al. perform yeast display-based in vitro evolution experiments on SARS-CoV-2 RBD using different ACE2 selection pressures. These experiments identify mutations that get selected for ACE2 binding and RBD stability in several different SARS-CoV-2 variant backgrounds. They find that in the Wuhan-1 background many mutations that get fixed in experiments are the ones that occurred when the Omicron variant emerged. While the results of this study make some sense and seem reasonable, providing feedback on results is hard because I found understanding the actual method and the exact way the selection was performed really difficult due to a lack of clear general explanation of the system used. Authors need to describe the RBD yeast display system they use much more thoroughly. Currently the results section mostly only mentions additional improvements the researchers made on top of the previous system they have published and the reader is expected to look up their previous paper to understand how the system works. Point one applies to describing the improvements made to the system as well, e.g. authors should not expect readers to know how SpyCatcher works, or have to read through methods section to find out what eUnaG2 and DnbALFA are, particularly given that these are not "standard" techniques and reporters. Authors also need to explain what is the advantage of using a different reporter plasmid for each selection round, it is not obvious.

Response to Reviewer #1:
We want to thank the reviewer pointing out that the current methodology section, particularly of the yeast display system is not sufficiently clear. Accordingly, we have substantially revised the section "Experimental design and setup for parallel in vitro evolution" to ensure that readers can fully understand the methodology without prior familiarity with the enhanced yeast display system described in our previous work (lines 132-160, see revised Fig. 1 and accompanying text).

The orthogonal systems used to monitor surface expression strength are now described in detail in both the main text and the Supplementary Information. We also revised Fig. 1 and added a new panel (now Fig. 1C) illustrating the full mutagenesis–selection cycling workflow. In addition, we now explicitly explain the principles and roles of all key system components, including the SpyCatcher–SpyTag covalent display expression labeling, eUnaG2 green fluorescence reporter, and ALFA-tag binding nanobody DnbALFA detection module, which were previously only briefly mentioned.

We also expanded the rationale behind the use of different reporter plasmids in successive selection rounds (lines 132-160). The system improvements were primarily implemented to eliminate cross-contamination between parallel high-throughput FACS sorting experiments. Because sorting is inherently sensitive to minor cross-contaminations, employing distinct reporter plasmids for each evolution trajectory step allowed us to unambiguously identify and exclude potential contaminant sequences. Moreover, and shown in Fig. 1B and explained in the accompanying text, we used specific sequence barcodes for each evolved variant, showing that

no cross contamination occurred. Overall, this ensures that all mutations analyzed represent true outcomes of in vitro evolution rather than artifacts arising from library cross-mixing.

Fig. 1 legend needs to be more descriptive including defining all the acronyms in it, none of which are explained.

The Fig. 1 legend was completely revised and expanded to be fully self-explanatory. All acronyms are now defined, and each panel is described in sufficient detail. The figure itself was updated, with one new panel added and others modified for clarity.

Fig 1C compared HSS and LSS selection strategies, but based on what authors say in the text random mutagenesis is performed between each selection round it would be good to show that in the figure as well.

To clarify this, we added a new panel (now Fig. 1C), and the original panel (now 1D) was modified accordingly.

Fig 1 would also benefit from showing a comparison between mutations unique to each variant background used to make libraries relative to Wuhan-1.

As Figure 1 is already highly complex, we chose to highlight the mutations unique to each variant background in Fig. 3 in the sequence logo plots (panels 3C and 3D) and also in all sequencing-related figures in the Supplementary information (Fig. S8-S18), where they are shown in black. These mutations are now also explicitly mentioned in the main text

(lines 173–177): The starting RBDs originated from SARS-CoV-2 variants: Wuhan-Hu-1 (WT, and WT+I358F mutation), Alpha (mutation present N501Y), Beta (K417N, E484K, N501Y), RBD variant 48 (RBD-v48, Beta + Q498R mutation (Table S2) 25), Omicron BA.1 (RBD-v48 + G339D, S371L, S373P, S375F, N440K, G446S, S477N, T478K, E484A, Q493R, G496S, Y505H) and Omicron BA.2 (RBD-v48 + G339D, S371F, S373P, S375F, S376A, D405N, R408S, N440K, S477N, T478K, E484A, Q493R, Y505H).

Can authors explain why they use different DNA libraries for HSS and LSS (both plasmid libraries and regions that are mutagenised differ) instead of starting with the same library given that they compare the outcomes of these selections

We appreciate this important comment. We fully agree that addressing the potential impact of the initial library design and sequencing depth is essential for validating the conclusions of our study. Indeed, the two libraries (HSS and LSS) were originally constructed independently as part of distinct experimental plans. However, upon observing the emerging trends, we recognized their complementarity and combined them into a single, coherent research project. All steps after library selection, including next generation sequencing, further Sanger sequencing, binding affinity measurements and all steps of data analysis were done together for all selected libraries. Moreover, in the revised version we added new data and analysis to ensure that the observed differences between HSS and LSS *in vitro* evolution are not artifacts arising from library design or differences in sequencing depth.

1. Influence of library design:
HSS mutant libraries were established using the GeneMorph II Random Mutagenesis Kit, while LSS libraries were established using taq polymerase-based error prone mutagenesis procedures (Mn^{2+} method). Using Sanger and NGS sequencing we established that the number of mutations per *in vitro* evolution step were similar using both procedures (see method section). To ensure

complete coverage of all possible variants, the size of the yeast library was always at least two orders of magnitude greater than the theoretical number of single-nucleotide changes. The equivalence of the libraries is well illustrated by the comparison of the distribution of nucleotide sequence identities among them (newly included as Fig. S7).

The mutagenized region of the HSS library did not include mutations in residues 336–430, whereas this region was randomized in the LSS library. To assess the potential effect of this difference, we performed additional next-generation sequencing (NGS) of this region in the LSS library. The analysis revealed a pattern consistent with our Sanger sequencing results and confirmed the absence of clear convergence or fixation of residues characteristic of Omicron lineages (see mutation scatter plots under this paragraph (Figure 1) which are now included as Fig. S19 - S23 in the manuscript SI). Some positions, however, showed interesting diversification: for example, position 417 in the libraries derived from the Beta and Omicron variants, where mutations K417I and K417Y were recurrently fixed. Furthermore, we observed significant fixation of stabilizing mutations A348P and I358F, previously described to enhance RBD stability (ref. doi: 10.1038/s41564-021-00954-4 and 10.3389/fimmu.2021.765211). The NGS analysis also confirmed the loss of the S375F mutation in the BA.1 LSS library, as discussed in the manuscript. Collectively, these results demonstrate that the conclusions regarding the dominance of HSS and rapid fixation dynamics are not artifacts of library length or composition. All these new observations were added in the manuscript main text:

Lines 313-316: The K417N mutation (characteristic of the Beta and BA.1 variants) showed different behavior. It did not revert to the original residue and further diversified into several accepted substitutions, with K417I and K417Y emerging as the most prominent (Fig. S21-S23).

Lines 607-624: It should be noted that the use of the isolated RBD domain for *in vitro* evolution has limitations, as it may lack stabilizing contacts present in the full Spike trimer that can compensate for the destabilizing effect of the S375F substitution. Structural studies have shown that S375F favors the RBD adopting the “down” conformation [69-71], where substantially more intermolecular contacts are formed. Nevertheless, the overall stability of the Omicron Spike, at least in the initial inflection region, remains markedly reduced [72]. Sanger sequencing of individual LSS rounds revealed minimal fixation of mutations outside the RBM region (amino acids 336–430). In later rounds (≥3), partial fixation of the stabilizing mutations A348P and I358F was observed [25,73]. Overall, these results suggest that this portion of the RBD without destabilizing Omicron mutations has low impact on the acquisition of mutations within the RBM. Therefore, the presence of the S375F substitution, which is largely responsible for the Omicron phenotype [39], must be supported by complex epistatic effects underlying immune escape or tissue tropism switching. These effects likely confer a positive fitness advantage but cannot be directly recapitulated in our experimental system.

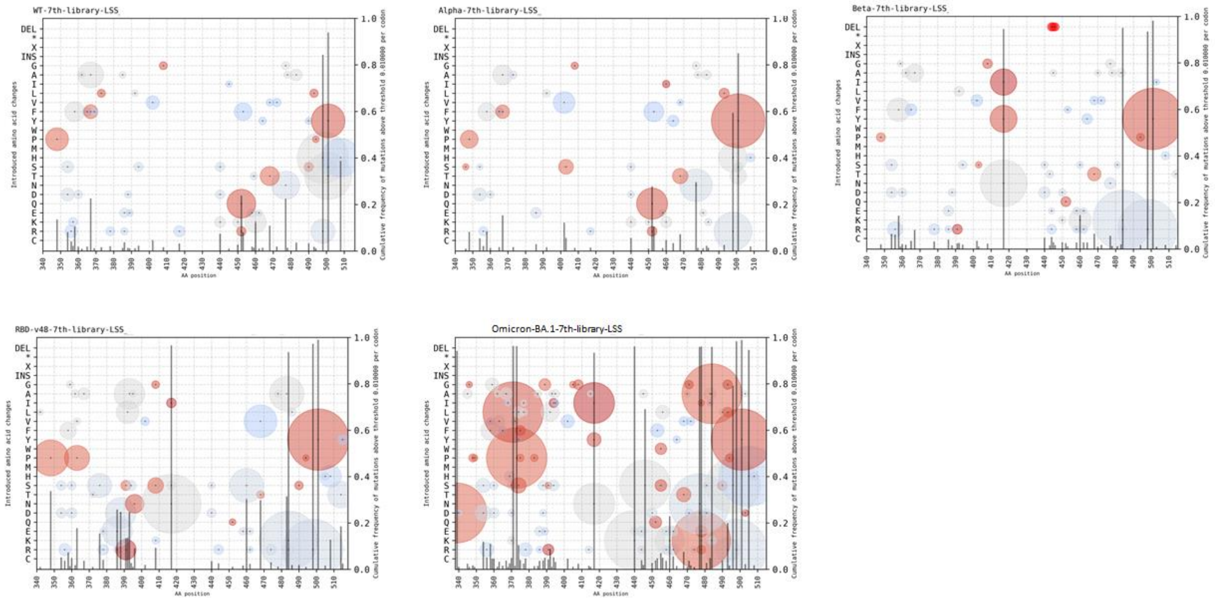

Figure 1 – Full RBD sequence mutation scatter plots of in vitro evolution under LSS analogous to Fig.3. The relative size of the circles reflects mutation frequency in the population, and color denotes amino acid functional conservation based on BLOSUM62 scoring matrix (how commonly is the original amino acid residue replaced with the other in conserved blocks of alignments across many species of the same protein). The corresponding NGS results, which cover the RBD regions outside of the RBM analyzed in the main text, are now provided as a new Supplementary Fig. S19 – S23 and made available in our online repository.

2. Effect of Sanger sequencing and low-frequency variants:

Sanger sequencing in our study was used primarily as a tool to monitor the acquisition of mutations and was not used for quantitative frequency estimation. We included this in Fig. 1D to make it clearer and also expanded the dataset with additional Sanger reads (Fig. S8 – S13) and clarified in the text that these sequences represent unique clones, many of which were obtained multiple times independently.

The additional reason for Sanger sequencing usage was to demonstrate mutations co-occurrence in individual sequences compared to frequency-based analyses from NGS. To further strengthen the comparison between sequencing methods, we revised our analysis to include the relative frequencies of unique NGS reads and now display the 30 most abundant reads, along with their absolute counts, for each library (see Fig. S14-S18). This establishes a clear parallel with the Sanger-based data and demonstrates that our conclusions are supported not only by endpoint frequencies but also by the co-occurrence patterns of key mutations.

Together, these additional experiments and analyses provide robust evidence that the conclusions regarding the dominance of HSS and the rapid fixation of adaptive mutations are independent of the specific library design and sequencing methodology.

Numbers on the x-axis of Fig. 3C-D are not legible

Fig. 3 was completely revised with numerous updates. All axes and labels are now legible and clearly interpretable. In addition, a new panel (3F) was added to analyze the relationship between ACE2 affinity selection and antibody escape requested by other reviewer, using data from neutralizing antibody binding in convalescent serum (doi.: 10.1016/j.chom.2021.02.003).

Seems that authors used NGS to analyse original library composition but then used only Sanger sequencing to determine mutations that get selected after each selection round? The number of clones that are sequenced after each selection round seems to differ and vary between libraries, why is that how was the number of clones to be sequenced determined? If only Sanger sequencing was done instead of some form of deep sequencing does that mean the data likely underestimates lower frequency variants?

Two complementary sequencing approaches were used in this study. Sanger sequencing was employed to monitor the progress of *in vitro* evolution and to assess the co-occurrence of mutations. Only unique clones identified are reported in the manuscript, although many sequences were observed multiple times. During revision, additional Sanger sequences were included to increase the representativeness of the data. To further complement this, we performed an analysis of the most abundant reads from NGS data, providing a parallel assessment of clone frequency (now Fig. S14-S18).

Next-generation sequencing (NGS) was used to obtain a detailed frequency-based view of the evolving libraries, as indicated in Fig. 1D. For HSS, multiple NGS analyses were performed to capture the rapid fixation of beneficial clones (Fig. S15 – S18 and S24 – S35).

For LSS, NGS was performed at the seventh round, guided by Sanger data showing substantial variability and a mutation count comparable to the fourth round of HSS. This fact is now explained in the manuscript – lines 249 - 253. However earlier LSS rounds were also sequenced using the Sanger method (Fig. S8 – S13). NGS for earlier LSS rounds was deemed unlikely to provide additional insights, as it would primarily reflect a lower mutational load. Manuscript text:

(lines 212-216): Selected libraries (Fig. 1D) were analyzed by next generation sequencing (NGS-Illumina paired-end deep sequencing 2 × 250 bp) while all rounds were monitored by Sanger sequencing. This combined strategy ensures that both high-frequency and relevant co-occurring mutations are accurately captured, while low-frequency variants are considered in the NGS analysis

(lines 241-245): For deep sequencing analysis, we selected the unsorted, 2nd and 4th rounds of HSS). For comparison, the 7th round of LSS) was chosen to obtain a comparable number of mutations in the selected sequences as can be seen from Sanger sequencing results and Illumina sequencing reads analyses (Fig. S7-S18).

Authors say that in RBD-v48 library "increased affinity provided a "surplus" of affinity providing broader mutational tolerance, allowing the fixation of unique mutations such as A348P, A363P, K386E, N388D, N460S, I468V, V483A, and S514T identified by NGS". I do not see where this data is shown.

The data supporting this statement have been added in Fig. S22 and similarly extended for the other LSS libraries to cover the full mutagenized region (Fig. S19-S23). Consistent with the Sanger sequencing results, NGS confirms that in LSS this region did not show fixation of mutations to high frequency as discussed in the comment: Influence of library design.

I'm not sure that this figure reference is correct "The display of RBD variants with and without this mutation revealed a striking difference, indicating its dramatic structural and functional impact (Fig. S1)". Fig S1 shows gating strategy and Fig. S11 shows disappearance of S375F from BA.1, I'm not sure what the authors are referring to in tat sentence.

The figure reference in that sentence was incorrect and has been corrected to Fig. S2.

While authors say BLOSUM62 matrix is commonly used, I doubt the majority of people know what it is and this should be explained.

The BLOSUM62 matrix reflects measure of evolutionary conservation of any amino acid residue being (eventually) replaced in an aligned conserved protein block of a multi-species multiple-sequence alignment with any other amino acid residue. The values (weights) were indeed calculated from well-conserved regions of some proteins of a broad spectrum of organisms. In such multiple-sequence alignments are sometimes present more or less common replacements. Their probabilities were transformed into weights by original authors of BLOSUM matrices. We just took over the final matrix with the weights to emphasize, which amino acid changes are not common (supposedly functionally drastic) from those which occur rather frequently (supposedly functionally similar). The matrix is now better explained in the Fig. 3 caption and in the Discussion, lines 635-638 and Methods section 891 - 897, where we also describe how colors were assigned. Additionally, the colors dynamically calculated and used to render the figures are provided in the source data .tsv file deposited at Zenodo.org under DOI:10.5281/zenodo.17252728 accession.

Authors should discuss some limitation of using RBD only system, e.g. we know ACE2 binding is affected by RBD up down movement, indeed S375F mutation that was selected against in this study is well described as mutation that leads to RBD more in the down position (https://doi.org/10.1038/s41467-022-32665-7, https://doi.org/10.1101/2021.12.21.473620).

We added this important limitation in the Discussion section.

(lines 607 – 623): It should be noted that the use of the isolated RBD domain for *in vitro* evolution has limitations, as it may lack stabilizing contacts present in the full Spike trimer that can compensate for the destabilizing effect of the S375F substitution. Structural studies have shown that S375F favors the RBD adopting the "down" conformation [69-71], where substantially more intermolecular contacts are formed. Nevertheless, the overall stability of the Omicron Spike, at least in the initial inflection region, remains markedly reduced [72]. Sanger sequencing of individual LSS rounds revealed minimal fixation of mutations outside the RBM region (amino acids 336–430). In later rounds (≥3), partial fixation of the stabilizing mutations A348P and I358F was observed (with I358F being used also as a starting mutation in HSS) [25,73]. Overall, these results suggest that this portion of the RBD without destabilizing Omicron mutations has a low impact on the acquisition of mutations within the RBM. Therefore, the presence of the S375F substitution, which is largely responsible for the Omicron phenotype [39], must be supported by complex epistatic effects underlying immune escape or tissue tropism switching. These effects likely confer a positive fitness advantage but cannot be directly recapitulated in our experimental system. It should also be noted that despite S375F being fixed in HSS (where only the RBM was mutated), its thermostability reverted rapidly to WT values, due to secondary mutations (Fig. S39).

Reviewer #1 (Remarks on code availability):

Code is available with detailed README. I did not try to rerun the analysis.

Reviewer #2 (Remarks to the Author):

This study employs an enhanced yeast surface display method to identify mutations in SARS-CoV-2 RBD/RBM that maintain or increase binding affinity for ACE2. Selections of mutants are performed under low (LSS) and high stringency (HSS) conditions for ACE2 binding. Under HSS conditions some mutations are more highly represented than under LSS conditions, and the authors report the HSS mutants to be higher affinity for ACE2. Many of these higher affinity HSS mutations are found in SARS-CoV-2 sequences in the GISAID database and in Omicron variants. The main findings of the study are that high stringency selection conditions favor rapid fixation of mutants for the selected phenotype, and that mutations selected for enhanced ACE2 binding are very common in SARS-CoV-2 Omicron variants.

The study is robust and well designed. The authors use a range of starting variants for their screens, in addition to Wt, and employ alternating selection plasmids to enhance reliability. The starting libraries are good quality. The data on HSS vs LSS fixation is convincing. Many of the mutations selected for highest ACE2 affinity binding are already known to enhance ACE2 binding affinity, supporting the validity of the selection protocol for ACE2 binding. Some of these HSS mutants are also directly assayed for ACE2 affinity, though this should be extended to a wider range of mutants.

It is known that Omicron variants exhibit increased affinity for ACE2 and possess mutations that increase ACE2 affinity. The present study confirms that mutations selected for increased ACE2 affinity are commonly found in Omicron variants. Whilst the study provides a very good demonstration of the power of their yeast display method to identify mutations leading to increased ACE2 affinity, the key findings of the study are not in themselves particularly novel.

The manuscript promotes the idea that ACE2 affinity is the main driver of Omicron evolution. However, this has not been tested or proven in the current study. The finding that mutations selected for increased ACE2 affinity are present in SARS-CoV-2 is not the same as showing ACE2 affinity is the main driver of Omicron evolution, rather it confirms ACE2 affinity as an important component of Omicron phenotype.

Response to Reviewer #2:

We appreciate this constructive comment, which prompted us to expand and improve the manuscript to include additional information addressing the role of immune escape in shaping Omicron evolution. Since our primary interest lies in the emergence of Omicron, we incorporated the comprehensive dataset generated by Allison J. Greaney and colleagues (Prof. Jesse D. Bloom laboratory), which quantifies escape from prior Omicron convalescent serum and thus reflects antibody-driven selective pressures present in the population at that time.

We compared these immune escape scores with both the mutation frequencies in the GISAID database and those obtained from our LSS and HSS selections (Fig. 3F). Although each dataset is derived from distinct experimental or evolutionary selection pressures and carries its own bias, their comparison provides a meaningful qualitative framework for deconvoluting the potential selective forces contributing to the fixation of individual mutations. Importantly, the convergence of mutations selected under high-stringency ACE2 binding conditions with those fixed in Omicron variants, despite the availability of numerous alternative substitutions conferring higher immune escape potential, suggests that ACE2 affinity was not a coincidental

correlate but a dominant selective constraint (see middle and right panels Fig. 3F). Moreover, plotting mutation frequencies of CoV-2 lineages against either mutation frequencies after *in vitro* evolution driven by escape from convalescent serum, HSS or LSS shows a clear correlation only with HSS. This observation implies that during the early emergence of Omicron, adaptive advantages conferred by improved receptor engagement outweighed the benefits of immune evasion alone. The integration of affinity and immune escape datasets thus provides mechanistic evidence supporting our conclusion that enhanced ACE2 binding represented a principal evolutionary driver in the initial phase of Omicron diversification as no neutralizing antibodies were targeting Omicron mutant residues.

These results are now presented in Fig. 3F, with full annotation available in the online data repository. The corresponding analysis is described in the main text:

(Results section, lines 411–425): Our experimental design excluded antibody selection. However, we were able to reuse complementary datasets from studies specifically focused on antibody escape, in which ACE2 binding was not a stringent constraint. This allowed us to deconvolute mutations primarily driven by receptor affinity from those enriched due to immune escape. Here, we utilized the comprehensive frequency data generated by Allison J. Greaney and colleagues (Prof. Jesse D. Bloom laboratory, 24) and compared them with our experimental results and frequencies observed in circulating viral populations recorded in GISAID. The combined analyses are presented in Fig. 3F, with an annotated version available at [https://host-patho-evo.github.io/mutation_scatter_plot/]. The plot shows that the stronger selected mutations in HSS are also more frequent in SARS-CoV-2. In addition, this group of mutations has also some neutralizing potential, which may be due to them being within the ACE2 binding site, which is highly enriched for neutralizing mutations. This comparison identified V445P as the mutation under strongest antibody-mediated selection. By contrast, L452M and E484R were predominantly enriched due to enhanced ACE2 binding. For Q498R and N501Y, both antibody escape and receptor-binding advantages act synergistically to promote their selection.

(Discussion, lines 641–653): Comparing our results with an existing dataset mapping how mutations affect recognition by polyclonal human plasma antibodies 24 showed that residues selected under HSS most closely matched the mutation frequencies observed in GISAID (Fig, 3F). A subset of selected mutations exhibited dual characteristics with enhancing both receptor binding and immune escape (e.g., N460K, S477N, Q498R, N501Y). In contrast, other positions showed that residues conferring stronger immune escape, such as E484F, were not selected. This suggests that, at these sites, selection favored residues tolerated in terms of receptor binding yet providing a partial immune escape advantage, as exemplified by E484A. This finding is particularly important for applying similar methodologies to predict advanced viral lineages and mutations in scenarios where a large portion of the human population is no longer immunologically naïve. It is evident that immune escape mutations will preferentially emerge from variants that balance receptor binding affinity and immune evasion which are represented in our experiments as the low frequency mutations.

> *Figure 4 - affinity differences between LSS clones vs HSS clones are not clear as LSS clones are shown on a more compressed X-axis than HSS clones, the same dimension axes for panels A and C are required to compare clones in this figure. The effects of HSS and LSS on clone affinity (Figure 4C and Table S7) should be more comprehensively and accurately described. The average affinity gain of the LSS clones is actually higher than the HSS clones (Table S7). Measurement of affinity gain in more LSS and HSS clones would allow a more robust conclusion about the effects of LSS vs HSS on affinity to be drawn.*

The entire experiment described in Fig. 4 was repeated and the figure itself, was completely redone. The original version combined results obtained by students from the two participating laboratories. However, control measurements revealed a systematic difference between the results obtained by the two laboratories, particularly in the absolute affinity values (not the

relative to WT values). This is common for affinity measurements done using different instruments. To eliminate this bias, the entire dataset, further expanded to include additional mutations (including from earlier *in vitro* evolution steps), was remeasured in a single laboratory by a single student under identical conditions. The results, supported by three independent replicates, clearly demonstrate a significant affinity difference between the LSS and HSS libraries, consistent with the expected trend which has been also shown in the original manuscript.

Some of the figures could be clearer. Figure S13 should have the mutations introduced to create the variants BA.1 and BA.2 colored yellow for those variants.

Figures and their captions were corrected. Mutations corresponding to relevant variants (Alpha, Beta, RBD v48, BA.1 and BA.2 are always marked and shown where relevant, see Fig. S8-S18).

In Figure S5 it is not immediately clear which mutations have been selected by HSS and which are present because they were in the starting variant, e.g. N440K is already present in BA.1 and BA.2. This should be clarified by use of a different color for such mutations. The superscript in the table legend should also be checked.

Figures and their captions were corrected

For analysis of convergence between in vitro and virus evolution it would it be valuable to compare frequencies of co-occurring mutations in the GISAID database with mutation co-occurrence in clones from HSS.

To illustrate the co-occurrence of mutations, we have added an analysis of Illumina sequencing reads and their frequencies, which provides a meaningful parallel to both Sanger sequencing and data available from the GISAID database (Fig. S8-S18). From these data, the frequencies of mutation co-occurrences can be readily calculated and compared with those observed in circulating viral sequences. New line with GISAID cooccurrence data for positions 498 + 501 was added to Table S6.

Further analysis did not reveal any novel or informative patterns in RBM region beyond those already discussed in the manuscript, and therefore we did not include this additional section in the main text.

Reviewer #3 (Remarks to the Author):

This work builds upon previous work from the same group in 2021 (Zahradnik et al, 2021) where they performed in vitro evolution and affinity maturation of the SARS-CoV-2 RBD towards ACE2 and ended up with a mutant RBD with 600-fold increase in binding affinity. Some of the mutations that they evolved were also observed in Omicron.

That study had certain limitations, the codons were optimized for expression in Saccharomyces cerevisiae for yeast display and in vitro evolution included a pre-equilibrium selection step favoring faster on-rates, which may not align with natural viral evolution. Thus, they redesigned/improved their study in this paper.

This paper is actually split in two major parts. In the first one (which is most of the paper), the authors study how in vitro evolution can be used to identify SARS-CoV-2 mutations that increase binding affinity to ACE2, while maintaining structural integrity. They compare their results with mutations that occurred in real SARS-CoV-2 strains. This is actually used as a proof of concept to study how ACE2 binding can be increased in SARS-CoV-1 related sequences, in the second part of the paper (this is only a small section at the end of results). They identified as sufficient to close the binding affinity gap between SARS-CoV-1 and SARS-CoV-2. This is a very important finding. At the same time, it's actually very close to a gain of function experiment on how to significantly increase the binding affinity of a group of Sarbecoviruses (the SARS1 group) that have much lower infectivity at this time, but significantly higher mortality. SARS-CoV-1 had a mortality rate of ~10% whereas SARS-CoV-2, at the begining had a mortality rate bellow 1% and this further decreased. Basically, this experimental evolution framework is mostly useful for identifying mutations that will allow a virus to break the species barrier and start infecting successfully humans. It is not optimized for capturing the subsequent evolutionary trajectory of the virus, that will need to evade immune response, re-infect and also change tissue tropism in order to facilitate more easy spread (preferentially moving to the upper respiratory system), in the next phases of an epidemic.

In my opinion, the most important finding of this work is how easy it actually is for a member of the SARS-CoV-1 evolutionary group to significantly increase its infectivity and start a new epidemic (like that of 2002-2004 SARS outbreak) that at the early stages may also have a significantly increased lethality (possibly ten times higher than SARS-CoV-2 had at the begining). The implications are very significant. The scientific community needs to remain vigilant and continue research on Coronaviruses. Both in terms of monitoring and in terms of rapidly developing safe and effective vaccine technologies. In my opinion, RNA vaccines are the way forward, for rapid development, in order to combat any new epidemic, but safety issues need to be addressed as well. And other more conserved regions for a pan-Sarbecovirus vaccine need to be further investigated. At the same time, it shows that a new epidemic with a SARS-1 related sarbecovirus may easily evolve in nature, without the need for a lab-leak. Although, given the level of natural immunization that the human population has aquired in the last 5 years for SARS-CoV-2, it is possible that a new SARS-CoV-1 related epidemic may not be as bad (assuming significant cross-reaction). Furthermore, this work also shows how close the world was in 2002-2004 for SARS to evolve into a much more transmissible virus and generate a pandemic like that of 2019. The immediate response of the people that were involved in containing the 2002-2004 SARS outbreak was crucial in averting a catastrophic pandemic then. Thus, this highlights the importance of rapid response, collaboration, openess, sharing of information and coordinated action at the international level. My recommendation is to significantly reformat the paper accordingingly, in order to properly discuss all the above very significant findings and their even more important implications.

Response to Reviewer #3:

We sincerely appreciate the reviewer's insightful and highly valuable comments. We are very pleased that the reviewer has precisely recognized the main motivation and conceptual framework of our work. We fully agree that the findings from the SARS-CoV-1 part of the study have important implications regarding the potential of SARS-CoV-1–related sarbecoviruses to increase their affinity for human ACE2 and possibly cross the species barrier.

At the same time, we would like to emphasize that the first part of the manuscript, to which the reviewer refers only briefly, is in our view equally essential. It establishes the methodological foundation and conceptual context that make the SARS-CoV-1 findings meaningful. In particular, it validates the *in vitro* evolution approach against real-world SARS-CoV-2 sequence data, thereby providing a robust framework for interpreting the subsequent SARS-CoV-1 results. Moreover, this section also explains newly observed phenomena, such as the absence of synonymous mutations in BA.1 and BA.2 lineages. SARS-CoV-2 is the most sequenced virus ever, which was done in real-time, providing a unique resource to study viral evolution. The fact that using *in vitro* evolution under certain HSS but not other (LSS) conditions, provides a solid foundation to study potential evolution of other viruses, which is the main objective of this paper.

We fully agree that the broader implications for public health and pandemic preparedness are highly significant. Accordingly, we have expanded the Discussion to better highlight these aspects, emphasizing both the scientific and societal importance of our findings:

(Discussion lines 685-701): Strikingly, closing the affinity gap between SARS-CoV-1 and SARS-CoV-2 for ACE2 binding, which has been linked to the lower infectivity of SARS-CoV-1 23, requires only a single substitution, Y442S. In addition, amino acid residues that share the same binding mode in both SARS-CoV-1 and SARS-CoV-2 exhibit a notable degree of convergence in our selection experiments. This similarity suggests potential cross-reactivity of immune responses targeting these sites, thereby constraining the possible adaptive trajectories. Overall, this experimental evolution framework provides a powerful strategy for identifying mutations that could enable a virus to cross the species barrier and efficiently infect humans. It also highlights mutations whose careful surveillance should be prioritized. Comparable in vitro evolutionary analyses performed under different selective pressures could help dissect the subsequent evolutionary processes that drive immune escape, or shifts in tissue tropism toward the upper respiratory tract 15 80, features characteristic of later epidemic stages, and ultimately advance our understanding toward true predictability of viral evolution. Ultimately, the lessons from both the 2002–2004 SARS outbreak and the COVID-19 pandemic highlights the critical importance of early detection, transparency, data sharing, and international collaboration in preventing and mitigating future pandemics.

Finally, we would like to note that we are currently preparing an extended study specifically focused on predicting potential affinity-enhancing, species-barrier-crossing mutations across a broader spectrum of coronaviruses. For this reason, we have chosen not to further expand the SARS-CoV-1 section in the present manuscript. We are confident that the reviewer will appreciate and understand our decision.

The authors also need to discuss further the weaknesses of this technology in terms of predicting the next phases of an epidemic. The fitness of the virus is a combination of increased transmissibility and immune evasion, while still retaining structural integrity. Increased transmissibility is in turn a combination of better binding to ACE2 and tissue tropism to the upper respiratory system, that allows it to spread more easily. While this experimental/in-vitro evolution setting can capture/explain the binding of the virus to ACE2, it does not account for tissue tropism and for immune evasion (see and discuss DOI: 10.3390/v14040707).

In response to this valuable comment, we have included multiple changes in the manuscript and integrated our results with immune escape dataset generated by Allison J. Greaney and colleagues (Prof. Jesse D. Bloom laboratory), which quantifies escape from prior Omicron convalescent serum and thus reflects antibody-driven selective pressures present in the population at the beginning of 2021 (Fig. 3F). Manuscript text:

(Results section, lines 411–425): Our experimental design excluded antibody selection. However, we were able to reuse complementary datasets from studies specifically focused on antibody escape, in which ACE2 binding was not a stringent constraint. This allowed us to deconvolute mutations primarily driven by receptor affinity from those enriched due to immune escape. Here, we utilized the comprehensive frequency data generated by Allison J. Greaney and colleagues (Prof. Jesse D. Bloom laboratory, 24) and compared them with our experimental results and frequencies observed in circulating viral populations recorded in GISAID. The combined analyses are presented in Fig. 3F, with an annotated version available at [https://host-patho-evo.github.io/mutation_scatter_plot/]. The plot shows that the stronger selected mutations in HSS are also more frequent in SARS-CoV-2. In addition, this group of mutations has also some neutralizing potential, which may be due to them being within the ACE2 binding site, which is highly enriched for neutralizing mutations. This comparison identified V445P as the mutation under strongest antibody-mediated selection. By contrast, L452M and E484R were predominantly enriched due to enhanced ACE2 binding. For Q498R and N501Y, both antibody escape and receptor-binding advantages act synergistically to promote their selection.

(Discussion, lines 641–653): Comparing our results with an existing dataset mapping how mutations affect recognition by polyclonal human plasma antibodies 24 showed that residues selected under HSS most closely matched the mutation frequencies observed in GISAID (Fig, 3F). A subset of selected mutations exhibited dual characteristics with enhancing both receptor binding and immune escape (e.g., N460K, S477N, Q498R, N501Y). In contrast, other positions showed that residues conferring stronger immune escape, such as E484F, were not selected. This suggests that, at these sites, selection favored residues tolerated in terms of receptor binding yet providing a partial immune escape advantage, as exemplified by E484A. This finding is particularly important for applying similar methodologies to predict advanced viral lineages and mutations in scenarios where a large portion of the human population is no longer immunologically naïve. It is evident that immune escape mutations will preferentially emerge from variants that balance receptor binding affinity and immune evasion which are represented in our experiments as the low frequency mutations.

We added discussion of the limitations of our system (Discussion section, lines 607 – 623), as well as possible modifications (lines 694 – 696). As the reviewer correctly notes, uncovering some of these phenomena is not feasible with the current approach and would require the application of complementary techniques.

> *At the early stages of the pandemic, where a large portion of the human population was still immunologically naïve, increased binding to the human ACE2 ortholog was very important. Nevertheless, in later stages, immune evasion and increased infectivity via the upper respiratory became more important. For example, almost all VoCs had not only increased binding to ACE2, but also increased immune escape and even increased re-infection rates. More specifically, the Omicron lineage demonstrated a re-infection rate 5.4 times higher than that of the already highly infectious Delta lineage. However, this increase in infectivity is not only due to higher affinity of the Spike RBD for the human ACE2 receptor. Pseudovirus assays demonstrated that the Omicron's RBD binding affinity for the human ACE2 receptor is 2.4 times higher than that of the Wuhan-Hu-1 strain (see DOI:10.1038/s41586-021-04386-2), which is lower than that of Alpha (6.2 times higher than Wuhan-Hu-1), similar to that of Beta (2.4 times higher than Wuhan-Hu-1), and higher than that of Delta (1.2 times higher than Wuhan-Hu-1). The high infection and re-infection rate of Omicron might be attributed, at least in part, to strong pressure to achieve antigenic shift, because it has emerged in an environment where many individuals already had been infected by other variants or were immunized to a large extent by Spike-targeting vaccines. The Omicron lineage could already escape 26*

out of 29 monoclonal antibodies (see DOI:10.1038/s41586-021-04386-2) that target the Spike RBM. Furthermore, its tissue tropism changed from the lower to the upper respiratory system, and played a major role in increasing its overall infectivity compared to Delta. Thus, an in vitro system that focuses on optimizing the binding affinity of the Spike to ACE2 cannot account for the epistatic effects of immune-escape and tissue tropism switching.

We fully agree with the reviewer that our current methodology cannot, in its present form, capture the epistatic effects underlying immune escape or tissue tropism switching, and that overall viral fitness represents an exceedingly complex, multifactorial process. The results we present primarily demonstrate how markedly different outcomes can emerge under distinct selective pressures, revealing the fundamental determinants of RBM shaping. Still, as we show in the new Fig. 3F, HSS is the only method that captures high mutation frequencies observed during viral evolution, including the evolution of Omicron. Moreover, we showed in Table S5 that part of the late evolution of SARS-CoV-2 (from 2023 and onwards) can also be explained by retaining the potential to bind tightly to ACE2 (as shown by HSS). We detailed the convergent and divergent evolutionary trends observed under LSS, HSS and in SARS-CoV-2 in lines 411-425.

(lines 411-425): Our experimental design excluded antibody selection. However, we were able to reuse complementary datasets from studies specifically focused on antibody escape, in which ACE2 binding was not a stringent constraint. This allowed us to deconvolute mutations primarily driven by receptor affinity from those enriched due to immune escape. Here, we utilized the comprehensive frequency data generated by Allison J. Greaney and colleagues (Prof. Jesse D. Bloom laboratory, [24]) and compared them with our experimental results and frequencies observed in circulating viral populations recorded in GISAID. The combined analyses are presented in Fig. 3F, with an annotated version available at [https://host-patho-evo.github.io/mutation_scatter_plot/]. The plot shows that the stronger selected mutations in HSS are also more frequent in SARS-CoV-2. In addition, this group of mutations has also some neutralizing potential, which may be due to them being within the ACE2 binding site, which is highly enriched for neutralizing mutations. This comparison identified V445P as the mutation under strongest antibody-mediated selection. By contrast, L452M and E484R were predominantly enriched due to enhanced ACE2 binding. For Q498R and N501Y, both antibody escape and receptor-binding advantages act synergistically to promote their selection.

We agree that tissue tropism switching is largely driven by mutations outside the RBM, for example near the furin cleavage site (e.g., P681R/H, N679K) or within the S2 domain (e.g., N764K, D796Y, N856K, Q954H, N969K, L981F). Our in vitro evolution framework focuses specifically on receptor-binding domain adaptation, and therefore intentionally isolates the selective forces acting on receptor engagement rather than those governing post-entry events, immune evasion, or altered tissue tropism. To our mind, it is amazing that even such a simplistic model, as used by us in this work, can explain the majority of SARS-CoV-2 mutations in the RBM (which is the highest mutating region in the virus). This shows the crucial importance of ACE2 binding, and the unique mechanism of evolution driving Omicron appearance.

Nevertheless, discussion highlighting the limitations of our system and its inability to capture epistatic effects of immune escape and tissue tropism switching has been added to the revised manuscript and is discussed with the effect of S375F mutation (lines 597 – 610).

In Introduction, the authors discuss the evolutionary rate of SARS-CoV-2. Evolutionary analyses estimate a mutation rate of 0.5 × 10−3–1.1 × 10−3 substitutions/site/year, which translates to approximately 1.3–2.8 substitution/month for the entire genome. At

the same time, the Spike region mutates almost 5 times faster than the other regions of SARS-CoV-2 (see and discuss DOI: 10.3390/v14040707). In addition, the same comparative study revealed that the amino acid substitutions of Spike were higher in the successful VoCs, compared to the other SARS-Cov-2 sequences.

The authors also have a very interesting section and discussion on the emergence of Omicron. It is noteworthy that each of the five major VoCs was not derived by a few extra mutation steps of another pre-existing VoC. This is possibly because steady-stepwise evolution may not be sufficient to achieve immune escape during the evolution of a pandemic where the vast majority of the population is no longer antigenically naïve (especially for the Spike ORF), but saltatory evolution may be necessary (see DOI:10.1073/pnas.1909031116). It is conceivable that "under-the-radar" lineage evolution may be occurring, involving a combination of host-switching and chronic infections of immune-compromised patients. Although the authors discuss the scenario involving immunocompromised patients, this alone cannot explain the intense localization of amino acid changes in the spike of Omicron. It possible that a more complex scenario of host switching accelerated changes at the Spike, followed by infection of an immunocompromised patient, that gave ample time for re-adaptation of that strain back to humans. See DOI: 10.1093/infdis/jiab622, and DOI: 10.3390/v14040707).

Several comparative studies have identified recurring mutations (homoplasies/convergent evolution) in successful VoCs (see DOI: 10.1016/j.meegid.2020.104351, DOI: 10.1073/pnas.2104241118 and DOI: 10.3390/v14040707). The authors need to discuss in more detail how many of these particular recurring mutations were identified in their experimental evolution framework

We appreciate the reviewer's detailed discussion of evolutionary phenomena and variant emergence. Relevant passages, additional citations, and expanded discussion have been incorporated throughout the manuscript, particularly in the Introduction (lines 49 – 53) and Discussion (page 18), to address the points raised regarding mutation rates, Spike evolution, and recurring mutations identified in our experimental evolution framework.

Specific changes:

(Introduction, lines 49 – 53): The mutation rate of SARS-CoV-2 is estimated to be 10^{-6} mutations per nucleotide per replication cycle representing 1.3–2.8 substitution/month for the entire genome, similar to other beta coronaviruses [4]. Yet, the distribution of mutations is not uniform and is highly context-dependent, influenced by local sequence composition, genomic region, and RNA structure and refined by functional selection [5].

(Discussion, line 592-595): Notably, key mutations such as N440K, L452R, N460K, S477N, T478K, Q498R, and N501Y were enriched under HSS, mirroring their individual convergent evolution in multiple VOCs and combined emergence in Omicron and its descendant SARS-CoV-2 lineages.

(Discussion, line 661-667): Omicron did not emerge through stepwise evolution in the general public, but through a drastic shift in the mutation landscape as an example of saltatory evolution [77] which was as we demonstrate in this manuscript driven by HSS likely within a small closed environment (maybe in immunocompromised patients). Our data indicate that rapid and dramatic accumulation of context-specific beneficial mutations can occur solely under strong selective pressure for the relevant trait, without the need for any additional mechanism that would increase the intrinsic mutation rate.

Some minor corrections:

In vitro: Please italicize throughout the manuscript

Corrected

In silico: Please italicize throughout the manuscript

Corrected

Typo: Predominantly immune evasion associated mutations were not selected in vitro.

We reformulated the whole sentence: Mutations primarily associated with immune evasion were not selected.

I would also recommend that the authors change the title of the paper, it doesn't read well as it is.

We changed the manuscript title to: "Stringent Selection Drives Convergence Toward Omicron-like SARS-CoV-2 Receptor-Binding Motifs" as it indeed matches better our (still limited) findings.

Reviewer #3 (Remarks on code availability):

Its fine.
