# Supplementary material for: Author Correction: Stringent selection drives convergence toward omicron-like SARS-CoV-2 receptor-binding motifs
Source: Nat Commun. 2026 Aug 26;17:9058. doi: 10.1038/s41467-026-77179-8 (PMC13518779; doi:10.1038/s41467-026-77179-8)
Supplement: Supplementary file 1 [file 41467_2026_77179_MOESM1_ESM.pdf]

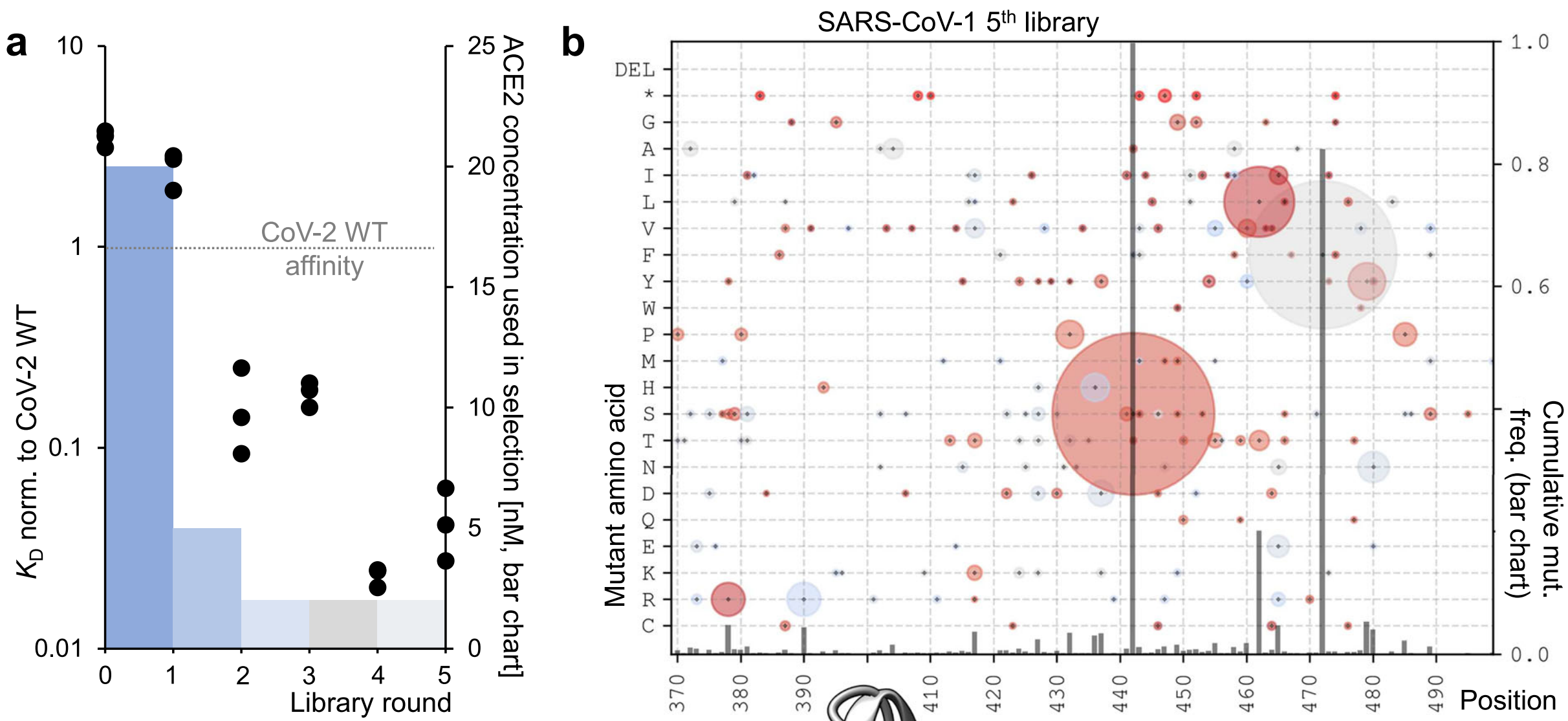

c

CoV-1 Pos.	CoV-1 WT res	CoV-1 affi. mat.	CoV-2 Pos.	CoV-2 WT res	In CoV-2 var	CoV-2 affi. mat.
432	S	P	445	V	P, H	P
436	Y	H	449	Y	-	-
437	N	D	450	N	D	D, K
442	Y	S	455	L	F, S	M, S
462	P	L	475	A	V	-
472	L	F	486	F	S, V, P	V
479	N	Y	493	Q	R	G, R, K
480	D	N	494	S	P	-
485	T	P	499	P	-	-

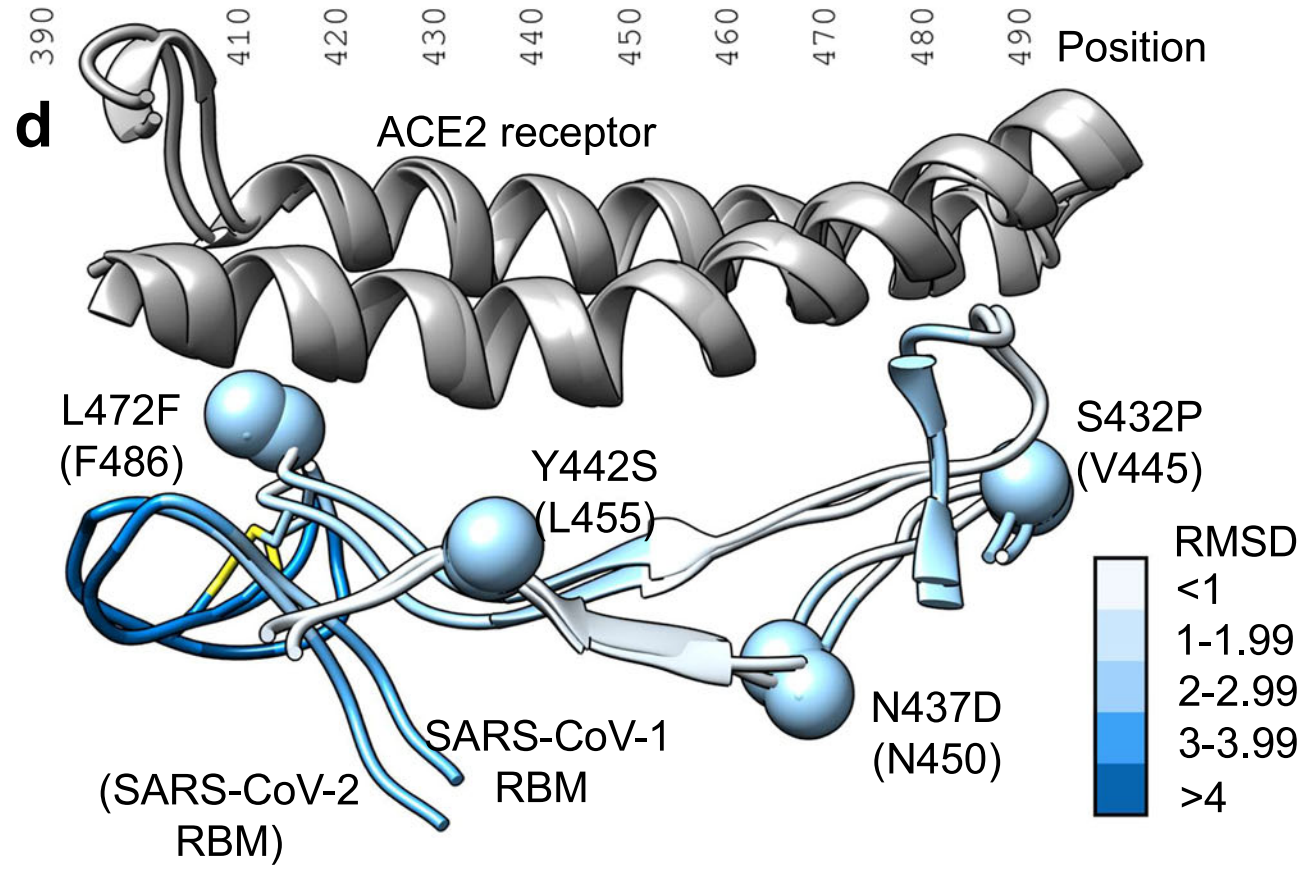

Fig. 6 | SARS-CoV-1 selection for high affinity binding of human ACE2. **a** Progression of affinity maturation across individual libraries (1–5) (right axis, bar chart). Black points (left axis) represent K_D values normalized to SARS-CoV-2 affinity for SARS-CoV-1 (library round 0, triplicate) and three randomly selected clones after completed selection library step (dots at the right edge of each bar). The sequences of these clones are shown in Fig. S40. **b** Mutation scatter plot showing the distribution of mutations in the 5th library above 0.1% threshold. The legend matches that of the mutation scatter plot in Fig. 3, where the radius of the circle represents mutation frequency in the population, and the color indicates the evolutionary distance from the original amino acid. **c** Selected residues during affinity maturation of SARS-CoV-1 and SARS-CoV-2, along with an analysis of the structural similarity of their receptor-binding motifs (RBMs) in binding to angiotensin converting enzyme 2 (ACE2) peptidase domain. Residues showing evidence of convergence are highlighted in red and are depicted as spheres in the structure comparison. **d** based on structures ACE2 and SARS-CoV-1 (PDB: 6CS2[65]) and ACE2 and SARS-CoV-2 (PDB: 6m17[66]). Amino acid residue number of SARS-CoV-1 with original and mutated AA residue is enlisted first and followed by residue number from SARS-CoV-2 (in braces with just the original AA residue shown, visualized in UCSF Chimera 1.18). Source data are provided in the file Source_Data_Fig-6.xlsx.
